# Two Novel Transcriptional Regulators Are Essential for Infection-related Morphogenesis and Pathogenicity of the Rice Blast Fungus *Magnaporthe oryzae*

**DOI:** 10.1371/journal.ppat.1002385

**Published:** 2011-12-01

**Authors:** Xia Yan, Ya Li, Xiaofeng Yue, Congcong Wang, Yawei Que, Dandan Kong, Zhonghua Ma, Nicholas J. Talbot, Zhengyi Wang

**Affiliations:** 1 State Key Laboratory for Rice Biology, Institute of Biotechnology, Zhejiang University, Hangzhou, People's Republic of China; 2 School of Biosciences, University of Exeter, Exeter, United Kingdom; University of Melbourne, Australia

## Abstract

The cyclic AMP-dependent protein kinase A signaling pathway plays a major role in regulating plant infection by the rice blast fungus *Magnaporthe oryzae*. Here, we report the identification of two novel genes, *MoSOM1* and *MoCDTF1*, which were discovered in an insertional mutagenesis screen for non-pathogenic mutants of *M. oryzae*. *MoSOM1* or *MoCDTF1* are both necessary for development of spores and appressoria by *M. oryzae* and play roles in cell wall differentiation, regulating melanin pigmentation and cell surface hydrophobicity during spore formation. MoSom1 strongly interacts with MoStu1 (Mstu1), an APSES transcription factor protein, and with MoCdtf1, while also interacting more weakly with the catalytic subunit of protein kinase A (CpkA) in yeast two hybrid assays. Furthermore, the expression levels of *MoSOM1* and *MoCDTF1* were significantly reduced in both *Δmac1* and *ΔcpkA* mutants, consistent with regulation by the cAMP/PKA signaling pathway. MoSom1-GFP and MoCdtf1-GFP fusion proteins localized to the nucleus of fungal cells. Site-directed mutagenesis confirmed that nuclear localization signal sequences in MoSom1 and MoCdtf1 are essential for their sub-cellular localization and biological functions. Transcriptional profiling revealed major changes in gene expression associated with loss of *MoSOM1* during infection-related development. We conclude that MoSom1 and MoCdtf1 functions downstream of the cAMP/PKA signaling pathway and are novel transcriptional regulators associated with cellular differentiation during plant infection by the rice blast fungus.

## Author Summary

*Magnaporthe oryzae*, the causal agent of rice blast disease, is an important model fungal pathogen for understanding the molecular basis of plant-fungus interactions. In *M. oryzae*, the conserved cAMP/PKA signaling pathway has been demonstrated to be crucial for regulating infection-related morphogenesis and pathogenicity, including the control of sporulation and appressorium formation. In this study, we report the identification of two novel pathogenicity-related genes, *MoSOM1* and *MoCDTF1*, by T-DNA insertional mutagenesis. Our results show that *MoSOM1* or *MoCDTF1* are essential for sporulation, appressorium formatiom and pathogenicity, and also play a key role in hyphal growth, melanin pigmentation and cell surface hydrophobicity. Nuclear localization sequences and conserved domains of the MoSom1 and MoCdtf1 proteins are crucial for their biological function. MoSom1 interacts physically with the transcription factors MoCdtf1 and MoStu1. We also show evidence that MoSom1 has the capacity to interact with CpkA, suggesting that MoSom1 may act downstream of the cAMP/PKA signaling pathway to regulate infection-related morphogenesis and pathogenicity in *M. oryzae*. Our studies extend the current understanding of downstream components of the conserved cAMP/PKA pathway and its precise role in regulating infection-related development and cellular differentiation by *M. oryzae*.

## Introduction

Eukaryotic organisms, including fungi, can sense and respond to extracellular cues via various signaling pathways for regulating a variety of developmental and differential cellular processes. Among these pathways, the conserved cyclic AMP-dependent protein kinase A (cAMP/PKA) signaling pathway has been well studied. The secondary messenger cAMP is universally produced through cyclization of ATP catalyzed by adenylate cyclases (ACs), and the level of cellular cAMP is regulated by cAMP phosphodiesterases [1], [2]. PKA consists of two catalytic subunits and two regulatory subunits. Binding of four cAMP molecules at two sites on each regulatory subunit causes conformational changes in PKA regulatory subunits, releasing activated PKA catalytic subunits which subsequently phosphorylate target proteins, including transcription factors, to control various physiological processes [3]–[6].

The cAMP/PKA response pathway plays an important role in fungal morphogenesis and virulence in plant pathogenic fungi [7]. During the last two decades, the function of several components of the cAMP/PKA pathway, in particular, AC and PKA, has been determined in a number of plant pathogenic fungi, including *Colletotrichum trifolii* [8], *C. lagenarium* [9], [10], *Fusarium verticillioides* [11], *Magnaporthe oryzae* [12]–[14], *Sclerotinia sclerotiorum* [15] and *U. maydis* [16], [17]. In yeasts, several downstream target proteins of PKA have also been identified and functionally characterized. In *Saccharomyces cerevisiae* for instance, the Flo8 transcription factor is critical for pseudohyphal growth in diploids, haploid invasive growth and flocculation and functions downstream of the cAMP/PKA pathway [18], [19]. A family of *FLO* genes, including *FLO11* (also referred as *MUC1*) which encodes a cell surface flocculin critical for both pseudohyphal growth and invasive growth, are regulated or activated by Flo8 [19]–[22]. It has been shown that the binding of Flo8 to the promoter of *FLO11* is regulated by Tpk2 (a catalytic subunit of PKA) in *S. cerevisiae* [23]. In both *S. cerevisiae* and *Candida albicans*, APSES (Asm1, Phd1, Sok2, Efg1, and StuA) transcription factors are targets for the cAMP/PKA pathway [24]–[27]. *C. albicans* Flo8 interacts with Efg1, a homolog of the Phd1/Sok2 and StuA proteins that regulate morphogenesis of *S. cerevisiae* and *Aspergillus nidulans*, respectively, and is essential for hyphal development and virulence [28]. In phytopathogenic fungi, several APSES transcription factors, including *F. oxysporum* FoStuA, *Glomerella cingulata* GcStuA and *M. oryzae* MoStu1 (Mstu1), have recently been identified [29]–[31]. Both GcStuA and MoStu1 are required for appressorium mediated plant infection [30], [31], while FoStuA is dispensable for pathogenicity by *F. oxysporum* [29]. In *U. maydis*, three transcription factors, Prf1, Hgl1 and Sql1, regulated by cAMP pathway have also been identified [32]–[34]. However, the downstream targets of the cAMP/PKA pathway still remain largely unknown in phytopathogenic fungi.

*Magnaporthe oryzae* is the causal agent of rice blast, the most destructive disease of rice worldwide [35], [36]. In the last two decades, *M. oryzae* has arisen as a model fungal pathogen for understanding the molecular basis of plant-fungus interactions [36]–[39]. It is now clear that infection-related morphogenesis is controlled by the cAMP response pathway and activation of the mitogen-activated protein kinase (MAPK) cascade in *M. oryzae* [12], [40]–[42]. Appressorium formation of *M. oryzae* requires the cAMP-response pathway, which responds to inductive signals from the rice leaf, including surface hydrophobicity and wax monomers from the plant [12]–[14], [43]–[45]. Deletion of the *M. oryzae MAC1* gene encoding adenylate cyclase resulted in mutants that cannot form appressoria and were defective in the growth of aerial hyphae and conidiation [12]. However, these defects in *Δmac1* mutants could be complemented by adding exogenous cAMP or by spontaneous mutations in the regulatory subunit of PKA gene *SUM1* [44]. Consistent with this, *M. oryzae CPKA*, which encodes the catalytic subunits of PKA, is dispensable for appressorium formation, but is required for appressorial penetration [13], [14]. Additionally, the role of the *M. oryzae* Pmk1 MAPK pathway in regulating appressorium development has been clearly established [40], [42], [46]–[49]. Therefore, the cAMP/PKA pathway and Pmk1 MAPK cascade are essential for regulation of appressorium development and pathogenicity in the rice blast fungus.

In *M. oryzae*, the upstream activation of adenylate cyclase appears to be mediated by G-proteins in response to physical and chemical properties of the rice leaf surface. The *M. oryzae* genome contains three Gα (MagA, MagB, and MagC), one Gβ (Mgb1), and one Gγ (Mgg1) subunits. For the three Gα subunits, only disruption of *MAGB* can significantly reduce vegetative growth, conidiation, appressorium formation, and pathogenicity, although the *ΔmagC* mutants are also reduced in conidiation [50]. MagB may respond to surface cues to stimulate Mac1 activity and cAMP synthesis, because expression of a dominant active allele of *MAGB* causes appressoria to form on hydophilic hard surfaces [51]. Rgs1, a regulator of G-protein signaling, interacts with all the three Gα subunits and functions as a negative regulator of G-proteins in *M. oryzae* [52]. Additionally, both *MGB1* and *MGG1* are essential for appressorium formation and plant infection [53], [54]. *M. oryzae PTH11* which encodes a putative G-protein-coupled receptor may be involved in regulating Mac1 activities, because *PTH11* is required for surface recognition and virulence and exogenous cAMP restores appressorium formation and pathogenicity in *PTH11* deletion mutants [55]. Recently, we reported that MoRic8 interacts with MagB and acts upstream of the cAMP/PKA pathway to regulate multiple stages of infection-related morphogenesis in *M. oryzae* [56]. However, downstream targets of the cAMP/PKA pathway are not well studied in *M. oryzae*.

Here, we present the identification and functional characterization of two novel pathogenicity-related genes identified by insertional mutagenesis, *MoSOM1* and *MoCDTF1*, which are required for morphogenesis and virulence. Our results have provided evidence that *MoSOM1* and *MoCDTF1* are regulated by the cAMP/PKA pathway. Deletion of either *MoSOM1* or *MoCDTF1* resulted in defects in hyphal growth, sporulation, appressorium formation and virulence. MoSom1 strongly interacted with the transcription factors, MoCdtf1 and MoStu1, and also weakly interacted with CpkA in yeast two hybrid assays performed in the presence of cAMP. Moreover, *MoSOM1* can complement the defects of *S. cerevisiae flo8* in haploid invasive growth and diploid pseudohyphal development. When considered together, these data suggest that MoSom1 is an important regulator of infection-related development in *M. oryzae* which interacts with the transcription factors, MoCdtf1 and MoStu1, and acts downstream of the cAMP/PKA signaling pathway.

## Results

### Identification of the T-DNA-tagged genes *MoSOM1* and *MoCDTF1* of *M. oryzae*

**Figure 1:**
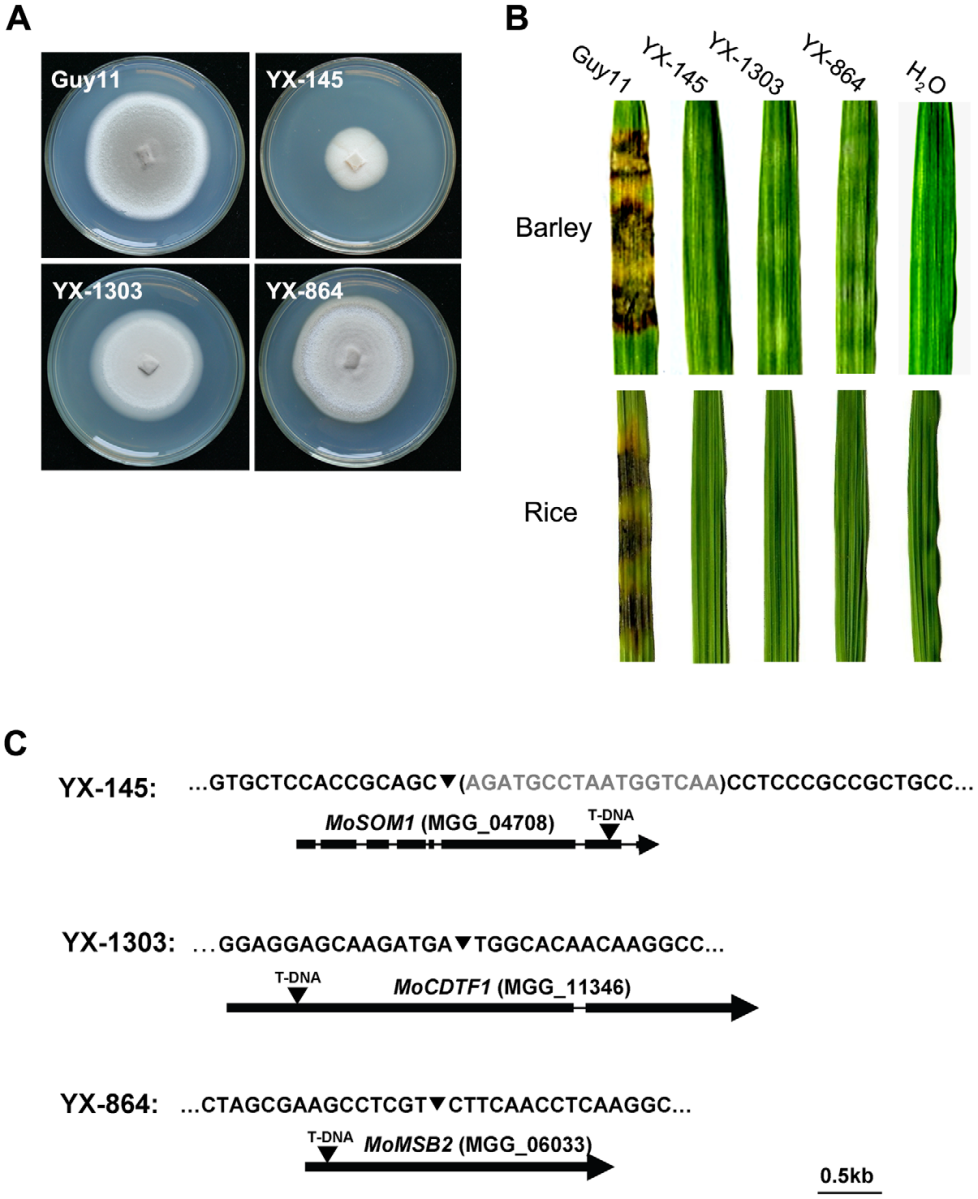
Identification of the T-DNA-tagged genes *MoSOM1* and *MoCDTF1*. (A) Colonies of the wild type strain Guy11 and three T-DNA insertional mutants. (B) Barley and rice leaf segments were inoculated with the mycelia from Guy11, YX-145, YX-1303 and YX-864, H_2_O was used as the control. (C) Position of the T-DNA insertion in YX-145, YX-1303 and YX-864 mutants. The nucleotide in the brackets was deleted by T-DNA integration. The arrows (▾) indicate the T-DNA (2.2 kb) insertion positions in *MoSOM1*, *MoCDTF1* and *MoMSB2* genes, respectively. The thick boxes represent the coding regions and the thin line joining these coding regions indicates the position of the introns.

To investigate the molecular basis of plant infection by *M. oryzae*, a large T-DNA insertional mutagenesis library (∼20,000 transformants) was constructed. All of the transformants were first screened for impairment in pathogenesis by inoculating barley leaves (cv. Golden Promise) with conidia or hyphae (if conidia were not available) using a barley cut-leaf assay. The mutants obtained from the first round screening were subsequently verified by inoculating rice leaves. Among them, YX-145, YX-1303 and YX-864 (Figure 1A; Table S1) were identified as mutants, which were incapable of causing disease on barley or rice leaves (CO-39) following inoculation with hyphae (Figure 1B). To identify the T-DNA integration sites in the mutants, genomic DNA flanking the integrated T-DNAs was obtained from the third round PCR products (Figure S1) and sequenced, respectively. By amplifying the genomic DNAs flanking the left border of the integrated T-DNA, the patterns of T-DNA integrated into these mutants were determined (Figure 1C).

The T-DNA insertion in YX-145 was found at position 593835+, which is 2457 bp downstream of the translational start site, in the seventh exon of a hypothetical gene MGG_04708 (GenBank XP_362263) located on supercontig 16 of chromosome IV. We named the T-DNA tagged gene *MoSOM1*, because it putatively encodes a predicted protein which is homologous with Som1 proteins, which may be involved in the cAMP-dependent protein kinase pathway controlling growth polarity in related fungal species. MoSom1 showed 47.54, 36.66, 36.84, 37.29, 51.47 and 47.96% amino acid identity with *Neurospora crassa* Som1 (AAF75278), *Aspergillus nidulans* OefA (AAW55626), *A. niger* Som1 (XP_001395127), *A. fumigatus* Som1 (XP_746706), *Metarhizium acridum* Som1 (EFY91592) and *Verticillium albo-atrum* Som1 (XP_003006356), respectively. However, MoSom1 showed only 14.76% and 14.93% amino acid identity with *Saccharomyces cerevisiae* Flo8 (DAA07769) and *Candida albicans* Flo8 (AAQ03244 ), respectively. Phylogenetic analysis of the putative homologs of MoSom1 was shown in Figure S2A.

The T-DNA integration site in YX-1303 was at position 1126131-, which is 544 bp downstream of the translational start site, in the first exon of a hypothetical gene MGG_11346 (GenBank XP_001413674) located on supercontig 27 of chromosome I. The T-DNA tagged gene putatively encodes a protein with no known function. We named the gene *MoCDTF1* (for *Magnaporthe oryzae* cAMP-dependent transcription factor gene). MoCdtf1 showed 21.44, 24.23, 18.73 and 27.37% amino acid identity with *N. crassa* NCU00124 (XP_957248), *Sclerotinia sclerotiorum* SS1G_07310 (XP_001591864), *A. nidulans* AN4210 (XP_661814) and *Gibberella zeae* FG06653 (XP_386829). However, no homolog of MoCdtf1 exists in the genomes of the yeasts *Saccharomyces cerevisiae* and *C. albicans*. Phylogenetic analysis of the putative homologs of MoCdtf1 was shown in Figure S2B.

In the YX-864 mutant, *MoMSB2* (MGG_06033) was disrupted by T-DNA integration (Figure 1C). To verify the non-pathogenic phenotype of YX-864, we performed a targeted gene deletion of *MoMSB2* (Figure S3A). The resulting *Δmomsb2* null mutants, MK9 and MK12 (Table S1), were selected by Southern blot analysis (Figure S3B); and were also confirmed by the lack of *MoMSB2* transcript using RT-PCR amplification with 864Q-F and 864Q-R (Table S2). Deletion of *MoMSB2* had no obvious effect on vegetative growth, conidial germination and sexual development, but caused defects in conidiation, appressorium formation and virulence (Figure S4). The defect in appressorium formation could not be restored by adding exogenous 1,16-hexadecanediol (Diol), cyclic adenosine 3′,5′-cyclophosphate (cAMP), and 3-iso-butyl-1-methylxanthine (IBMX). In *S. cerevisae*, it has been shown that Msb2 interacts with Sho1 and Cdc42 to promote their function in the filamentous growth pathway [57]. However, no direct interactions between MoMsb2 and MoSho1 (MGG_09125) and MoCdc42 (MGG_00466) were detected in yeast two hybrid assays (data not shown). Taken together, our data provide evidence that *MoMSB2* is required for plant infection-related morphogenesis and virulence in *M. oryzae*, which is consistent with a very recent study in which the gene was independently identified [49].

### Both *Δmosom1* and *Δmocdtf1* mutants are non-pathogenic

To determine the role of *MoSOM1* in plant infection and confirm the predicted role based on phenotypic analysis of YX-145, we performed targeted gene deletion of *MoSOM1* using the gene replacement vectors pMoSOM1-KO (Figure S3C). The gene replacement was analyzed by PCR amplification with primers 145-F and 145-R (Table S2) from transformants. The resulting *Δmosom1* null mutants, SK5, SK21 and SK27 (Table S1), were selected based on Southern blot analysis (Figure S3D) and also confirmed by RT-PCR amplification using primers 145Q-F and 145Q-R. One of the transformants resulting from ectopically integrated pMoSOM1-KO, ES16, was used as a control strain. To complement the mutant, the 2.8 kb *MoSOM1* gene-coding sequence and a 1.5 kb promoter region was re-introduced into SK27 (*Δmosom1*) to obtain two complemented strains, SC1 and SC3 (Table S1). Similarly, the *Δmocdtf1* null mutants, CTK2 and CTK15, were generated by a targeted gene deletion of *MoCDTF1* (Figure S3E and F). The complemented strains, CTC1 and CTC5, were obtained by transforming the genomic DNA including 4.1 kb *MoCDTF1* gene-coding sequence and a 1.6 kb promoter region back to *Δmocdtf1* (CTK15).

**Figure 2:**
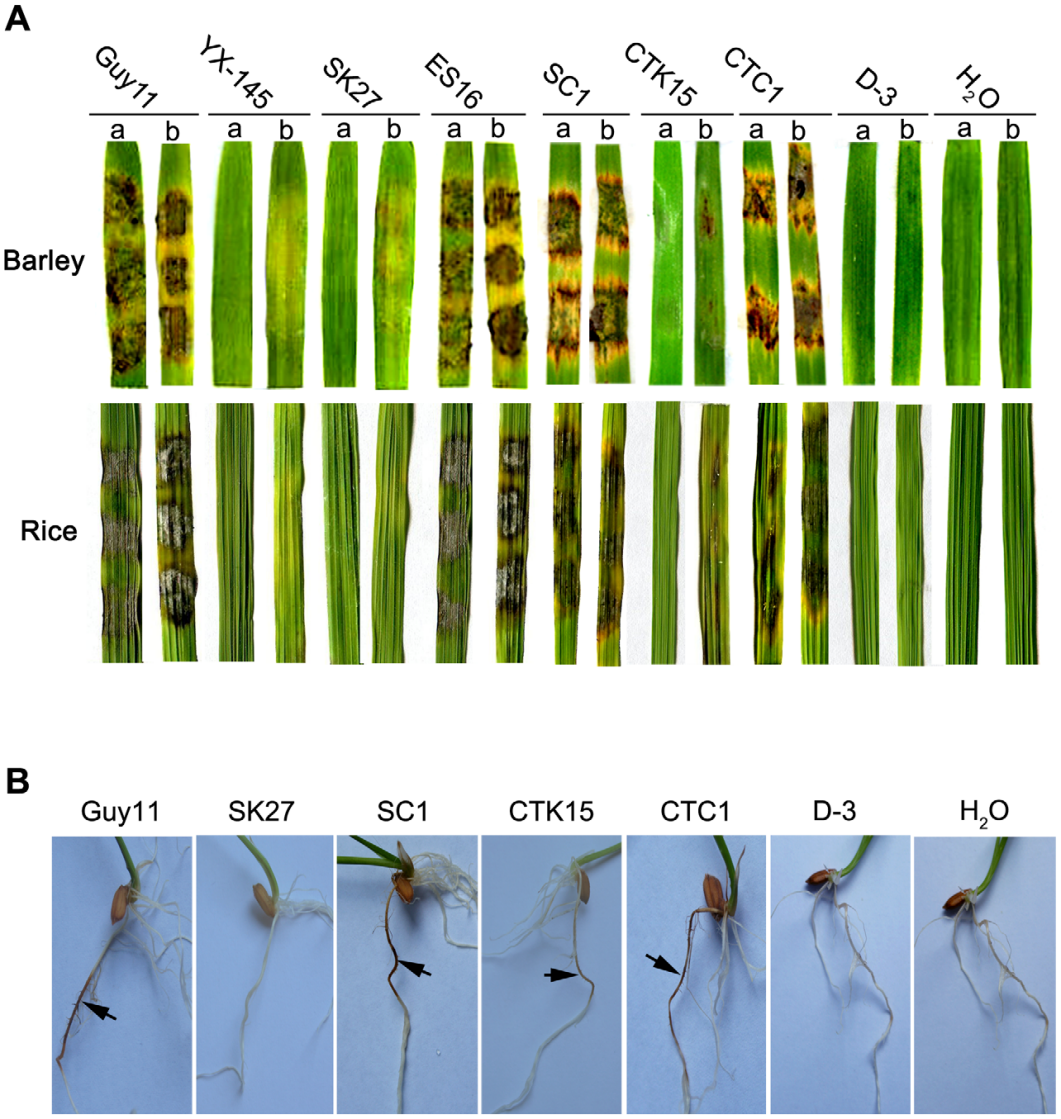
The *Δmosom1*, *Δmocdtf1* and *Δmosom1Δmocdtf1* mutants are nonpathogenic. (A) Barley and rice segments inoculated with the mycelia from the wild-type strain Guy11, YX-145, SK27 (*Δmosom1*), ES16 (ectopic transformant), SC1 (*Δmosom1*+*MoSOM1*), CTK15 (*Δmocdtf1*), CTC1 (*Δmocdtf1*+*MoCDTF1*) and D-3 (*Δmosom1Δmocdtf1*). a = unwounded leaf and b = abraded leaf. (B) Root infection assays. The mutants SK27 and D-3 were non-pathogenic when inoculated onto rice roots, but the mutant CTK15 was still able to cause some disease symptom. Arrows indicate necrotic lesions. H_2_O was used as the control. Photographs were taken at 5 days after inoculation.

We then harvested the mycelium of *Δmosom1* and *Δmocdtf1* mutants from liquid CM cultures to inoculate susceptible barley and rice using the cut leaf assay. Our results showed that the wild-type strain Guy11, ectopic (ES16) or complementation (SC1 and CTC1) transformants caused typical rice blast lesions on both intact and abraded barley or rice leaves (Figure 2A). However, consistent with the original analysis of YX-145, the *Δmosom1* (SK27) mutant was non-pathogenic on both susceptible barley and rice leaves, even when they were abraded to remove the surface cuticle (Figure 2A). The *Δmocdtf1* (CTK15) mutant was non-pathogenic on both barley and rice leaves, but was still able to cause some disease symptoms when leaf surfaces were abraded (Figure 2A). We were unable to carry out a pathogenicity assay using spray inoculation, because these mutants were completely defective in sporulation in culture (see below). Furthermore, the *Δmosom1* (SK27) mutant was non-pathogenic when inoculated onto rice roots, but the *Δmocdtf1* (CTK15) mutant was still able to cause some disease symptom (Figure 2B). These results therefore demonstrated that the non-pathogenic phenotype of YX-145 and YX-1303 mutants was caused by T-DNA integration and that both *MoSOM1* and *MoCDTF1* are crucial for plant infection in *M. oryzae*.

### *MoSOM1* and *MoCDTF1* are important in vegetative growth and colony pigmentation

**Figure 3 ppat-1002385-g003:**
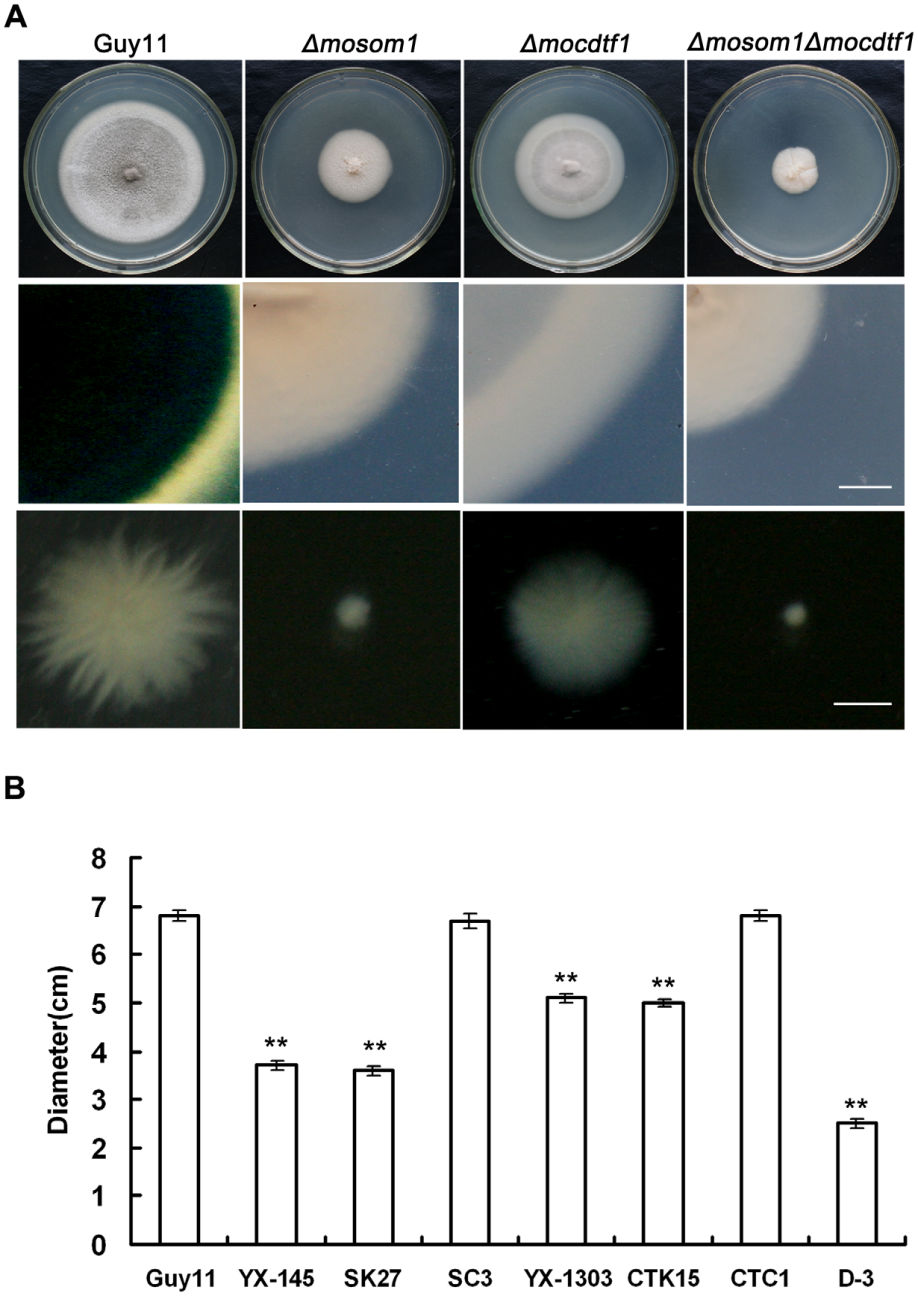
*MoSOM1* and *MoCDTF1* are required for vegetative growth and pigmentation. (A) Mycelium growth and pigmentation were significantly impaired in SK27 (*Δmosom1*), CTK15 (*Δmocdtf1*) and D-3 (*Δmosom1Δmocdtf1*) mutants. The mutants and the wild type strain (Guy11) were inoculated on CM medium and cultured at 25°C for 10 days (top). Reduced pigmentation was observed from the colony back side of the mutants (middle). Mycelium growth patterns of the strains in liquid CM medium at 25°C for 48 h (bottom). Scale bars = 5 mm. (B) Bar chart showing the colony diameters of Guy11, YX-145, SK27, ES16 (ectopic transformant), SC3 (*Δmosom1*+*MoSOM1*), YX-1303, CTK15, CTC1 (*Δmocdtf1*+*MoCDTF1*) and D-3. Error bars represent standard deviation. Asterisks indicate significant difference at *P* = 0.01.

Deletion of *MoSOM1* caused significant defects in hyphal growth and colony pigmentation (Figure 3A). The *Δmosom1* mutant formed colonies that were less pigmented and which formed less aerial hyphae (Figure 3A). All *Δmosom1* mutants (SK5, SK21 and SK27) showed the same phenotypes and only data for mutant SK27 are therefore presented here. When the *Δmosom1* mutant (SK27) was grown in CM liquid culture, it formed very small compact mycelium masses, in contrast to the bigger but less compact mycelium formed by the wild-type strain (Figure 3A). The growth rate of mycelium from each strain was determined (Figure 3B). The *Δmosom1* mutant and YX-145 were significantly reduced in vegetative growth, forming colonies with diameters of 3.6±0.09 cm and 3.7±0.08 cm after 10-day incubation on CM at 25°C, respectively, compared with 6.8±0.1 cm colony diameter of wild-type strain Guy11 (*P*<0.01) (Figure 3B). We also carried out mycelial dry weight assays. The results showed that the *Δmosom1* mutant was significantly reduced in mycelial dry weight with 0.151±0.007 g compared with 0.330±0.015 g of the wild-type strain Guy11 (*P*<0.01) after 2-day incubation in liquid CM at 25°C.

Deletion of *MoCDTF1* also caused defects in vegetative growth and colony pigmentation on CM plate cultures compared with the wild-type strain, although the affected degree was not as severe as in *Δmosom1* mutants (Figure 3A). The *Δmocdtf1* mutant (CTK15) formed mycelium that was not well pigmented compared with the wild-type strain and formed smaller mycelium masses in liquid culture (Figure 3A). The *Δmocdtf1* mutant and YX-1303 were reduced in vegetative growth, forming colonies with diameters of 5.0±0.08 cm and 5.1±0.1 cm after 10-day incubation on CM at 25°C, respectively, compared with 6.8±0.1 cm colony diameter of wild-type strain Guy11 (*P*<0.01) (Figure 3B). The other *Δmocdtf1* mutant (CTK2) had the same phenotypes as CTK15 (data not shown). To further investigate the roles of *MoSOM1* and *MoCDTF1*, two *Δmosom1Δmocdtf1* mutants D-3 and D-9 were created by transformation of pMoSOM1-DK (Figure S3G) into the strain CTK15 (*Δmocdtf1*) and selected by PCR and confirmed by RT-PCR with the primers 145-F and 145-R (Figure S3H and I), respectively. The *Δmosom1Δmocdtf1* mutant D-3 grew more slowly than both the *Δmosom1* (SK27) and *Δmocdtf1* mutants (CTK15) in culture (Figure 3A and B). Additionally, when the *Δmosom1* mutant (SK27) and *Δmocdtf1* mutant (CTK15) were inoculated on various media, including MM, PDA and OMA, their vegetative growth and colony pigmented were also impaired (Figure S5). We conclude that *MoSOM1* and *MoCDTF1* are required for vegetative growth and mycelium pigmentation.

### Deletion of either *MoSOM1* or *MoCDTF1* completely blocks production of asexual and sexual spores

**Figure 4:**
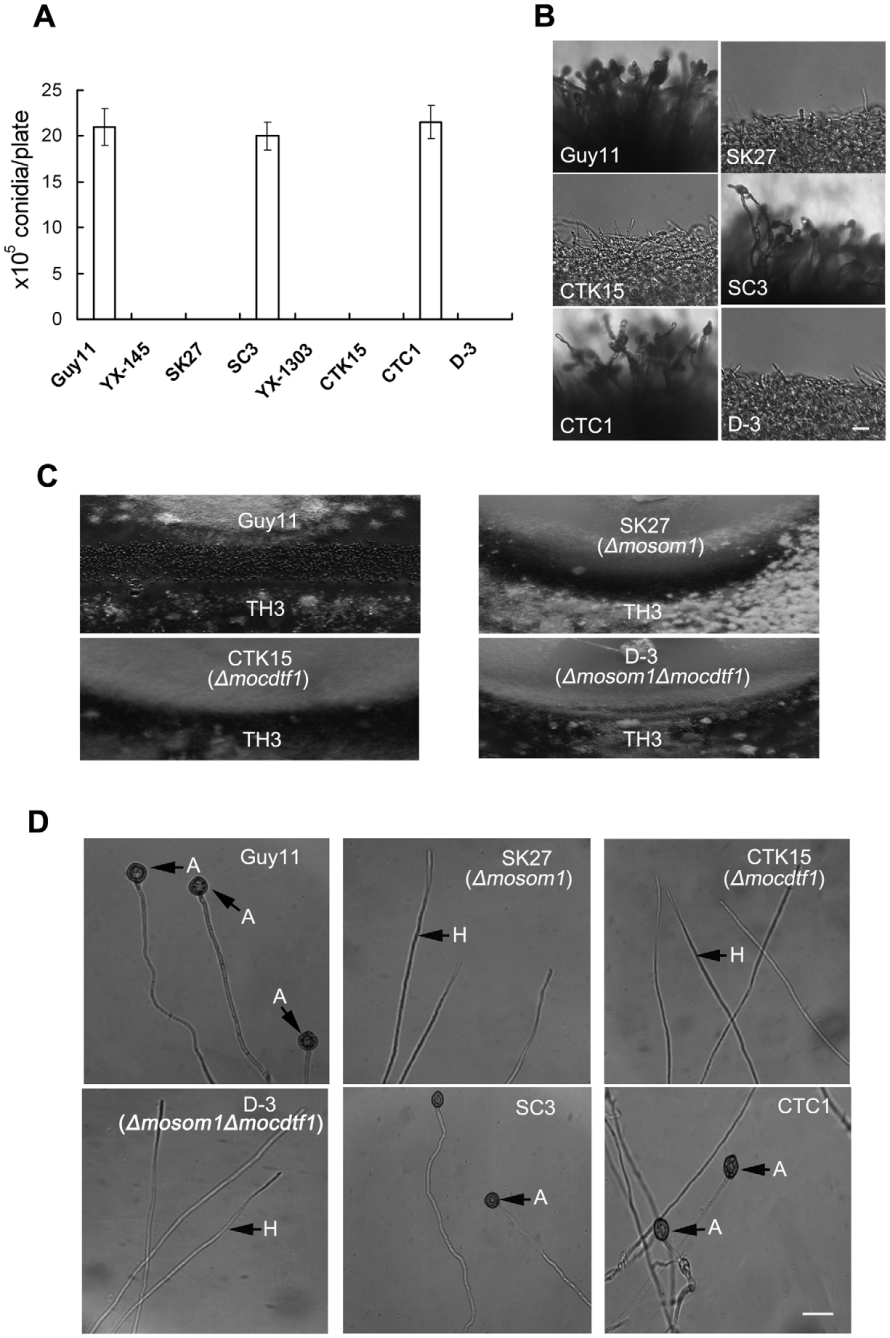
*MoSOM1* and *MoCDTF1* are essential for producing asexual/sexual spores and appressoria. (A) Bar chart showing the conidial production. The YX-145, SK27 (*Δmosom1*), YX-1303, CTK15 (*Δmocdtf1*) and D-3 (*Δmosom1Δmocdtf1*) mutants were unable to produce any conidia, while the wild type strain Guy11, SC3 (*Δmosom1*+*MoSOM1*) and CTC1 (*Δmocdtf1*+*MoCDTF1*) formed numerous conidia on CM medium at 25°C for 10 days. Error bars represent standard deviation. (B) Microscopic observation of conidial development. Aerial hyphae were significantly reduced and conidiophores were not observed in the SK27, CTK15 and D-3 mutants. The wild-type strain Guy11 and the complementation strains (SC3 and CTC1) formed normal conidiophores and numerous conidia. All the tested strains grown on CM medium for 4 days were examined by light microscopy and photographed. Scale bar = 20 µm. (C) Fertility assay. Guy11×TH3 formed numerous perithecia on oatmeal medium after 3-week incubation in an inductive condition, while no perithecia was observed for the crosses of SK27×TH3, CTK15×TH3 and D-3×TH3. (D) Appressorium formation assay. Mycelium fragments of Guy11, SK27, CTK15, D-3, SC3 and CTC1 were placed on hydrophobic GelBond film surfaces to allow appressorium development, respectively. Appressorium formation was observed after 24 h incubation at 25°C in darkness. Numerous appressoria were produced by Guy11 and the complementation strains (SC3 and CTC1), however, no appressoria were observed for the inoculation of SK27, CTK15 and D-3. A = appressorium; H = hypha. Scale bar = 10 µm.

The ability to form spores was evaluated by carefully washing the surface of 10-day-old cultures on CM plates. YX-145, SK27, YX-1303 and CTK15 were unable to form conidia, while the wild-type strain Guy11 produced numerous conidia with 21.0±2.0×10^6^ spores per plate (Figure 4A). When these mutants were grown on different growth media, including MM, PDA, OMA, sporulation was also not observed. These results showed that asexual sporulation was completely blocked by the deletion/disruption of either *MoSOM1* or *MoCDTF1*, indicating that each of the two genes is essential for conidiation in *M. oryzae*. Furthermore, no conidiophores were observed from the cultures of the mutants, while Guy11 formed normal conidiophores and conidia (Figure 4B). The phenotypes were also observed from other targeted gene replacement mutants, such as SK5, SK21 and CTK2. These results suggest that the defect in conidiation of the *Δmosom1* and *Δmocdtf1* mutants may be caused by the lack of aerial conidiophore development.

To determine the role of *MoSOM1* and *MoCDTF1* in sexual reproduction, the wild type Guy11 (*MAT1-2*), SK27 and CTK15 were crossed with a standard tester strain TH3 (*MAT1-1*) of *M. oryzae* to allow perithecium production. After three weeks, the junctions between mated individuals were examined for the presence of perithecia. We observed numerous perithecia at the junctions of the wild type strains Guy11 and TH3, but no perithecia were formed after crossing SK27 with TH3 or CTK15 with TH3 (Figure 4C), even when the incubation time was extended to six weeks. Similarly, crossing of TH3 with the T-DNA insertional mutants (YX-145 and YX-1303), SK5, SK21 and CTK2 did not produce any perithecia, indicating that *MoSOM1* or *MoCDTF1* are essential for fertility and development of fruiting bodies by *M. oryzae*. The *Δmosom1Δmocdtf1* mutant D-3 was also unable to produce conidiophores, conidia and was completely impaired in sexually development (Figure 4A–C). We conclude that *MoSOM1* and *MoCDTF1* are both essential for production of asexual and sexual spores by *M. oryzae*.

### Both *MoSOM1* and *MoCDTF1* are required for appressorium formation from mycelium

Since the *Δmosom1* and *Δmocdtf1* mutants were unable to produce spores, we harvested mycelium of the mutants from liquid CM culture and appressorium formation was investigated by placing hyphae on hydrophobic surfaces. Numerous appressoria were formed from mycelium of the isogenic wild type strain Guy11, but no appressoria were observed at 24 h or even 48 h post inoculation with the *Δmosom1* (SK27) and *Δmocdtf1* (CTK15) mutants (Figure 4D). When mycelium of these mutants was placed on barley or rice leaf surfaces, no appressorium formation was induced and no penetration events were observed at 24 h post inoculation (data not shown), indicating the non-pathogenic phenotypes of *Δmosom1* and *Δmocdtf1* mutants on host leaves may be caused by the defect in appressorium formation. The *Δmosom1Δmocdtf1* was also unable to form appressoria from mycelium (Figure 4D). These results suggest that *MoSOM1* and *MoCDTF1* are both required for appressorium formation and plant infection by *M. oryzae*.

### Both *Δmosom1* and *Δmocdtf1* mutants show an easily wettable phenotype

**Figure 5:**
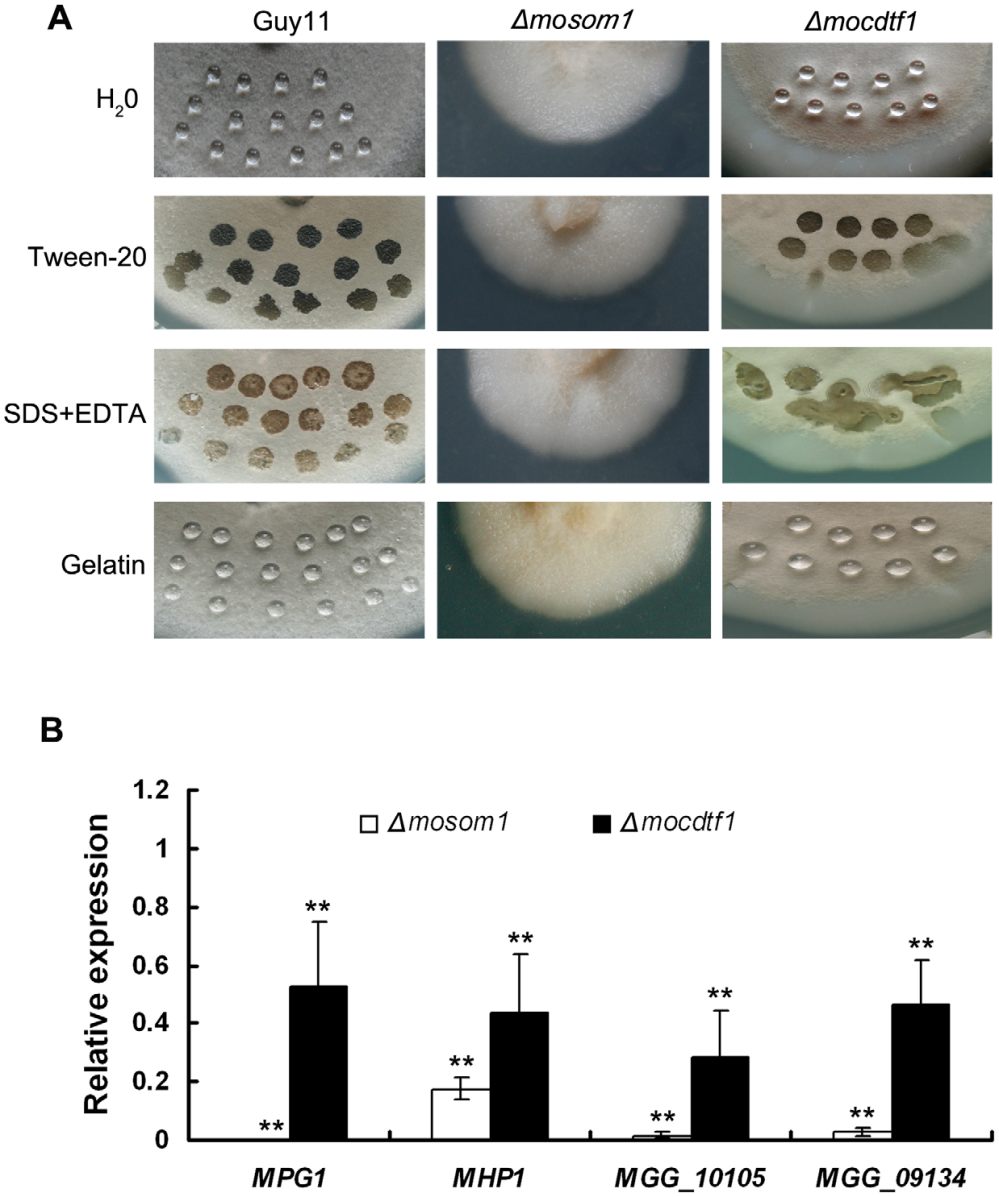
Both *Δmosom1* and *Δmocdtf1* mutants show an easily wettable phenotype. (A) Surface hydrophobicity of the wild type strain Guy11 and the mutants SK27 (*Δmosom1*) and CTK15 (*Δmocdtf1*) was assessed by placing a 10 µl drop water, 250 µg/ml tween20, 0.2% SDS+50 mM EDTA, and 0.2% gelatin on the 7-day-old cultures, respectively. Drops of water and 0.2% gelatin remained on the cultures of Guy11 and the old mycelium of the mutant CTK15, while the others were soaked into colonies. The photographs were taken after 12 h incubation. (B) Expression of the genes coding hydrophobins in the *Δmosom1* (SK27) and *Δmocdtf1* (CTK15) mutants measured by quantitative reverse-transcription polymerase chain reaction (qRT-PCR). Error bars represent the standard deviation. Asterisks indicate significant difference at *P* = 0.01.

An easily wettable phenotype can be observed when a fungal culture becomes easily water-logged, due to a loss of surface hydrophobicity, brought about by the absence of the rodlet layer associated with aerial hyphae and conidiospores [58]. We observed that colonies of YX-145 and *Δmosom1* mutants were distinct from the wild-type strain Guy11 and formed less aerial hyphae. YX-1303 and *Δmocdtf1* mutants were also reduced in aerial hypha formation. We therefore tested the surface hydrophobicity of these strains (Figure 5A). Drops of water and 0.2% gelatin remained on the surface of mycelium of Guy11 and older mycelium of the *Δmocdtf1* mutant (CTK15) after 24–48 h incubation, and drops of detergent solution remained suspended on the surface of colonies of Guy11 for about 10–30 min before soaking into the mycelium. By contrast, drops of water and detergent solution immediately soaked into the cultures of the *Δmosom1* mutant (SK27) and young mycelium of CTK15 (Figure 5A). Similar results were observed for the other *Δmosom1* and *Δmocdtf1* mutants. The surface hydrophobicity of the double knockout mutants D-3 and D-9 was similar to the *Δmosom1* mutants. The results indicate that deletion of either *MoSOM1* or *MoCDTF1* affects cell surface hydrophobicity in *M. oryzae*.

As a consequence of the wettable phenotype of the mutants, we reasoned that *M. oryzae* hydrophobin genes might be down-regulated in the mutants. To test this idea, we investigated the expression of *M. oryzae* hydrophobin-encoding genes, including *MPG1* and *MHP1* and two *MHP1* homologs (MGG_09134 and MGG_10105), by quantitative RT-PCR (qRT-PCR). We found that expression of hydrophobin encoding genes was significantly (*P<0.01*) down-regulated in both *Δmosom1* and *Δmocdtf1* mutants, particularly in the *Δmosom1* mutant (Figure 5B).

### Both MoSom1 and MoCdtf1 are localized to the nucleus

**Figure 6:**
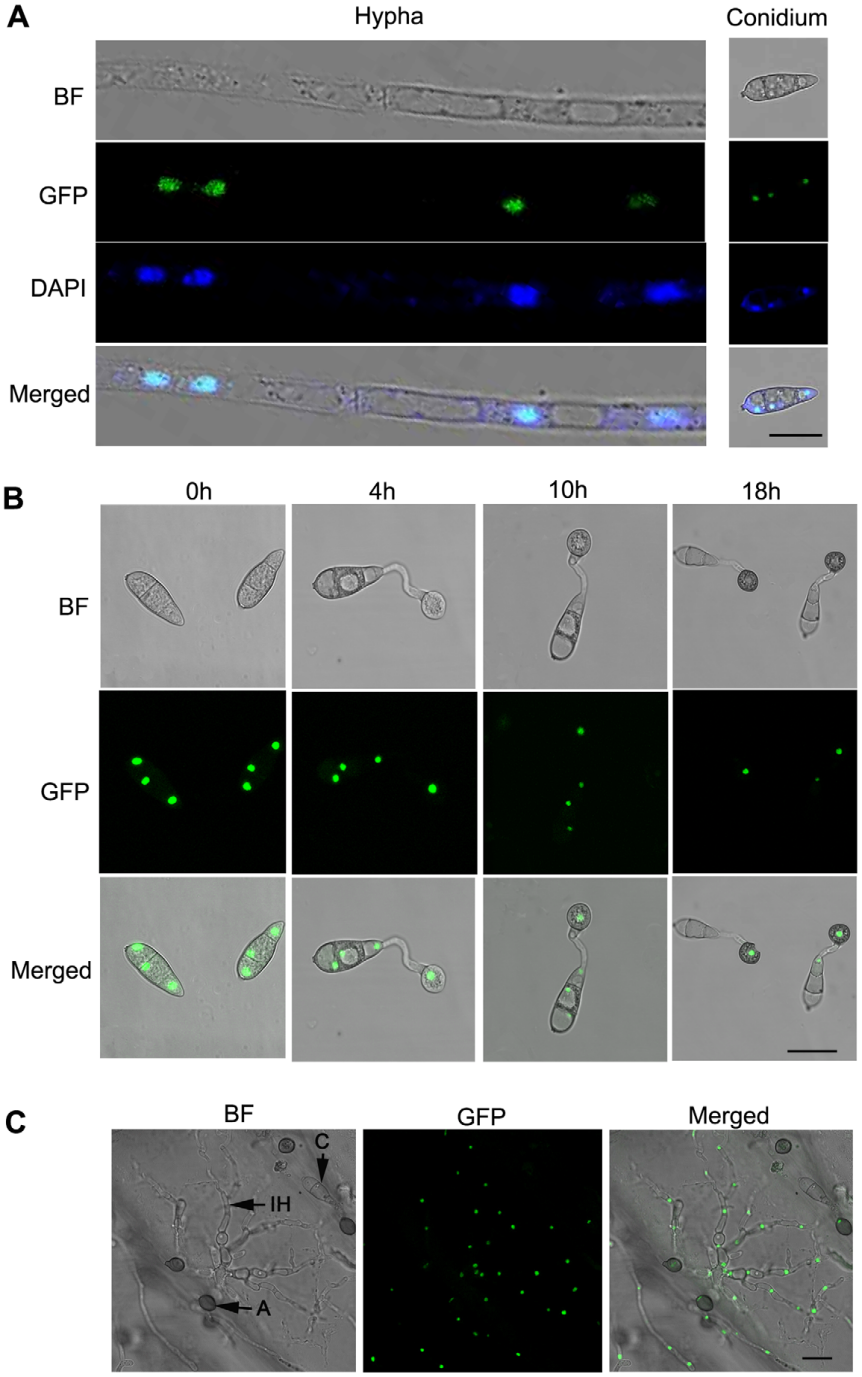
Intracellular localization of MoSom1-green fluorescent protein. (A) MoSom1 was localized to the nucleus. MoSom1 C-terminal green fluorescent protein (GFP) fusion strategy was conducted to generate GFP expression transformants. One of the transformants, SC3, was used for MoSom1 localization assay. Conidia and mycelia of SC3 were stained by DAPI (4′-6-Diamidino-2-phenylinodle). The merged image of GFP and DAPI staining showed that MoSom1-GFP localized in the nucleus. (B) The patterns of *MoSOM1* expression and nuclear division during appressorium development. Conidia of the strain SC3 was allowed to germinate on hydrophobic GelBond film surfaces. Photographs were taken at various time intervals. (C) Expression of *MoSOM1* during invasive growth. The assay was performed by placing 30 µl conidial suspension of SC3 on onion epidermis. Photographs were taken at 48 h after incubation. Arrows indicate conidium, appressorium or invasive hypha inside cells. C = conidium; A = appressorium; IH = invasive hypha. BF = bright field. All of bars = 10 µm.

To investigate the expression pattern of *MoSOM1* during infection-related development, a 1.52 kb promoter fragment upstream of the gene and the entire MoSom1 protein-coding sequence were fused in-frame to the green fluorescent protein (GFP)-encoding gene, *GFP* (*sGFP*), and introduced into the *Δmosom1* mutant SK27. Transformants carrying a single integration of the pMoSOM1-GFP were selected by DNA gel blot analysis. An independent single plasmid insertion event occurred in the transformants, SC1 and SC3 (Table S1). Punctate green fluorescence was observed in the two transformants. SC3 was used to investigate the spatial localization of the MoSom1 protein in detail. In this analysis, GFP fluorescence was observed both in mycelium and in conidia of SC3, and each cell contained one fluorescence punctum (Figure 6A), suggesting that MoSom1 may localize to the nucleus of each cell. To test this idea, mycelium and conidia of SC3 were stained with 4′-6-Diamidino-2-phenylinodle (DAPI) to stain nuclei specifically. The merged image of GFP and DAPI staining showed that MoSom1-GFP localizes to the nucleus and that each cell contains a single nucleus (Figure 6A). To observe *MoSOM1* expression and nuclear division patterns during appressorium development in *M. oryzae*, conidia of the strain SC3 were allowed to germinate on hydrophobic GelBond film surfaces. During conidium germination, the nucleus in the germinating cell entered mitosis and then one of the daughter nuclei migrated to the incipient appressorium (Figure 6B). Three nuclei that remained in the conidium degenerated and could no longer be seen after approximately 18 hours post inoculation, consistent with previous observations of nuclear division in *M. oryzae* [59]. Bright green fluorescence of the strain SC3 during penetration on onion epidermis was also observed, as shown in Figure 6C. However, qRT-PCR analysis showed that the expression levels of *MoSOM1* were similar at different developmental stages (data not shown), indicating expression throughout the life cycle of the fungus. The expression and localization of MoSom1-GFP was identical in the other transformant SC1. A similar strategy was used to investigate the expression pattern of *MoCDTF1* and localization of the encoded protein during infection-related development. Green fluorescence was also observed in nuclei, both in mycelium and in conidia of the transformants CTC1 (Figure S6) and CTC5. However, weak GFP fluorescence was observed in mycelium and in conidia of CTC1 and CTC5 compared strong GFP fluorescence observed in SC1 and SC3. These results provide evidence that both MoSom1 and MoCdtf1 proteins are localized to the nucleus in *M. oryzae*.

### Complementation analysis of *Δmosom1* and *Δmocdtf1* mutants

To ensure that all phenotypes observed in the *Δmosom1* and *Δmocdtf1* mutants were associated with the gene replacement event, we carried out phenotypic analysis of complemented transformants SC1, SC3, CTC1 and CTC5. The GFP-expressing transformants SC1 and CTC1 exhibited full virulence to barley and rice by cut-leaf assay using mycelium inoculations (Figure 2A) or by seedling assays with conidial spray-inoculation. The other phenotypes of *Δmosom1* and *Δmocdtf1* mutants, including vegetative growth, conidiation and appressorium formation, were all fully complemented by re-introduction of the genes of *MoSOM1* or *MoCDTF1* (Figure 3B; Figure 4A, B and D; Figure S7). However, the mutants were not responsive to 10 mM exogenous cAMP (data not shown), indicating MoSom1 and MoCdtf1 may act downstream of the cAMP/PKA pathway. We conclude that *MoSOM1* or *MoCDTF1* are both essential for multiple steps of plant infection-related morphogenesis development and pathogenicity in *M. oryzae*.

### The MoSom1 protein has different spliced isoforms in *M. oryzae*

To confirm the position and size of the introns of *MoSOM1* and *MoCDTF1*, cDNA clones of the coding sequence were obtained by reverse transcription-PCR with primer pairs of SOM-E-F/SOM-Xh-R and P1303-F/1303H-Kpn-R (Table S2) and the resulting PCR products cloned into pGEM-T easy vectors and sequenced, respectively. Comparison of the cDNA and sequenced genomic DNA confirmed that *MoCDTF1* has an open reading frame of 4,121 bp interrupted by one intron (62 bp) and putatively encodes a 1352 aa protein, which is identical to the protein sequence predicted by automated annotation of the *M. oryzae* genome sequence (ID: MGG_11346.6; Broad Institute). *MoSOM1* has an open reading frame of 2,789 bp interrupted by seven introns (56 bp, 85 bp, 62 bp, 24 bp, 66 bp, 81 bp and 68 bp, respectively) and putatively encodes a 781 aa protein (ID: MGG_04708.6; Broad Institute). However, five splice variants of MoSom1 were also found, as shown in Figure S8. Furthermore, all of the alternatively spliced isoforms of MoSom1 could be detected in RNA extracted from mycelium cultured in liquid CM (1 d, 3 d and 5 d) or conidia from 10-day-old CM plates (data not shown). Three missed amino-acid fragments occurred in exons 4, 6 and 7, respectively, while the extra amino-acid fragment was in exon 4. These data suggested that there may be various forms of post-transcriptional modification of *MoSOM1* in *M. oryzae*.

### Functional characterization of different domains of MoSom1 and MoCdtf1

**Figure 7:**
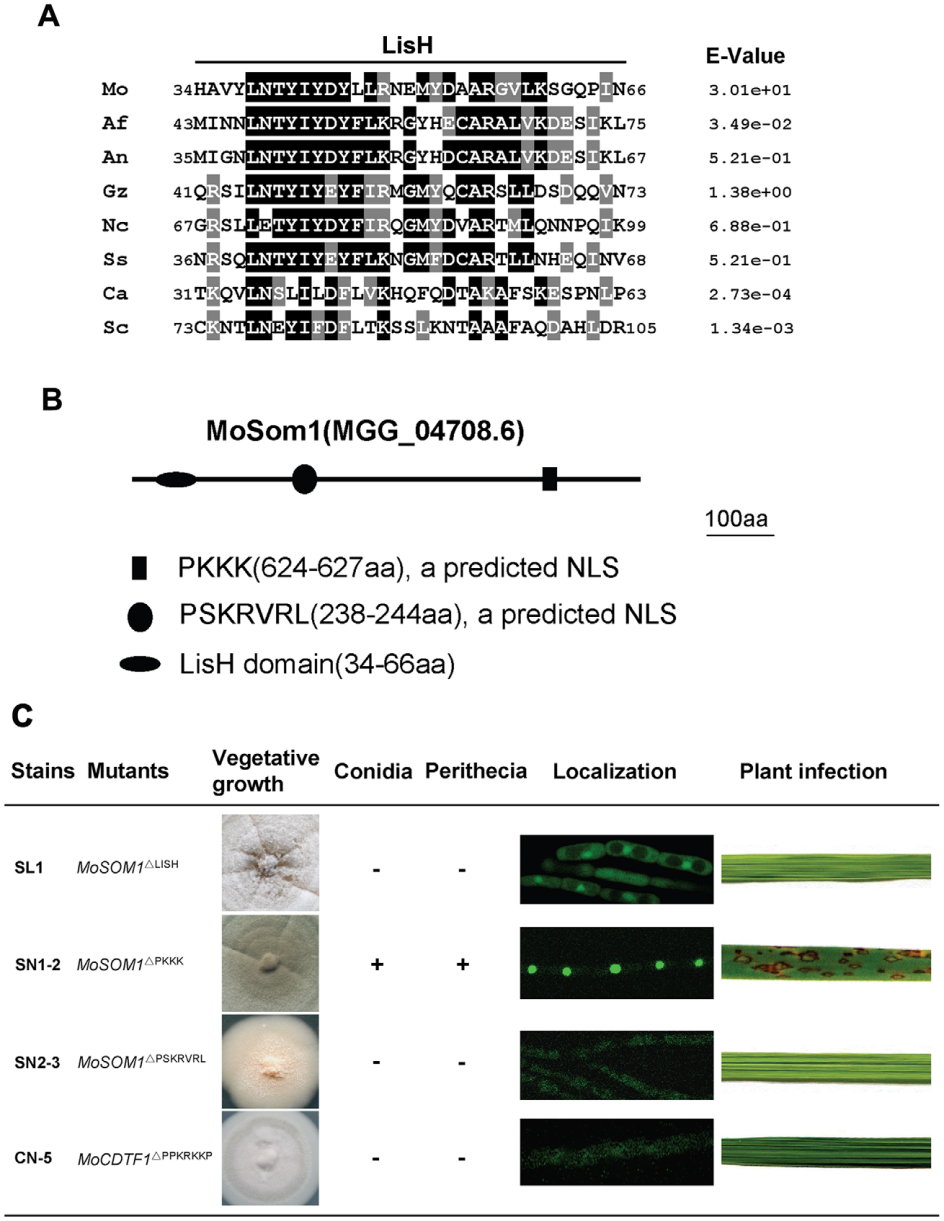
Functional analysis of putative LisH domain and nuclear localization sequences of MoSom1. (A) Sequence alignment of the LisH domain between *Magnaporthe oryzae* MoSom1 and other regulatory proteins. Identical residues are shaded in black and conserved residues are shaded in gray. Abbreviations correspond to species names. The fungal species, proteins and GenBank accession numbers are: Mo, *M. oryzae* MoSom1_XP_362263; Af, *Aspergillus fumigatus* putative Som1_XP_746706; An, *A. nidulans* putative OefA_AAW55626; Gz, *Gibberella zeae* putative Som1_XP_382826; Nc, *Neurospora crassa* putative Som1_AAF75278; Ss, *Sclerotinia sclerotiorum* putative Som1_XP_001598877; Ca, *Candida albicans* Flo8_AAQ03244; Sc, *Saccharomyces cerevisiae* Flo8_DAA07769. (B) Position of LisH domain and two predicted nuclear localization sequences (NLSs) in MoSom1 protein. (C) Functional analysis of domains. Like *Δmosom1* mutants, deletion of LisH domain of *MoSOM1* resulted in pleiotropic defects. The predicted NLS of PSKRVRL but not PKKK was required for MoSom1 localization to the nucleus and its function. The predicted NLS (PPKRKKP) was also crucial for the function and localization of MoCdtf1 protein. +, normal asexual or sexual sporulation; -, not any conidia or perithecia. The LisH domains were predicted at: http://smart.embl-heidelberg.de/; The NLSs were predicted at: http://psort.hgc.jp/form2.html.

Both *S. cerevisiae* Flo8 and *C. albicans* Flo8 contain a LUFS (LUG/LUH, Flo8, Single-stranded DNA binding protein) domain and there is a LisH (Lissencephaly type 1-like homology) motif within the domain. Similarly, a LUFS domain harbored a LisH motif was also found at the N-terminal portion of the *M. oryzae* MoSom1 protein (Figure S9A). The amino acid alignment of LisH domains of MoSom1 homologs from related fungal species were shown in Figure 7A, indicating that the fungal LisH domain in fungi is conserved. In addition, MoCdtf1 has a C-terminal ZnF_C2H2 domain. The amino acid alignment of the putative zinc finger, ZnF_C2H2 domain in MoCdtf1 was shown in Figure S9B.

The position of the LisH domain of MoSom1 was shown in Figure 7B. To explore the role of the LisH domain of the MoSom1 protein, we generated a mutant allele of *MoSOM1*-*GFP* by deletion of the LisH domain. The resulting transformants (SL1 and SL7) expressing *MoSOM1* ^ΔLISH^-GFP produced more aerial hyphae and formed more melanized colonies than the original *Δmosom1* mutant, but they were still defective in conidiation, asexual/sexual development and pathogenicity (Figure 7C). In these strains, the GFP fluorescence was observed both in nucleus and cytoplasm of hypha (Figure 7C), indicating that protein localization was somewhat affected by the deletion of LisH domain of *MoSOM1*. Additionally, mutants carrying deletions in the ZnF_C2H2 domain of MoCdtf1 had the same phenotypes as the original strain CTK15 (data not shown), indicating that the domain is essential for the function of MoCdtf1 in *M. oryzae*. These results indicated that both the LisH domain of MoSom1 and the ZnF_C2H2 domain of MoCdtf1 are essential for infection related morphorgenesis and virulence in *M. oryzae*.

Consistent with their observed localization patterns (Figure 6; Figure S6), both *M. oryzae* MoSom1 and MoCdtf1 were predicted to be nuclear localized proteins. The positions of two predicted nuclear localization signals (NLSs) were shown in Figure 7B. To determine the role of the predicted NLSs of MoSom1, we generated mutant alleles of *MoSOM1*-*GFP* deleted of each individual putative NLS (PKKK or PSKRVRL) and transformed them into the *Δmosom1* mutant (SK27). We found that transformants (SN1-2 and SN1-5) expressing the *MoSOM1* ^ΔPKKK^ -GFP grew normally on CM medium, produced numerous conidia and were fully pathogenic. Moreover, green fluorescence was still observed in the nucleus of these transformants (Figure 7C). However, like the original *Δmosom1* mutant, strains (SN2-3 and SN2-4) expressing the *MoSOM1* ^ΔPSKRVRL^-GFP were unable to produce asexual/sexual spores and were non-pathogenic. Interestingly, we observed green fluorescence of these strains in the cytoplasm of hypha (Figure 7C). These results suggest that PSKRVRL but not PKKK sequence is essential for the function and transportation of MoSom1 protein from cytoplasm to the nucleus. Using a similar strategy, we also demonstrated that the predicted NLS (PPKRKKP) of MoCdtf1 was crucial for the protein localized to the nucleus and its functions during differentiation and plant infection (Figure 7C).

### *MoSOM1* can complement *flo8* defects in haploid invasive growth of *S. cerevisiae* and diploid pseudohyphal development

**Figure 8:**
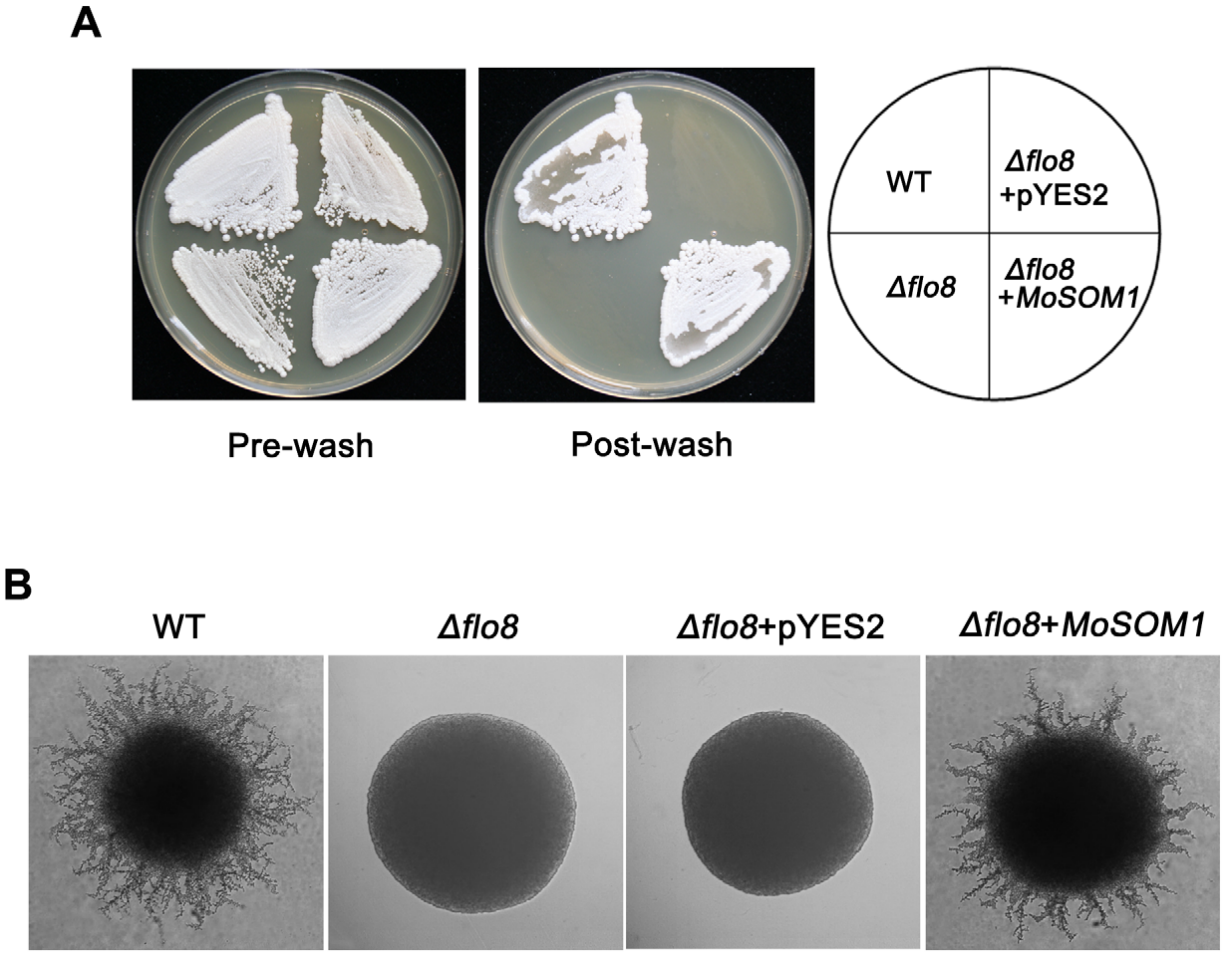
*MoSOM1* can complement *S. cerevisae flo8* defects in haploid invasive growth and diploid pseudohyphal development. (A) *MoSOM1* could complement *S. cerevisiae Δflo8* in invasive growth. The strains were grown on YPD at 30°C for 3 days. WT, Haploid wild-type (MY1384); *Δflo8*, HLY850; *Δflo8*+pYES2, HLY850 carrying a pYES2 vector; *Δflo8*+*MoSOM1*, HLY850 expressing *MoSOM1*. (B) *MoSOM1* could complement *S. cerevisiae Δflo8* in diploid pseudohyphal development. All strains were grown on SLAD at 30°C for 4 days except HLY852 was grown on SLAD with 200 mg/L URA (SIGMA). WT, diploid wild-type (CGx68); *Δflo8*, HLY852; *Δflo8*+pYES2, HLY852 carrying a pYES2 vector; *Δflo8*+*MoSOM1*, HLY852 expressing *MoSOM1*.

In *Saccharomyces cerevisae*, Flo8 is critical for invasive growth and flocculation in haploids and pseudohyphal growth in diploids [18]. To determine if *MoSOM1* can functionally complement the *S. cerevisae flo8* defects, we carried out yeast complementation assays. Our results showed that a yeast strain expressing *MoSOM1* in the haploid *flo8* mutant HLY850 was restored in its ability to carry out invasive growth on YPD medium (Figure 8A). Consistently, the strain expressing *MoSOM1* in the dipoliod *flo8* mutant HLY852 recovered the ability to carry out pseudohyphal development on SLAD (synthetic low ammonium dextrose medium) (Figure 8B). These data suggest that *MoSOM1* can functionally complement yeast *flo8* defects in both haploid invasive growth and diploid pseudohyphal development.

### *MoSOM1* and *MoCDTF1* are putatively regulated by the cAMP response pathway

To understand the regulation of *MoSOM1* and *MoCDTF1* by the cAMP/PKA pathway, the expression of both *MoSOM1* and *MoCDTF1* was determined by qRT-PCR in *Δmac1*, *ΔcpkA*, *ΔmagA*, *ΔmagB* and *Δrgs1* mutants (Table S1). For comparison, other signaling mutants impaired in infection-related morphogenesis, such as *Δpmk1* and *Δmps1*, were also used. Interestingly, we found that expression levels of *MoSOM1* and *MoCDTF1* were significantly reduced in *Δmac1*, *ΔcpkA* and *ΔmagA* mutants (*P<0.01*), but not in other mutants (Figure S10). However, qRT-PCR analysis showed that expression of *MoCDTF1* was not significantly regulated by the deletion of *MoSOM1* or vice versa (data not shown). These results indicate that expression of *MoSOM1* and *MoCDTF1* are down-regulated by impairment of the cAMP/PKA signaling pathway.

To understand whether over-expression of the *MoSOM1* can restore the phenotypes of the *Δmac1* or *ΔcpkA* mutants, we developed two strains (OM1 and OM4) expressing *MoSOM1-GFP* driven by the TrpC promoter from *A. nidulans* in the *Δmac1* mutant, and similarly constructed two strains (OC2 and OC7) in the *ΔcpkA* mutant. Strong fluorescence was observed at the nucleus of these strains (Figure S11A). However, the phenotypes of the *Δmac1* or *ΔcpkA* mutants, including appressorium formation and pathogenicity (Figure S11B and C), were not restored by over-expression of *MoSOM1*. Interestingly, treatment of SC3 with the adenylate cyclase inhibitor MDL-12,330A hydrochloride at high concentrations, led to some accumulation of MoSom1-GFP in the cytoplasm (Figure S12). These results provide evidence that phosphorylation of MoSom1 by activated CpkA may be important for its nuclear localization.

### MoSom1 can physically interact with MoCdtf1, MoStu1 and CpkA

**Figure 9:**
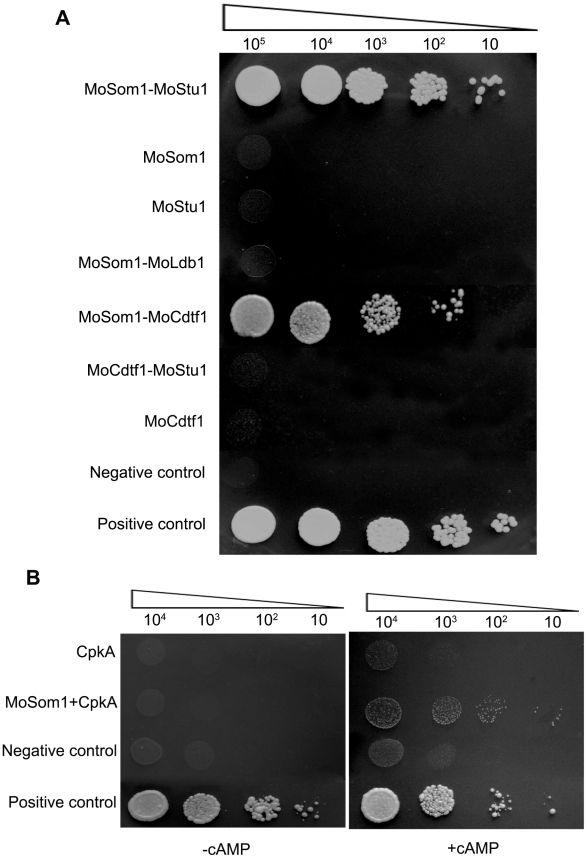
MoSom1 interacts with MoStu1, MoCdtf1 and CpkA in *M. oryzae*. (A) Strong interaction between MoSom1 and MoStu1 (an APSES protein) and MoCdtf1 in yeast two-hybrid assays. The Leu^+^ and Trp^+^ yeast transformants were assayed for growth on SD-Trp-Leu-His-Ade medium at specified concentrations 1×10^5^, 1×10^4^, 1×10^3^, 1×10^2^ and 10 cells each 10 µl droplet. (B) Weak interaction between MoSom1 and CpkA in a yeast two-hybrid assay with the presence of 5 mM cAMP. Transformants were tested for growth on SD-Trp-Leu-His-Ade medium with or without 5 mM cAMP.

Our results showed that the phenotypes of *Δmosom1* and *Δmocdtf1* mutants are somewhat similar and that expression of both *MoSOM1* and *MoCDTF1* are regulated by the cAMP/PKA signaling pathway. In a previous report, MoStu1 (MGG_00692), an APSES protein of *M. oryzae*, was shown to be required for pathogenicity and sporulation [31]. To determine whether MoSom1 interacts with the two transcription factors, MoCdtf1 and MoStu1, we carried out yeast two hybrid (Y2H) experiments. The results provided evidence that MoSom1 physically interacts with MoCdtf1 and MoStu1 (Figure 9A), suggesting both MoCdtf1 and MoStu1 were regulated by a direct interaction with MoSom1. However, we did not observe interactions between MoSom1 and other tested proteins, including CpkA and MoLdb1, under these experimental conditions. Additionally, interactions between MoCdtf1 and MoStu1 or MoLdb1 were also not observed in Y2H.

Previously, an interaction between *C. albicans* Flo8 and Tpk2 was observed in a modified Y2H system [23]. To examine the interaction between MoSom1 and CpkA, we added 5 mM exogenous cAMP into yeast growing medium. Interestingly, a weak interaction between MoSom1 and CpkA was detected by addition of 5 mM exogenous cAMP which may potentially reduce the binding of PKA catalytic subunits with regulatory subunits, while no interaction was detected between the two proteins without adding exogenous cAMP (Figure 9B), presumably because the CpkA is inactive and tightly bound to the endogenous PKA regulatory subunit. These results further demonstrate that MoSom1 may act downstream of the cAMP/PKA pathway in *M. oryzae*.

### Analysis of global patterns of gene expression in *Δmosom1* mutants of *M. oryzae*

**Figure 10:**
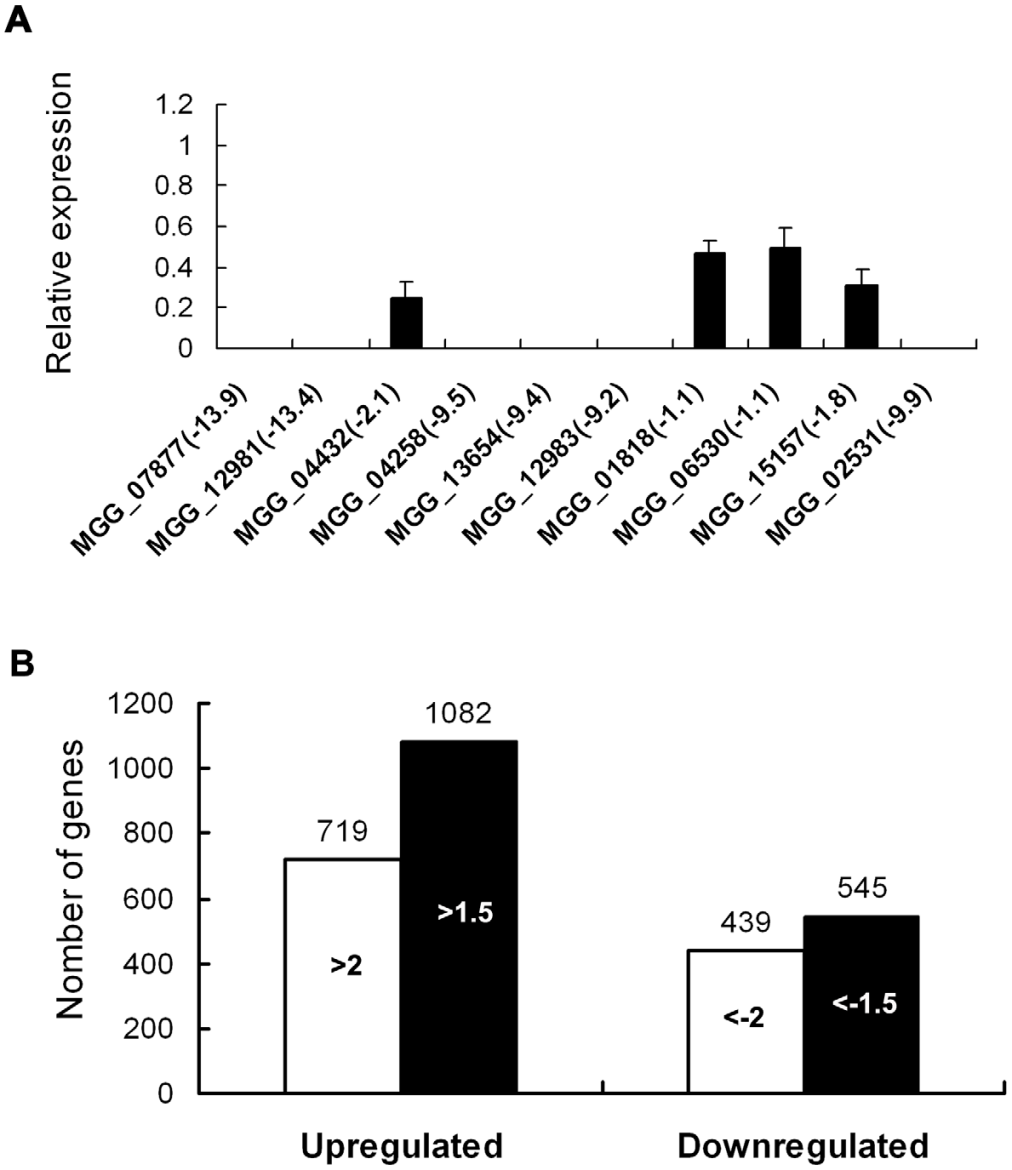
Differential gene expression analysis on transcriptomes of the *Δmosom1* mutant and Guy11 strains. (A) qRT-PCR validated the SAGE results. qRT-PCR was carried out to confirm the SAGE results through random selection of ten genes that were down-regulated in the *Δmosom1* mutant (SK27). The value of log2 ratio (*Δmosom1*/Guy11) is in bracket after gene ID number. The level of gene expression in Guy11 was taken as 1 and the relative expression in SK27 mutant was normalized based on 1. Error bars represent the standard deviation. (B) Numbers of altered genes expressing in *Δmosom1* mutant. >2.0 or >1.5, genes whose expression were up-regulated as indicated by expression profiling and the log2 ratio (*Δmosom1*/Guy11) values were more than 2 or 1.5; <−2 or <−1.5, genes whose expression were down-regulated as indicated by expression profiling and the log2 ratio (*Δmosom1*/Guy11) values were less than −2 or −1.5.

To identify genes that are putatively regulated by *MoSOM1*, we generated serial analysis of gene expression (SAGE) libraries for the wild-type strain (Guy11, 3728956 tags) and the *Δmosom1* mutant SK27 (3449284 tags) using mycelium grown in liquid CM medium. To confirm gene expression patterns derived from the SAGE libraries, 10 down-regulated genes in the *Δmosom1* mutant were randomly selected and validated by qRT-PCR. The results showed that each gene expression pattern was consistent with that in the SAGE data (Figure 10A). To identify genes that were subjected to regulation by MoSom1, we compared the gene expression profiles between the wild-type strain and the MoSom1 mutant. In total, 719 genes were up-regulated with log2 ratio (*Δmosom1*/Guy11) >2 and 439 genes were down-regulated with log2 ratio (*Δmosom1*/Guy11)<−2 (Figure 10B). Genes regulated by deletion of *MoSOM1* with log2 Ratio (*Δmosom1*/Guy11)>1.5 or <−1.5 were shown in Table S3. By analysis of the SAGE data, we found that several pathogenicity-related genes (*MPG1*, *MoVPR1*, *MoAAT1*, *MSP1*, *MoSSADH*, *MoACT*, and *COS1*) were significantly down-regulated, whereas some (*MoRIC8*, *MAC1*, *CPKA*, *MgRAC1*, *BUF1* and *TPS1*) were up-regulated (Table 1). The expression patterns of these genes by SAGE were consistent with those by qRT-PCR analysis (Table 1). Interestingly, most genes involved in the cAMP/PKA pathway, including *MAC1* and *CPKA*, were significantly up-regulated by deleting *MoSOM1* (Table 1), suggesting that MoSom1 is a negative regulator of their transcription.

**Table 1:** SAGE analysis for several known pathogenicity-related genes.

		Log_2_ Ratio				
Gene_ID	Description	*Δmosom1*/WT		*Δmoric8*/WT	*Δmoldb1*/WT	Reference
MGG_10315	Class I hydrophobin (Mpg1)	−11.06(0)(a)	−9.80(b)	−1.47(0)	−11.23(1.13E-07)	[58]
MGG_14008	Regulator of G-protein signaling (MoRic8)	2.04(3.00E-11)	2.06	ND(c)	ND	[56]
MGG_09898	Adenylate cyclase (Mac1)	5.03(0.0001237)	5.25	2.83(0.0001989)	ND	[12]
MGG_06368	Catalytic subunit of the cAMP-dependent protein kinase (CpkA)	2.04(3.87E-09)	2.33	ND	−2.76(0.0002804)	[13], [14]
MGG_02731	Rho-family GTPases (MgRac1)	1.85(0)	1.80	0.77(1.41E-11)	ND	[82]
MGG_02252	Tetrahydroxynaphthalene reductase (Buf1)	1.55(0)	1.61	3.59(4.01–197)	3.17(5.31E-132)	[83]
MGG_02531	Minor extracellular protease (MoVpr1)	−9.95(3.11E-11)	−9.21	−3.60(7.09E-05)	−4.17(0.0001121)	[71]
MGG_03860	Trehalose-6-phosphate synthase (Tps1)	1.55(1.29E-13)	1.58	ND	−0.97(3.07E-06)	[84]
MGG_01662	4-aminobutyrate aminotransferase (MoAat1)	−4.84(6.21E-23)	−5.16	−1.37(4.27E-07)	−2.04(6.58E-10)	[71]
MGG_05344	Snodprot1 homolog (Msp1)	−5.13(0)	−5.02	−4.81(0)	−3.36(2.31E-13)	[85]
MGG_01230	Succinate-semialdehyde dehydrogenase (MoSsadh)	−2.68(3.44E-110)	−2.35	0.48(2.16E-12)	ND	[71]
MGG_15157	Acetyltransferase (MoAct)	−1.77(1.99E-13)	−1.70	−0.77(0.0001066)	0.69(6.19E-06)	[71]
MGG_03977	Hypothetical protein-conidiophore stalk-less1 (Cos1)	−3.62(4.05E-19)	−3.81	ND	−2.79(2.13E-07)	[86]

(a) p-Value is indicated in brackets.

(b) Gene expression determined by quantitative RT-PCR.

(c) ND, no data available.

Recently, we have described two pathogenicity-related genes, *MoRIC8* and *MoLDB1* [56], [60]. MoRic8 interacts with Gα subunit MagB and acts upstream of the cAMP/PKA pathway to regulate infection-related morphogenesis. MoLdb1 is a morphogenetic regulator and the *Δmoldb1* mutants are similar phenotypes to the *Δmosom1* mutants. Therefore, we also generated SAGE libraries from the *Δmoric8* mutant Q-10 (3636867 tags) and the *Δmoldb1* mutant AK58 (3615472 tags). Sixty most up- or down-regulated genes in the SAGE library of *Δmosom1*, which were also detected in the SAGE libraries of the *Δmoric8* and *Δmoldb1* mutants, were presented in Table S4. As expected, the profile of gene expression in the *Δmoric8* mutant was very consistent with that in the *Δmosom1* mutant SK27, because both MoRic8 and MoSom1 proteins appear to be involved in the cAMP/PKA signaling pathway. Interestingly, the gene expression profiling of the *Δmoldb1* mutant was also consistent with that in *Δmoric8* or *Δmosom1* mutants, although there were interesting differences such as the expression of *CPKA*, *TPS1* and *MoACT*, as shown in Table 1. These data suggest that there may be a potential link between MoSom1 and MoLdb1 in regulating infection-associated gene expression in *M. oryzae*.

## Discussion

In this study we identified three T-DNA insertional mutants, YX-145, YX-1303 and YX-864, which were defective in multiple steps of plant infection and morphogenesis by the rice blast fungus *Magnaporthe oryzae*. HiTAIL-PCR analysis revealed the integrated T-DNA in the mutants disrupted genomic regions corresponding to genes of *MoSOM1*, *MoCDTF1* and *MoMSB2*, respectively. Targeted deletion of *MoSOM1* or *MoCDTF1* caused severe defects in both fungal morphogenesis and virulence, which were consistent with the corresponding T-DNA insertional mutants (Figure 2–4). To our knowledge, both *MoSOM1* and *MoCDTF1* genes have not been functionally characterized previously in phytopathogenic fungi. In addition, our results also showed that *MoMSB2* was required for plant infection-related morphogenesis and virulence in *M. oryzae*, which is consistent with a very recent study in which the gene was independently identified [49]. However, we also observed that deletion of *MoMSB2* resulted in a significant reduction in conidiation (Figure S4A), which was distinct from the previous report.

MoSom1 and MoCdtf1 are key morphogenetic regulators. Like most fungal pathogens, asexual reproduction and infection-related development play key roles in the disease cycle in *M. oryzae* [39]. Molecular genetic analysis of conidiation reveals several conidiation-associated genes that have distinct effects on control of conidiation and conidial morphology. The *con7* mutant, for instance, produces a mixture of normal and aberrantly shaped conidia unable to form appressorium, and is non-pathogenic [61]. However, very few mutants have been identified that have completely lost the ability to form conidia in *M. oryzae*. The *MoHOX2* gene encodes a putative homeobox transcription factor. Deletion mutants of *MoHOX2* completely abolished asexual sporulation, but the mutants were still pathogenic through hypha-driven appressoria [62], [63]. Recently, we have reported that *MoLDB1* gene encoding a protein with a putative LIM binding domain is necessary for fungal morphogenesis [60]. Deletion mutants of *MoLDB1* completely lost the ability to differentiate spores, including meiotically generated ascospores, and were non-pathogenic. The mutants were also unable to differentiate conidiophores or appressoria from mycelium [60]. One of the most interesting findings we report here is that deletion either *MoSOM1* or *MoCDTF1* completely blocked asexual/sexual sporulation and appressorium development from mycelium and the mutants were non-pathogenic. Interestingly, similar to *MoLDB1*, both *MoSOM1* and *MoCDTF1* are also required for efficient hyphal growth, melanization and hydrophobicity. Furthermore, we did not observe conidiophores in the mutants, indicating that the defect in conidiation of the mutants is associated with lack of conidiophore formation rather than subsequent conidiogenesis.

*M. oryzae* MoSom1 is homologous with *Aspergillus nidulans* OefA and the hypothetical proteins from other related fungal species. Among these proteins in filamentous fungi, only *A. nidulans* OefA has been investigated [64]. *OEFA* has been identified by gene silencing and over-expression approaches and targeted deletion of *OEFA* causes a “fluffy” growth phenotype due to its development of undifferentiated aerial hyphae [64]. However, the detailed role of OefA in signaling pathways has not been characterized. In yeasts, previous studies have shown that *Saccharomyces cerevisiae* Flo8 is critical for filamentous growth and functions downstream of the cAMP-PKA pathway [18], [19], [23]. Similarly, *Candida albicans* Flo8 is also essential for hyphal development and virulence and functions downstream of the cAMP-PKA pathway [28]. Since MoSom1 showed only 14.76% and 14.93% amino acid identity with *S. cerevisiae* Flo8 and *C. albicans* Flo8, respectively, this makes it difficult to find orthologs of Flo8 from the genomes of filamentous fungi by BLAST search. As a consequence of this, a recent report mentioned that the *M. oryzae* genome, including many other filamentous ascomycetes, may lack distinct orthologs of Flo8 [65]. However, we have shown in this report that MoSom1 functions downstream of the cAMP/PKA pathway, in a similar manner to yeast Flo8. Several lines of evidence support such a view. First, *MoSOM1* can complement a *S. cerevisiae flo8* mutant in its ability to carry out haploid invasive and diploid pseudohyphal growth. Second, a strong interaction between MoSom1 and MoStu1 and a weak interaction between MoSom1 and CpkA was detected by yeast two-hybrid analysis. Thirdly, *MoSOM1* expression was significantly down-regulated by deletion of *MAC1* or *CPKA*, the two key components of the cAMP/PKA pathway, and finally, the defects of *Δmosom1* mutants could not be restored by supplementation with exogenous cAMP.

MoSom1 directly interacted with MoStu1 in a yeast two-hybrid assay, and might therefore act as a regulator of MoStu1 to regulate fungal morphogenesis in *M. oryzae*. In *C. albicans*, Efg1, an APSES transcription factor, is essential for regulating morphogenesis [66]. A previous report has demonstrated that *C. albicans* Flo8 interacts with Efg1 to regulate expression of hypha-specific genes and genes important for virulence [28]. In *M. oryzae*, MoStu1 is also an APSES transcription factor [31]. Deletion of *MoSTU1* results in a reduction of mycelial growth and conidiation and a delay in appressorium formation, and deletion mutants are non-pathogenic [31]. Consistently, we also found that a strong interaction between *M. oryzae* MoSom1 and MoStu1 in a yeast two-hybrid assay, indicating that MoSom1 may act as a regulator of MoStu1 to regulate fungal morphogenesis. However, because of the different phenotypes of *Δmosom1* and *Δmostu1* mutants, it seems reasonable to predict that MoSom1 also interacts with other transcription factors in addition to MoStu1.

In a previous study, a direct interaction between *S. cerevisae* Flo8 and Tpk2 proteins was observed using a modified yeast two-hybrid system carried out in the presence of exogenous cAMP [23]. We found a weak interaction between *M. oryzae* MoSom1 and CpkA but only when the selection medium was supplemented with 5 mM exogenous cAMP (Figure 9B). This analysis makes a prediction possible that directly places MoSom1 downstream of the cAMP/PKA signaling. In *S. cerevisiae*, phosphorylation of Flo8 by Tpk2 is required for Flo8 interaction with the *FLO11* promoter both *in vivo* and *in vitro* [23]. Since multiple PKA phosphorylation sites were also predicted in the MoSom1 protein (see Figure S8B), therefore, in addition to transcriptional regulation, it is possible that MoSom1 is activated by serine/threonine phosphorylation by CpkA to regulate genes required for fungal morphogenesis and pathogenicity. Additionally, we noted that there were obvious different phenotypes between *Δmosom1* and *ΔcpkA*. It is therefore also possible that MoSom1 may be activated by additional regulators from different signaling pathways.

LisH domains exist in various eukaryotic proteins and are required for regulating microtubule dynamics, either by mediating dimerization, or by binding cytoplasmic dynein heavy chain or microtubules directly [67]. Like yeast Flo8, *M. oryzae* MoSom1 has a LUFS domain with a conserved LisH motif at its N-terminus (Figure S9A). Multiple alignment analyses indicated that the LisH domain is highly conserved in fungi (Figure 7A). We found that the LisH domain is required for the function of MoSom1 in *M. oryzae*, because deletion of the LisH domain in *MoSOM1* partially impaired protein localization to the nucleus and resulted in similar phenotypes to the *Δmosom1* mutant (Figure 7C). It is possible that the LisH domain may therefore mediate cytoskeletal interactions necessary for transport of MoSom1 to the nucleus. In *S. cerevisiae* Flo8 has been localized to the nucleus [18]. Consistent with this, our results also showed that MoSom1 localized to the nucleus and that the predicted NLS of PSKRVRL is important for the function and transportation of MoSom1 protein from the cytoplasm to the nucleus.

In this study, we also found the expression of *M. oryzae MoSOM1* was significantly down-regulated by deletion of either *MAC1* or *CPKA* (Figure S10) , which encode the key components of the cAMP/PKA pathway and, interestingly, several genes involved in the cAMP/PKA pathway were significantly up-regulated after deletion of *MoSOM1* (Table 1). These data are also consistent with MoSom1 acting downstream of the cAMP/PKA pathway.

When considering these results together, we conclude that MoSom1 is likely to act as a transcriptional regulator that functions downstream of the cAMP/PKA pathway to regulate fungal morphogenesis and pathogenicity.

*M. oryzae* appears to possess over 400 transcription factor genes, but only a minority of them have so far been characterized, including *MST12* [68], *CON7* [61], *MIG1* [69], *MoHOX8* [62], *COM1* [70], *MoAP1* [71] and *MoMCM1* [72], which are required for fungal morphogenesis or plant infection by *M. oryzae*. In this study, we identified a novel transcription factor, MoCdtf1, which is essential for sporulation, apressorium formation and virulence. However, *ΔmoCdtf1* mutants were able to cause some disease on wounded leaves or roots, although the disease severity was significantly reduced compared with the isogenic wild-type strain or complemented strains (Figure 2). These results were consistent with a recent report, in which an insertional mutant M558 was presented in which the T-DNA was integrated into the promoter of *MoCDTF1* and also showed impairment in conidiation and pathogenicty, but still infected rice roots [73]. MoCdtf1 has a putative NLS sequence and a conserved zinc finger structure, which are important for MoCdtf1 protein localized to nucleus and for regulating plant infection-related mophorgenesis. Like *MoSOM1*, expression of *M. oryzae MoCDTF1* was significantly down-regulated by deletion of either *MAC1* or *CPKA* (Figure S10). More interestingly, we found that MoCdtf1 physically interacts with MoSom1 in a yeast two hybrid assay (Figure 9A). These data suggest that *M. oryzae* MoCdtf1 may function as a transcription factor that acts downstream of the cAMP/PKA pathway.

The importance of MoSom1 to infection-related development was underlined by transcriptional profile analysis using SAGE, which demonstrated that a large set of genes are differentially regulated in a *Δmosom1* mutant compared to a wild type *M. oryzae* strain. Significantly, morphogenetic genes, such as the *MPG1* hydrophobin gene and the *BUF1* melanin biosynthesis gene, as well as physiological regulators such as the *TPS1* trehalose-6-phosphate synthase gene were among those differentially regulated. This is consistent with MoSom1 affecting processes pivotal to the formation and function of appressoria and acting downstream of the cyclic AMP signaling pathway, which is necessary for infection-related development in rice blast. The pleiotropic effects of the *Δmosom1* mutation on mycelial growth rate do, however, suggest that some of the observed major changes in gene expression may be a consequence of the slower growth rate and aberrant mycelial morphology of *Δmosom1* mutants. Dissecting specific families of genes regulated by the moSom1 pathway during appressorium development will therefore be important in elucidating the underlying biological processes regulated by this signaling mechanism.

**Figure 11:**
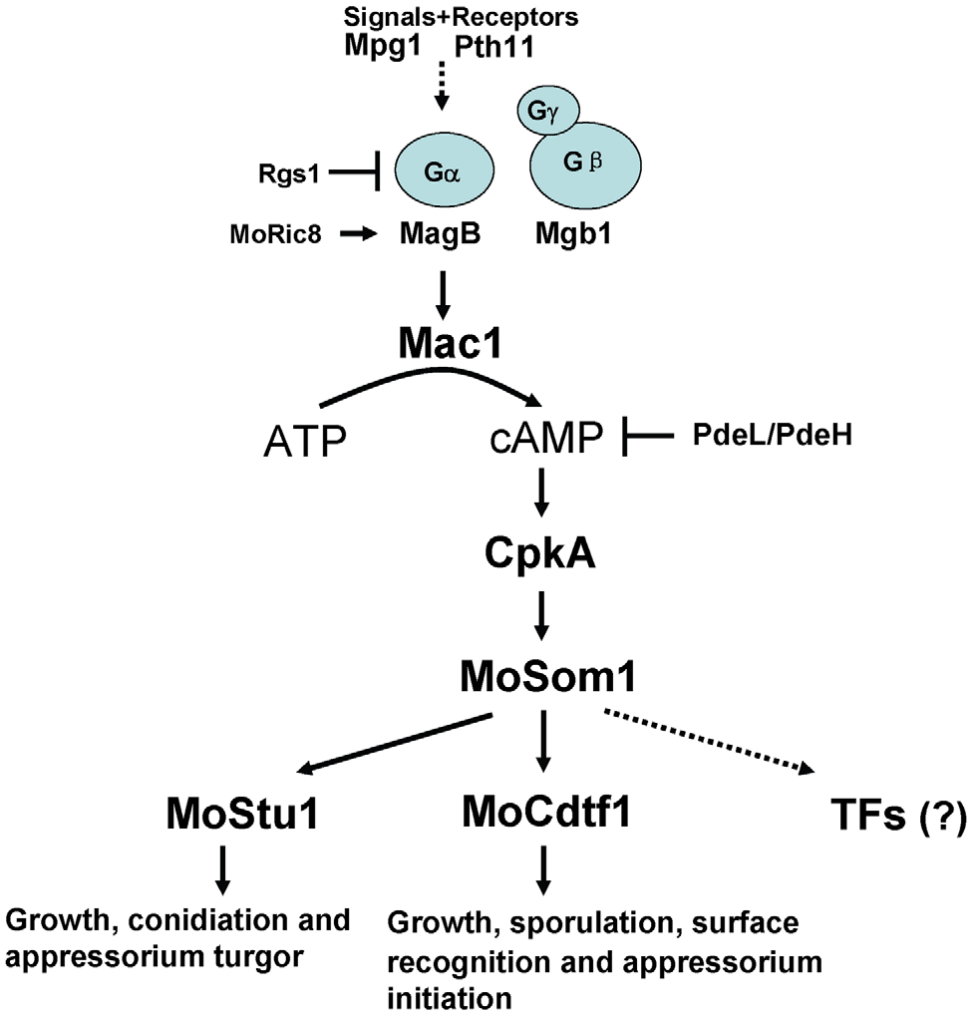
Model of the cAMP/PKA signaling pathway in *M. oryzae*. In this model, the rice blast fungus responds to external physical cues from the rice leaf surface that are detected by receptors such as Pth11 and are transmitted via the heterotrimeric G-protein leading to Mac1 adenylate cyclase activation. This activates the cyclic AMP dependent protein kinase A, CpkA, which in turn acts upstream of MoSom1, a transcriptional regulator that acts through a set of transcription factors including MoStu1, MoCdtf1 and others to bring about infection-assocaited development.

In summary, based on results from this report, we have developed a model of the cAMP/PKA signaling pathway in *M. oryzae* that is shown in Figure 11. Surface recognition and initiation of appressorium formation is regulated by the pathway. Moreover, the cAMP/PKA pathway is also involved in regulation of hyphal growth, asexual/sexual sporulation and invasive growth in host tissues. Free CpkA may activate MoSom1 protein to regulate appressium turgor generation through MoStu1 and to control sporulation and appressorium formation through MoCdtf1. However, it is also possible that additional transcription factors are regulated by MoSom1 to control these developmental processes. The model will allow us to test the wider roles of the cAMP/PKA pathway in regulating fungal morphogenesis and plant infection in *M. oryzae* in future.

## Materials and Methods

### Strains, culture conditions and molecular manipulations of DNA and RNA

All mutants described in the present study were generated from the *Magnaporthe oryzae* wild-type strain Guy11 [74], and are listed in Table S1. Standard growth and storage procedures for fungal strains were performed, as described previously [58]. *A. tumefaciens* AGL1 was used for T-DNA insertional transformation. *Escherichia coli* strain DH-5α was used for routine bacterial transformations and maintenance of various plasmids in this study. Southern blot analysis was performed by the digoxigenin (DIG) high prime DNA labeling and detection starter Kit I (Roche, Mannheim, Germany). General procedures for nucleic acid analysis followed standard protocols [75].Total RNA was extracted from mycelium of *M. oryzae* using the SV Total RNA Isolation System (Z3100; Promega Corp.) according to the manufacturer's instructions.

### Construction of vectors and fungal transformation

For construction of the gene replacement vector pMoSOM1-KO (Figure S3C), 1.4 kb (left border) and 1.2 kb (right border) flanking sequences of the *MoSOM1* gene locus were amplified using primer pairs of 3F/4R and 5F/6R (Table S2; Figure S3C) and cloned sequentially into pGEM-T easy vectors to generate pGEM-145L and pGEM-145R, respectively. The 1.4 kb *HPH* gene cassette, which encodes hygromycin phosphotransferase under control of the *A. nidulans* TrpC promoter [76], was amplified with primers HPH-Kpn-F and HPH-Xba-R (Table S2) using pCB1003 as a template and clone into pGEM-T easy vectors to give pGEM-HPH. The pGEM-HPH was digested with *Kpn*I and *Apa*I and inserted the fragment from pGEM-145R with the same digestions to generate pGEM-HPH-R. The pMoSOM1-KO was constructed by insertion *Spe*I and *Xba*I fragment from pGEM-145L into corresponding site of pGEM-HPH-R. To construct complementation vector pMoSOM1-GFP, a 4.3 kb fragment including 2.8 kb *MoSOM1* gene-coding sequence and a 1.5 kb promoter region were amplified using primers 145H-Nde-F and 145H-Hind-R (Table S2) and then cloned into pGEM-T easy vectors to produce pGEM-SOM. The pMoSOM1-GFP was generated by ligation of pGEM-SOM with the 1.5 kb GFP allele, which was amplified using primers GFP-Hind-F and GFP-Xho-R (Table S2). The pMoSOM1-DKO vector was constructed by replacing the *HPH* of pMoSOM1-KO with a 0.94 kb *bar* gene cassette encoding phosphinothricin acetyl transferase under control of the *A. nidulans* TrpC promoter, which was amplified with primers Bar-Xba-F and Bar-Kpn-R (Table S2) using pMLH21-bar [77] as a template. The *MoSOM1* over-expression vector, pOE-MoSOM1, was constructed by insertion the 4.3 kb fragment (2.8 kb *MoSOM1* gene-coding sequence and 1.5 kb GFP cassette) , which was amplified with the primers 145OE-Xho-F and GFP-Xho-R (Table S2) using the pMoSOM1-GFP as a template, into the corresponding site of pCB1532 with the *A. nidulans* trpC promoter.

A similar strategy was used to construct the gene replacement vector pMoCDTF1-KO. About 1.2 kb (left border) and 1.5 kb (right border) flanking sequences *MoCDTF1* gene locus were amplified using primer pairs of 7F/8R and 9F/10R (Table S2; Figure S3E) and cloned sequentially into pGEM-T easy vectors to generate pGEM-1303L and pGEM-1303R, respectively. The pGEM-1303R was digested with *Sac*I and *Xba*I and the released fragment was inserted into the corresponding site of pGEM-HPH to produce pGEM-1303HR. The pGEM-1303HR was digested with *Kpn*I and *Apa*I and then inserted with the fragment liberated from pGEM-1303L to generate pMoCDTF1-KO. To construct complementation vector pMoCDTF1-GFP, a 5.7 kb fragment including 4.1 kb *MoCDTF1* gene-coding sequence and a 1.6 kb promoter region were amplified using primers 1303H-Aat-F and 1303H-Kpn-R (Table S2) and then cloned into pGEM-T easy vectors to produce pGEM-CDTF. The pMoCDTF1-GFP was generated by ligation of pGEM-CDTF with the 1.5 kb GFP allele, which was amplified using primers GFP-Kpn-F and GFP-Xho-R (Table S2).

To construct the *MoMSB2* gene replacement vector pMoMSB2-KO (Figure S3A), a 4.2 kb fragment spanning the *MoMSB2* locus was amplified with primers 1F and 2R (Table S2) and cloned into pGEM-T easy vector (Promega, Madison, WI, U.S.A.), and a 1.7 kb *Xho* I and *Spl* I fragment containing the majority of the *MoMSB2* ORF was removed and replaced sequentially with the 1.4 kb *HPH* gene cassette amplified with primers HPH-Spl-F and HPH-Xho-R (Table S2) using pCB1003 as a template. For construction of complementation vector pMoMSB2-HB, a 4.2 kb fragment including 2.4 kb *MoMSB2* gene-coding sequence and a 1.8 kb promoter region were amplified using primers 864H-Sal-F and 864H-Spe-R (Table S2) and then cloned into pGEM-T easy vectors to produce pMoMSB2-HB.

For deletion of the *MAC1* gene, the gene deletion vector pMoMAC1-KO was generated using a similar strategy to pMoMSB2-KO. A 4.8 kb fragment spanning the *MoMAC1* locus was amplified with primers MAC-KO-FP/MAC-KO-RP (Table S2) and cloned into pGEM-T easy vector to give pGEM-MAC1. The *HPH* gene cassette was amplified with the primers HPH-Hind-F and HPH-Hind-R (Table S2) using PCB1003 as a template. The pMoMAC1-KO was constructed by insertion *HPH* gene cassette with *Hin*dIII ends into the corresponding restriction site of pGEM-MAC1. The vector for deletion of *MAGA* gene was kindly provided by professor Hao Liu, Tianjin University of Science and Technology.

The resulting vectors were linearized and transformed into *M. oryzae* Guy11 protoplasts to generate gene null mutants, respectively, as previously described [58]. Together with pCB1532 [78] vectors, the complementation vectors, pMoSOM1-GFP, pMoCDTF1-GFP and pMoMSB2-HB, were used to co-transform into their corresponding mutants, respectively. The vector pOE-MoSOM1 was used to transform *Δmac1* and *ΔcpkA* mutants to generate strains that *MoSOM1* was over-expressed, respectively. GFP fluorescence was observed using a Leica TCS SP5 inverted confocal laser scanning microscope (Leica, Wetzlar, Germany).

### Construction of *MoSOM1* ^ΔLisH^, *MoSOM1* ^ΔPKKK^, *MoSOM1* ^ΔPSKRVRL^ and *MoCDTF1* ^ΔPPKRKKP^ mutants

Three rounds of PCR amplification were carried out for the construction of pMoSOM1^ΔLisH^-GFP described as follows. First, 1.6 kb and 4.0 kb fragments were amplified with the primer pairs of 145H-Nde-F/LisH-R and LisH-F/GFP-Xho-R (Table S2) using pMoSOM1-GFP as a template, respectively. Second, the two PCR products were mixed and performed PCR reaction (10 reaction cycles) without adding primers. Third, a 5.6 kb fragment containing 1.5 kb native *MoSOM1* promoter, 2.6 kb *MoSOM1* gene-coding sequence (without Lish domain) and 1.5 kb GFP cassette was amplified by the primers 145H-Nde-F and GFP-Xho-R (Table S2) using the mixture as a template. Finally, the pMoSOM1^ΔLisH^-GFP was generated by insertion of the 5.6 kb fragment into pGEM-T easy vector.

A similar strategy was used to construct pMoSOM1^ΔPKKK^-GFP and pMoCDTF1^ΔPPKRKKP^-GFP vectors. The pMoSOM1^ΔPKKK^-GFP was generated from pMoSOM1-GFP using primer pairs of 145H-Nde-F/PKKK-R and PKKK-F/GFP-Xho-R (3.7 kb and 2.1 kb PCR products, respectively), whereas the pMoCDTF1^ΔPPKRKKP^-GFP was generated from pMoCDTF1-GFP using primer pairs of 1303H-Aat-F/1303CD-R and 1303CD-F/GFP-Xho-R (4.8 kb and 2.4 kb PCR products, respectively). The pMoSOM1^ΔPSKRVRL^-GFP was constructed by self-ligation of the PCR products amplified with primers PSK-F and PSK-R (Table S2) using pMoSOM1-GFP as a template. The primers used for the constructions were listed in Table S2.

The pMoSOM1^ΔLisH^-GFP, pMoSOM1^ΔPKKK^-GFP, pMoSOM1^ΔPSKRVRL^-GFP were used to transform the *Δmosom1* mutants to generate *MoSOM1* ^ΔLisH^, *MoSOM1* ^ΔPKKK^, *MoSOM1* ^ΔPSKRVRL^, respectively. The pMoCDTF1^ΔPPKRKKP^-GFP was used to transform *Δmocdtf1* to produce *MoCDTF1* ^ΔPPKRKKP^.

### Pathogenicity assays

For cut-leaf assays, fragments were cut from the leaves of 10-day-old barley cv Golden Promise and 14-day-old rice cv CO-39 seedlings, both highly susceptible toward *M. oryzae*, and placed in plastic plates containing wetted filters. Mycelium from 2-day-old liquid CM cultures at 25°C was placed onto leaf sections and the plates were incubated in a cycle of 12 h of light and 12 h of dark at 25°C. Wounded leaves were prepared by removing the surface cuticle by abrasion with an emery board as described previously [79]. For spray-inoculation assays, conidial suspensions were diluted in 0.2% gelatin to 1×10^5^ conidia ml^−1^ for rice infections using rice cv. CO-39. Conidia were spray-inoculated using an artist's airbrush onto 14-day-old plants. Rice seedlings were incubated in plastic bags for 24 h to maintain high humidity and then transferred to controlled environment chambers at 25°C and 90% relative humidity with illumination and 14 h light periods. For root infection assays, rice seeds were germinated for 3 days at 28°C and then transferred to plates contained 2% water agar. Mycelial plugs were carefully placed rice roots. Each test was repeated three times. Disease lesions were examined and photographed after 5 days of incubation.

### Analysis of fungal growth, sporulation, appressorium formation and genetic crosses

Vegetative growth was assessed by measurement of colony diameter on plate cultures of *M. oryzae* grown on CM. For mycelium dry weight assays, the same size blocks (1×1.5 cm^2^) cut from 7-day-old CM cultures were blended and inoculated in flasks containing 150 ml liquid CM medium. The flasks were incubated at 25°C for 2 days (150 rpm). After incubation, the mycelia produced in liquid cultures were filtered and washed. The dry weight of each mycelium was determined after drying at 60°C for 24 h. Three replicates of each treatment were performed, and the experiment was repeated three times.

Conidial development was assessed by harvesting conidia from the surface of 10-day-old plate cultures and by determining the concentration of the resulting conidial suspension using a haemocytometer. Appressorium development was assessed by allowing conidia at a concentration of 1×10^4^ conidia ml^−1^ to germinate on hydrophobic GelBond films or onion epidermis and incubating them in a humid environment at 25°C. For appressorium formation from the tips of mycelia, mycelia of the wild-type strain Guy11 and mutant strains were harvested from 48 h liquid CM cultures, and the mycelium fragment suspensions were placed on hydrophobic GelBond film surfaces to allow appressorium development. Appressorium formation was observed after 24 h incubation at 25°C in darkness. Fertility assays were carried out by pairing Guy11 (*MAT1-2*) and tested strains with standard tester strain TH3 (*MAT1-1*) on oatmeal agar (OMA) plates, as described previously [60]. Each test was repeated three times.

### Quantitative RT-PCR analysis

Total RNA was utilized for synthesis of the first strand cDNA using the PrimeScript™ 1st Strand cDNA Synthesis Kit (D6110A, TaKaRa, Tokyo). The resultant cDNA was used as a template for quantitative RT-PCR (qRT-PCR). qRT-PCR was performed with a SYBR Green Realtime PCR Master Mix Kit (QPK-201, TOYOBO, Osaka, Japan) using an iCycler iQ™ Multicolor Real-Time PCR Detection System (Bio-Rad, Munich, Germany). All qRT-PCR reactions were conducted in triplicates for each sample and the experiment was repeated three times. *M. oryzae* beta-tubulin gene (MGG_00604) amplified with the primer pairs of BT-F/BT-R was used as an endogenous reference. The abundance of the gene transcripts was calculated relative to this control using the 2^−ΔΔCT^ method [80]. All the primers used for qRT-PCR were listed in Table S2.

### Yeast complementation analysis

Yeast complementation was carried out as described previously [28]. The full length cDNA of *MoSOM1* was amplified with primers SOM-E-F and SOM-Xh-R and cloned into pYES2 vector to generate pYES2-SOM1. The yeast expression vector pYES2-SOM1 was transformed into the haploid mutant HLY850 and the diploid mutant HLY852 of *S. cerevisiae*, respectively. The transformants grown on SD-Ura plates were selected to test the ability of invasive growth on YPD plate and the pseudohyphal growth on SLAD plate supplemented with galactose. The yeast strains, MY1384 (*MATa* wild type), HLY850 (*MATa flo8::hisG ura3-52*), CGx68 (*MATa/α* wild type) and HLY852 (*MATa/α flo8::hisG/flo8::hisG ura3-52/ura3-52*), were kindly provided by Professor Jiangye Chen of Shanghai Institute for Biological Sciences, Chinese Academy of Sciences.

### Yeast two-hybrid (Y2H) assay

The Y2H assay was conducted according to the BD Matchmaker Library Construction & Screening Kits instructions (Clontech, PaloAlto, CA, U.S.A.). The full-length cDNA of *MoSOM1*, *MoCDTF1*, *MoSTU1*, *MoLDB1* and *CPKA* was amplified with the primer pairs SOM-E-F/SOM-Xh-R, 1303YTH-E-F/1303YTH-E-R, STU-E-F/STU-E-R, LDB-E-F/LDB-S-R and CPK-F/CPK-R (Table S2), respectively. The cDNA of *MoSOM1* was cloned into pGADT7 as the prey vector pGADT7-MoSOM1 and the other cDNAs were cloned into pGBKT7 as the bait vector, respectively. The resulting pGADT7-MoSOM1 and each bait vector were co-transformed into yeast strain AH109. The Leu^+^ and Trp^+^ yeast transformants were isolated and assayed for growth on SD-Trp-Leu-His-Ade medium. Yeast strains for positive and negative controls were from the Kit.

### Serial analysis of gene expression (SAGE)

The *M. grisea* wild-type strain Guy11 and the mutants, SK27 (*Δmosom1*), AK58 (*Δmoldb1*) [60] and Q-10 (*Δmoric8*) [56], were cultured in liquid CM medium at 28°C for 48 h in the dark (at 200 rpm). The mycelium of these strains was harvested, and total RNA was extracted using the SV Total RNA Isolation System (Z3100; Promega) according to the manufacturer's instructions. The RNA samples were then sent to Beijing Genomics Institute (BGI; Huada) for serial analysis of gene expression (SAGE).

## Supporting Information

Figure S1 **Genomic DNA flanking the integrated T-DNAs in YX-145, YX-864 and YX-1303 was amplified by hiTAIL-PCR.** High-efficiency thermal asymmetric interlaced polymerase chain reaction (hiTAIL-PCR) was performed as previously described [81]. The primers used for hiTAIL-PCR were shown in Table S2. The genomic DNAs flanking right sites of the integration T-DNAs of the YX-145, YX-864 and YX-1303 mutants were obtained from the third round products, respectively. The arrows indicated the PCR products were harvested for cloning and sequencing. M, 250 bp marker (Takara).
(TIF)

Figure S2 **Phylogenetic analysis of** ***Magnaporthe*** **MoSom1 and MoCdtf1 with the homologs from other fungal species.** (A) Phylogenetic tree of *Magnaporthe* MoSom1 and 14 homologs from other fungal species was constructed by observed divergency distance method in the program DNAMAN. Numbers at the nodes in the rooted tree represent bootstrapping value on 1000 replications. Abbreviations and numbers correspond to species names and GenBank accession numbers, respectively. Af, *Aspergillus fumigatus*; An, *A. nidulans*; Ang, *A. niger*; Ca, *Candida albicans* (Flo8); Cg, *Chaetomium globosum*; Gc, *Grosmannia clavigera*; Gg, *Glomerella graminicola*; Gz, *Gibberella zeae*; Ma, *Metarhizium acridum*; Mo, *Magnaporthe oryzae* (MoSom1); Nc, *Neurospora crassa*; Nh, *Nectria haematococca*; Pa, *Podospora anserine*; Pm, *Penicillium marneffei*; Va, *Verticillium albo-atrum*. (B) Phylogenetic tree of *Magnaporthe* MoCdtf1 and 15 homologs from other species was constructed as described above. Af, *Aspergillus fumigatus*; An, *A. nidulans*; Ang, *A. niger*; Ao, *A. oryzae*; At, *A. terreus*; Bf, *Botryotinia fuckeliana*; Cg, *Chaetomium globosum*; Gg, *Glomerella graminicola*; Gz, *Gibberella zeae*; Mo, *Magnaporthe oryzae* (MoCdtf1); Nc, *Neurospora crassa*; Nh, *Nectria haematococca*; Pa, *Podospora anserine*; Pm, *Penicillium marneffei*; Ss, *Sclerotinia sclerotiorum*; Va, *Verticillium albo-atrum*. The bar indicates 0.05 distance units. DNAMAN version 5.2.2 program was used for alignment and phylogenetic tree constrution.
(TIF)

Figure S3 **Targeted gene replacement of** ***MoMSB2*** **,** ***MoSOM1*** **and** ***MoCDTF1*** **.** (A) Construction of the vector pMoMSB2-KO and targeted gene replacement of *MoMSB2*. (B) Southern blot analysis. Genomic DNA was digested with *Hin*dIII/*Sac*I and probed with a 0.9 kb fragment amplified with the primers P864-F and P864-R. Lane 1, EM3 (ectopic); lane 2 and 3, MK9 and MK12 (*Δmomsb2*); lane 4, Guy11. (C) Construction of the vector pMoSOM1-KO and targeted gene replacement of *MoSOM1*. (D) Southern blot analysis. Genomic DNA was digested with *Sal*I and probed with a 1.2 kb fragment amplified with the primers 5F and 6R. Lane 1, Guy11; lane 2 to 4, SK5, SK21 and SK27 (*Δmosom1*); lane 4, ES16 (ectopic). (E) Construction of the vector pMoCDTF1-KO and targeted gene replacement of *MoCDTF1*. (F) Southern blot analysis. Genomic DNA was digested with *Bam*HI and probed with a 1.0 kb fragment (top) amplified with the primers P1303-F and P1303-R and a 1.4 kb *HPH* cassette (bottom), respectively. Lane 1, Guy11; lane 2, EC14 (ectopic); lanes 3 and 4, CTK2 and CTK15 (*Δmocdtf1*). (G) Construction of the double KO vector pMoSOM1-DKO and targeted gene replacement of *MoSOM1* in the *Δmocdtf1* mutant (CTK15). (H) *Δmosom1Δmocdtf1* mutants confirmed by PCR analysis. The *MoSOM1* coding sequence were amplified with primers 145-F and 145H-Hind-R in CTK15 (*Δmocdtf1*) and ESC4, but absent in D-3 and D-9 (top). A bar gene cassette could be amplified with primers Bar-Xba-F and Bar-Kpn-R from all other strains, except CTK15 (bottom). Lane 1, CTK15 (*Δmocdtf1*); lane 2 and 4, D-3 and D-9 (*Δmosom1Δmocdtf1*); lanes 3, ESC4 (transformant with ectopic integration of pMoSOM1-DKO). (I) *Δmosom1Δmocdtf1* mutants confirmed by RT-PCR analysis. 0.2 kb PCR products were amplified with primers 145Q-F and 145Q-R for CTK15 (*Δmocdtf1*) and ESC4, but absent for D-3 and D-9 (top). A 0.2 kb beta-tubulin gene (MGG_00604.6) fragment could be amplified from all the strains with primers BT-F and BT-R (bottom). A = *Apa*I; K = *Kpn*I; S = *Sal*I; Sc = *Sac*I; Sp = *Spe*I; Spl = *Spl*I; X = *Xba*I; Xh = *Xho*I. Asterisk represents restriction sites introduced or derived from vectors.
(TIF)

Figure S4 ***MoMSB2*** **is required for conidiation, appressorium formation and pathogenicity in** ***Magnaporthe oryzae*** **.** (A) Bar chart showing the conidial production of various strains. Error bars represent the standard deviation. The YX-864 and MK12 (*Δmomsb2*) mutants were reduced in conidiation on CM medium. Asterisks indicate a significant difference of conidiation between Guy11 and the mutants (*P*<0.01). Guy11, the wild type strain; YX-864, the T-DNA insertional mutant; MK12, *Δmomsb2*; EM3 (ectopic); MC5 (*Δmomsb2*+*MoMSB2*). (B) Bar chart showing appressorium formation. Conidial suspension was dropped on GelBond films to allow appressorium formation and incubated at 25°C for 24, 48, and 72 h. Percentage of conidia to form appressoria was calculated under a microscope. The *Δmomsb2* mutant (MK12) was delayed and reduced to form appressoria. Asterisks indicate a significant difference of appressorium formation between Guy11 and the mutants (*P*<0.01). (C) Guy11 formed numerous melanized appressoria at 24 h, while the MK12 mutant could hardly produce appressoria at this stage. However, the MK12 mutant could form some appressoria for 48 h and 72 h incubation. Scale bar = 10 µm. (D) Pathogenicity assays. Barley segments were inoculated with the conidial drops (10 µl per drop; 1×10^5^ conidia/ml). a = unwounded leaf and b = abraded leaf. Rice leaves were spray-inoculated with the conidia at a concentration 1×10^5^ conidia/ml. H_2_O containing 0.2% gelatin was used as the control. Photographs were taken at 5 days after inoculation.
(TIF)

Figure S5 **Growth patterns of the** ***Δmosom1*** **and** ***Δmocdtf1*** **mutants on various media.** The *Δmosom1* (SK27) and *Δmocdtf1* (CTK15) mutants reduced in vegetative growth and mycelium pigmentation on different media, CM, MM, PDA and OAM.
(TIF)

Figure S6 **Intracellular localization of MoCdtf1-green fluorescent protein.** (A) Expression of *Magnaporthe oryzae MoCDTF1* in hyphae of the strain CTC1, which carries a single GFP-carboxy translational fusion of *MoCDTF1*. The GFP fluorescence was observed in the nucleus. (B) The patterns of *MoCDTF1* expression and nuclear division during appressorium development in *M. oryzae*. Conidia of the strain CTC1 was allowed to germinate on hydrophobic GelBond film surfaces. Photographs were taken at various time intervals. BF = bright field. Scale bars = 10 µm.
(TIF)

Figure S7 **The phenotypes of** ***Δmosom1*** **mutants are restored by re-introduction of** ***MoSOM1*** **gene.** (A) The colony morphology of the complementation transformant SC3 (*Δmosom1*+*MoSOM1*) was similar with the wild type strain Guy11. All defects of the *Δmosom1* mutant (SK27), including vegetative growth, mycelium pigmented melanization, conidiation and appressorium formation, were overcome by re-introduction of *MoSOM1* gene. +, normal; +/−, significantly reduced; −, not any. (B) Conidia from SC3 geminated and formed numerous appressoria on onion epidermis. Scale bar = 10 µm.
(TIF)

Figure S8 **Putative spliced isoforms of** ***Magnaporthe oryzae*** **MoSom1.** (A) The full coding sequence of *MoSOM1* was amplified with primers SOM-E-F and SOM-Xh-R and sequenced. Six spliced isoforms of MoSom1 were found. I, MGG_04708; I2-6, amino acid sequence of MoSom1 with minor variations as indicated. (B) The protein sequence of MoSom1. The missed or extra amino acid residues in the MoSom1 isoforms were marked. Several predicted PKA phosphorylation sites (Website: http://mendel.imp.ac.at/sat/pkaPS/) were underlined.
(PPT)

Figure S9 **Sequence alignments of LUFS and ZnF_C2H2 domains from several fungal species.** (A) Sequence alignment of the LUFS (containing LisH) domain. Identical residues are shaded in black and conserved residues are shaded in gray. ScFlo8, *Saccharomyces cerevisiae* Flo8 (DAA07769); CaFlo8, *Candida albicans* Flo8 (AAQ03244); MoSom1, *Magnaporthe oryzae* MoSom1 (XP_362263). (B) Sequence alignment of the ZnF_C2H2 domain. Mo, *M. oryzae* XP_001413674; Va, *Verticillium albo-atrum* XP_003006450; Gz, *Gibberella zeae* XP_386829; Pm, *Penicillium marneffei* XP_002144056; An, *Aspergillus nidulans* XP_661814.
(PPT)

Figure S10 **qRT-PCR analysis of** ***MoSOM1*** **and** ***MoCDTF1*** **expression in several mutants.** Beta-tubulin gene (MGG_00604), *MoSOM1* and *MoCDTF1* were amplified with primer pairs of BT-F/BT-R, 145Q-F/145Q-R and 1303Q-F/1303Q-R, respectively. The Error bars represent standard deviation. Asterisks indicate a significant difference of gene expression between Guy11 and the mutants (*P*<0.01).
(TIF)

Figure S11 **Over-expression of** ***MoSOM1*** **in either** ***Δmac1*** **or** ***ΔcpkA*** **mutant is unable to overcome the defects.** (A) GFP fluorescence was observed in the nucleus for both OM1 and OC2 strains that *MoSOM1* was over-expressed in *Δmac1* and *ΔcpkA* mutants, respectively. (B) Over-expression of *MoSOM1* in *Δmac1* was unable to overcome the defect in appressorium development of the mutant. The patterns of appressorium formation between the *ΔcpkA* mutant and OC2 were also similar. Scale bar = 10 µm. (C) Like *Δmac1* and *ΔcpkA* mutants, both OM1 and OC2 strains were nonpathogenic to susceptible barley and rice.
(TIF)

Figure S12 **Localization of MoSom1 can be changed to cytoplasm and nucleus by the treatment of an adenylate cyclase inhibitor.** MDL-12,330A hydrochloride (SIGMA), an adenylate cyclase inhibitor, was dissolved in dimethyl sulfoxide (DMSO). The strain SC3 (*Δmosom1*+*MoSOM1*) was incubated in liquid CM at 25°C for 24 h, and then added MDL-12,330A to 25 and 200 µg/ml, respectively. The same concentration of DMSO (0.8%, V/V) was used as the control. The cultures were incubated for additional 48 h before photographs were taken. The GFP was still observed in nucleus for the control (top), while the fluorescence was changed to cytoplasm and nucleus for the treatments of MDL-12,330A (middle and bottom), especially for that of 200 µg/ml . BF = bright field; Scale bar = 10 µm.
(TIF)

Table S1 **Wild-type and recombinant strains of** ***Magnaporthe oryzae*** **used in this study.**
(DOC)

Table S2 **PCR primers used in this study.**
(DOC)

Table S3 **SAGE analysis of gene expression regulated by deletion of** ***MoSOM1*** **in** ***Magnaporthe oryzae*** **.**
(XLS)

Table S4 ** Several genes up- or down-regulated by deletion of** *MoSOM1* **,** ***MoRIC8*** **or** ***MoLDB1*** **in** ***Magnaporthe oryzae*** **.**
(DOC)
