# Supplementary figures and images for: Two Novel Transcriptional Regulators Are Essential for Infection-related Morphogenesis and Pathogenicity of the Rice Blast Fungus Magnaporthe oryzae
Source: PLoS Pathog. 2011 Dec 1;7(12):e1002385. doi: 10.1371/journal.ppat.1002385 (PMC3228794; doi:10.1371/journal.ppat.1002385)

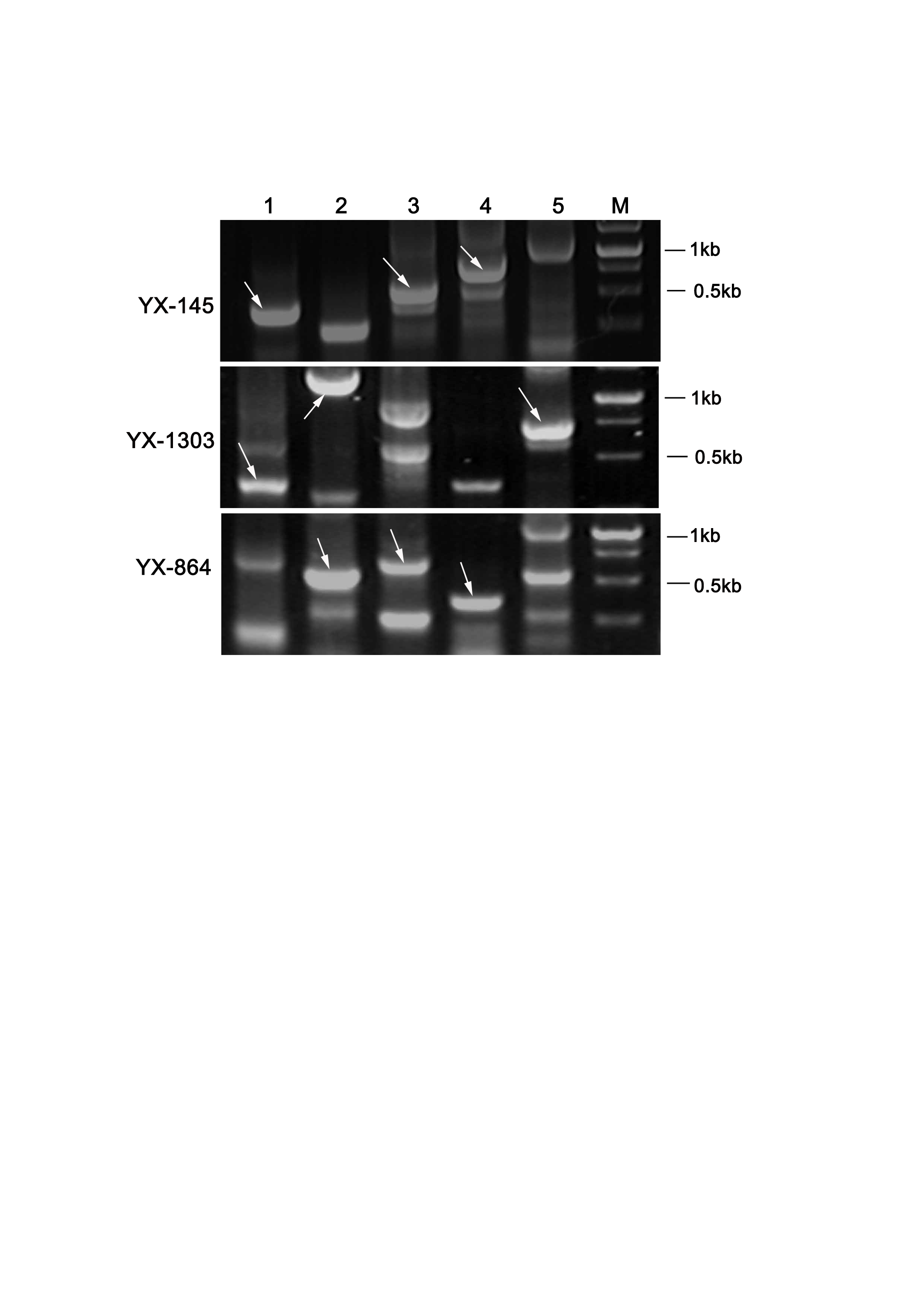

Supplement: Figure S1 — Genomic DNA flanking the integrated T-DNAs in YX-145, YX-864 and YX-1303 was amplified by hiTAIL-PCR. High-efficiency thermal asymmetric interlaced polymerase chain reaction (hiTAIL-PCR) was performed as previously described [81]. The primers used for hiTAIL-PCR were shown in Table S2. The genomic DNAs flanking right sites of the integration T-DNAs of the YX-145, YX-864 and YX-1303 mutants were obtained from the third round products, respectively. The arrows indicated the PCR products were harvested for cloning and sequencing. M, 250 bp marker (Takara). (TIF) [file ppat.1002385.s001.tif]

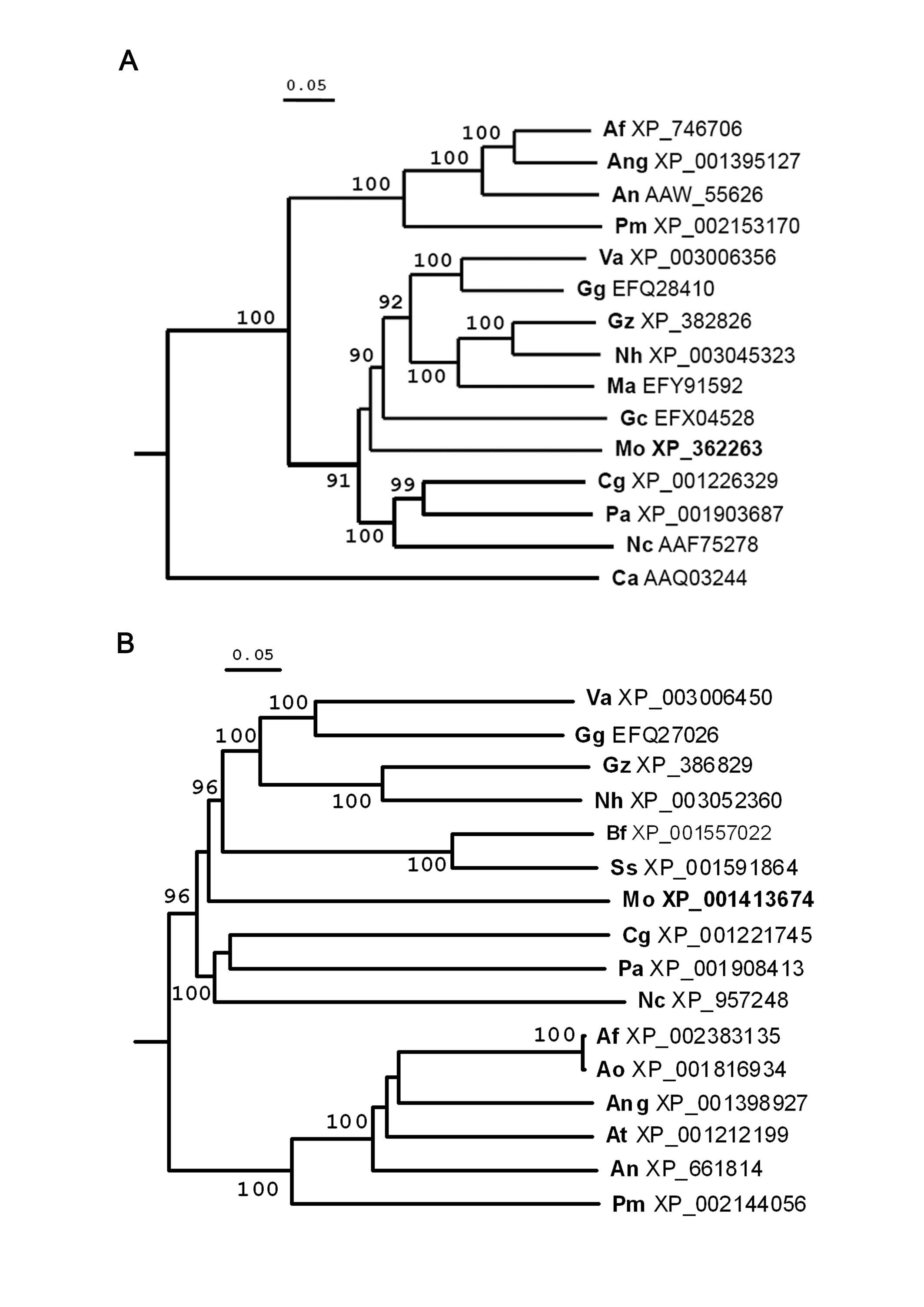

Supplement: Figure S2 — Phylogenetic analysis of Magnaporthe MoSom1 and MoCdtf1 with the homologs from other fungal species. (A) Phylogenetic tree of Magnaporthe MoSom1 and 14 homologs from other fungal species was constructed by observed divergency distance method in the program DNAMAN. Numbers at the nodes in the rooted tree represent bootstrapping value on 1000 replications. Abbreviations and numbers correspond to species names and GenBank accession numbers, respectively. Af, Aspergillus fumigatus; An, A. nidulans; Ang, A. niger; Ca, Candida albicans (Flo8); Cg, Chaetomium globosum; Gc, Grosmannia clavigera; Gg, Glomerella graminicola; Gz, Gibberella zeae; Ma, Metarhizium acridum; Mo, Magnaporthe oryzae (MoSom1); Nc, Neurospora crassa; Nh, Nectria haematococca; Pa, Podospora anserine; Pm, Penicillium marneffei; Va, Verticillium albo-atrum. (B) Phylogenetic tree of Magnaporthe MoCdtf1 and 15 homologs from other species was constructed as described above. Af, Aspergillus fumigatus; An, A. nidulans; Ang, A. niger; Ao, A. oryzae; At, A. terreus; Bf, Botryotinia fuckeliana; Cg, Chaetomium globosum; Gg, Glomerella graminicola; Gz, Gibberella zeae; Mo, Magnaporthe oryzae (MoCdtf1); Nc, Neurospora crassa; Nh, Nectria haematococca; Pa, Podospora anserine; Pm, Penicillium marneffei; Ss, Sclerotinia sclerotiorum; Va, Verticillium albo-atrum. The bar indicates 0.05 distance units. DNAMAN version 5.2.2 program was used for alignment and phylogenetic tree constrution. (TIF) [file ppat.1002385.s002.tif]

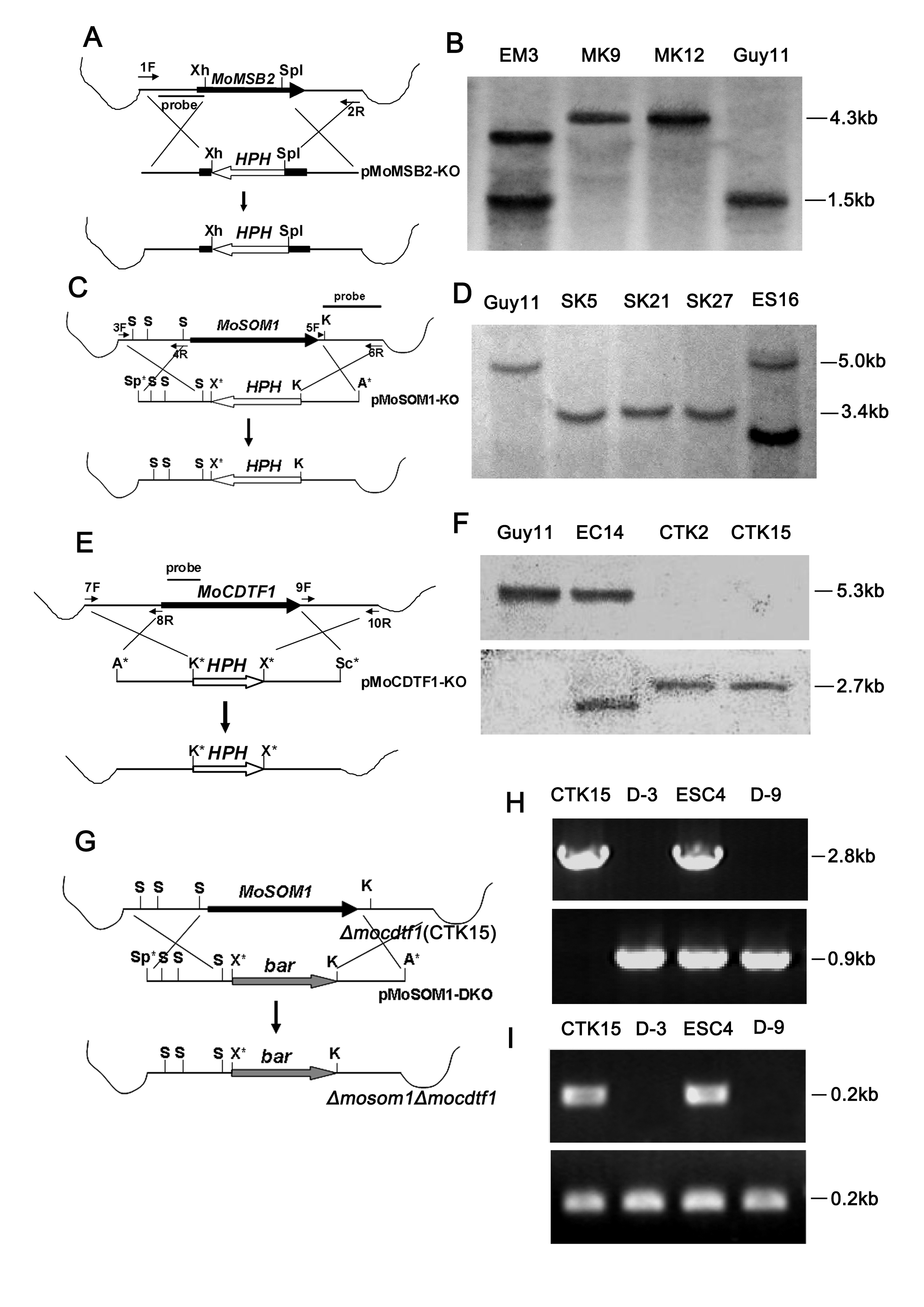

Supplement: Figure S3 — Targeted gene replacement of MoMSB2 , MoSOM1 and MoCDTF1 . (A) Construction of the vector pMoMSB2-KO and targeted gene replacement of MoMSB2. (B) Southern blot analysis. Genomic DNA was digested with HindIII/SacI and probed with a 0.9 kb fragment amplified with the primers P864-F and P864-R. Lane 1, EM3 (ectopic); lane 2 and 3, MK9 and MK12 (Δmomsb2); lane 4, Guy11. (C) Construction of the vector pMoSOM1-KO and targeted gene replacement of MoSOM1. (D) Southern blot analysis. Genomic DNA was digested with SalI and probed with a 1.2 kb fragment amplified with the primers 5F and 6R. Lane 1, Guy11; lane 2 to 4, SK5, SK21 and SK27 (Δmosom1); lane 4, ES16 (ectopic). (E) Construction of the vector pMoCDTF1-KO and targeted gene replacement of MoCDTF1. (F) Southern blot analysis. Genomic DNA was digested with BamHI and probed with a 1.0 kb fragment (top) amplified with the primers P1303-F and P1303-R and a 1.4 kb HPH cassette (bottom), respectively. Lane 1, Guy11; lane 2, EC14 (ectopic); lanes 3 and 4, CTK2 and CTK15 (Δmocdtf1). (G) Construction of the double KO vector pMoSOM1-DKO and targeted gene replacement of MoSOM1 in the Δmocdtf1 mutant (CTK15). (H) Δmosom1Δmocdtf1 mutants confirmed by PCR analysis. The MoSOM1 coding sequence were amplified with primers 145-F and 145H-Hind-R in CTK15 (Δmocdtf1) and ESC4, but absent in D-3 and D-9 (top). A bar gene cassette could be amplified with primers Bar-Xba-F and Bar-Kpn-R from all other strains, except CTK15 (bottom). Lane 1, CTK15 (Δmocdtf1); lane 2 and 4, D-3 and D-9 (Δmosom1Δmocdtf1); lanes 3, ESC4 (transformant with ectopic integration of pMoSOM1-DKO). (I) Δmosom1Δmocdtf1 mutants confirmed by RT-PCR analysis. 0.2 kb PCR products were amplified with primers 145Q-F and 145Q-R for CTK15 (Δmocdtf1) and ESC4, but absent for D-3 and D-9 (top). A 0.2 kb beta-tubulin gene (MGG_00604.6) fragment could be amplified from all the strains with primers BT-F and BT-R (bottom). A = ApaI; K = KpnI; S = SalI; Sc = SacI; Sp = SpeI; Spl = SplI; [file ppat.1002385.s003.tif]

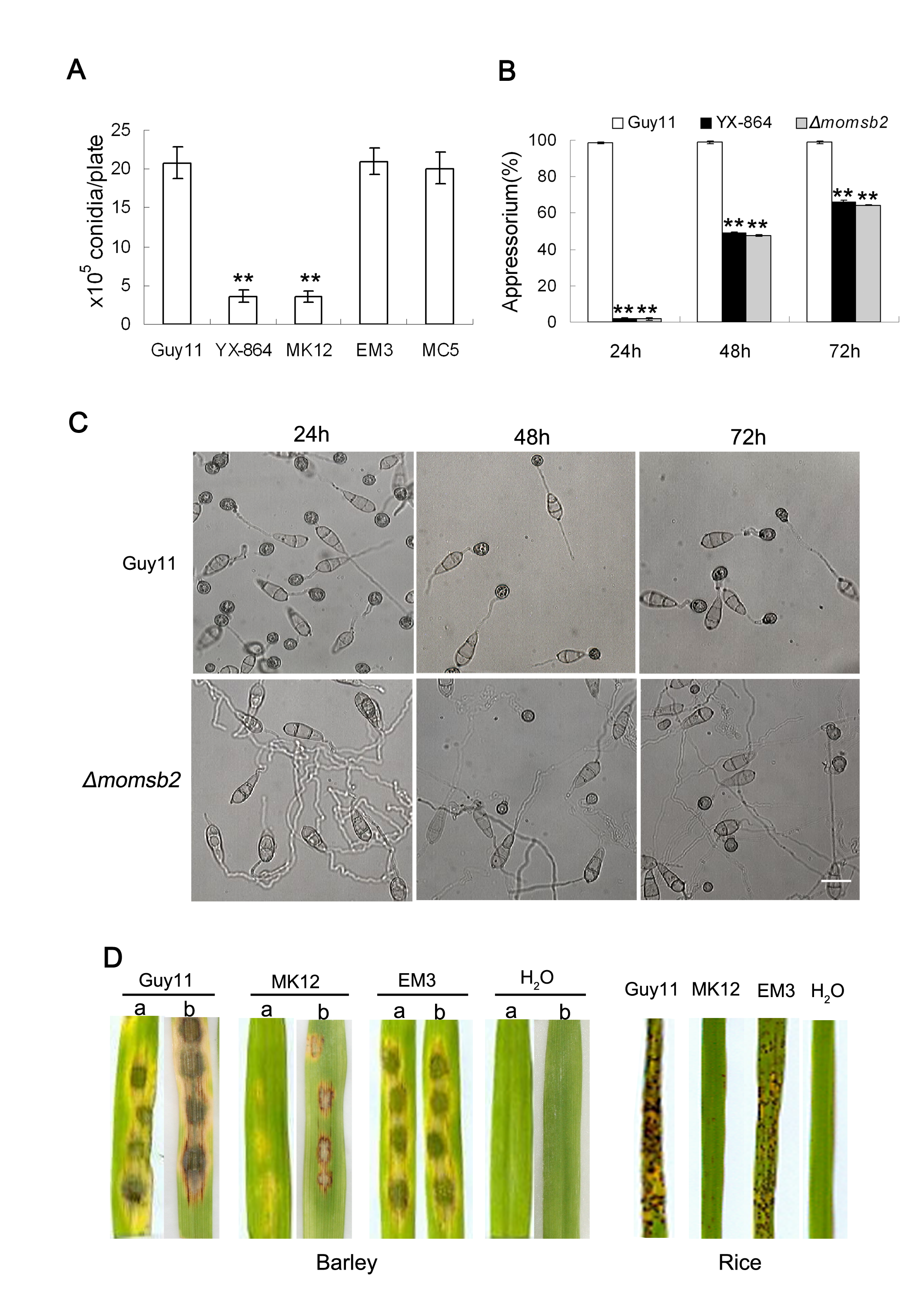

Supplement: Figure S4 — MoMSB2 is required for conidiation, appressorium formation and pathogenicity in Magnaporthe oryzae . (A) Bar chart showing the conidial production of various strains. Error bars represent the standard deviation. The YX-864 and MK12 (Δmomsb2) mutants were reduced in conidiation on CM medium. Asterisks indicate a significant difference of conidiation between Guy11 and the mutants (P<0.01). Guy11, the wild type strain; YX-864, the T-DNA insertional mutant; MK12, Δmomsb2; EM3 (ectopic); MC5 (Δmomsb2+MoMSB2). (B) Bar chart showing appressorium formation. Conidial suspension was dropped on GelBond films to allow appressorium formation and incubated at 25°C for 24, 48, and 72 h. Percentage of conidia to form appressoria was calculated under a microscope. The Δmomsb2 mutant (MK12) was delayed and reduced to form appressoria. Asterisks indicate a significant difference of appressorium formation between Guy11 and the mutants (P<0.01). (C) Guy11 formed numerous melanized appressoria at 24 h, while the MK12 mutant could hardly produce appressoria at this stage. However, the MK12 mutant could form some appressoria for 48 h and 72 h incubation. Scale bar = 10 µm. (D) Pathogenicity assays. Barley segments were inoculated with the conidial drops (10 µl per drop; 1×10^5^ conidia/ml). a = unwounded leaf and b = abraded leaf. Rice leaves were spray-inoculated with the conidia at a concentration 1×10^5^ conidia/ml. H2O containing 0.2% gelatin was used as the control. Photographs were taken at 5 days after inoculation. (TIF) [file ppat.1002385.s004.tif]

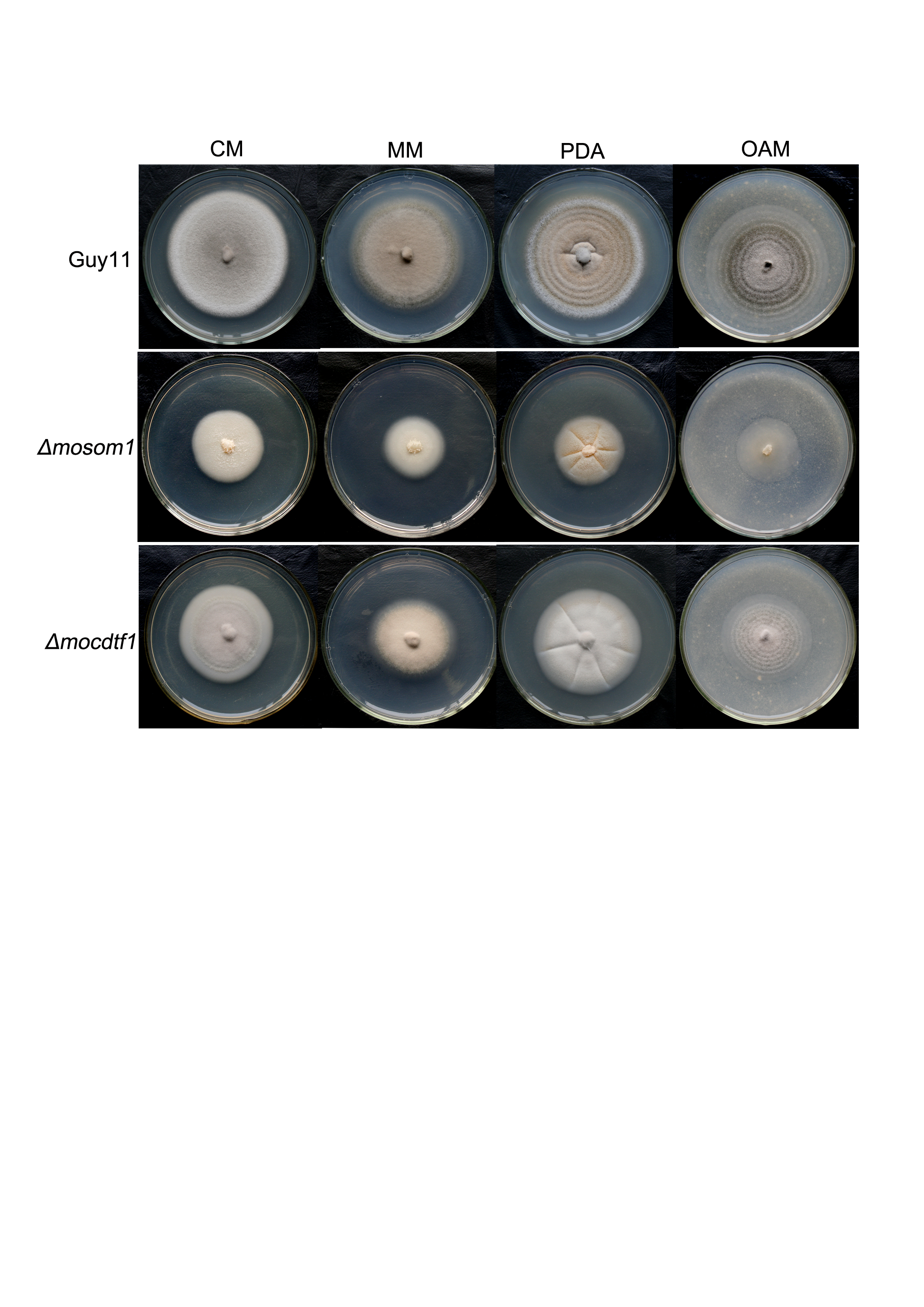

Supplement: Figure S5 — Growth patterns of the Δmosom1 and Δmocdtf1 mutants on various media. The Δmosom1 (SK27) and Δmocdtf1 (CTK15) mutants reduced in vegetative growth and mycelium pigmentation on different media, CM, MM, PDA and OAM. (TIF) [file ppat.1002385.s005.tif]

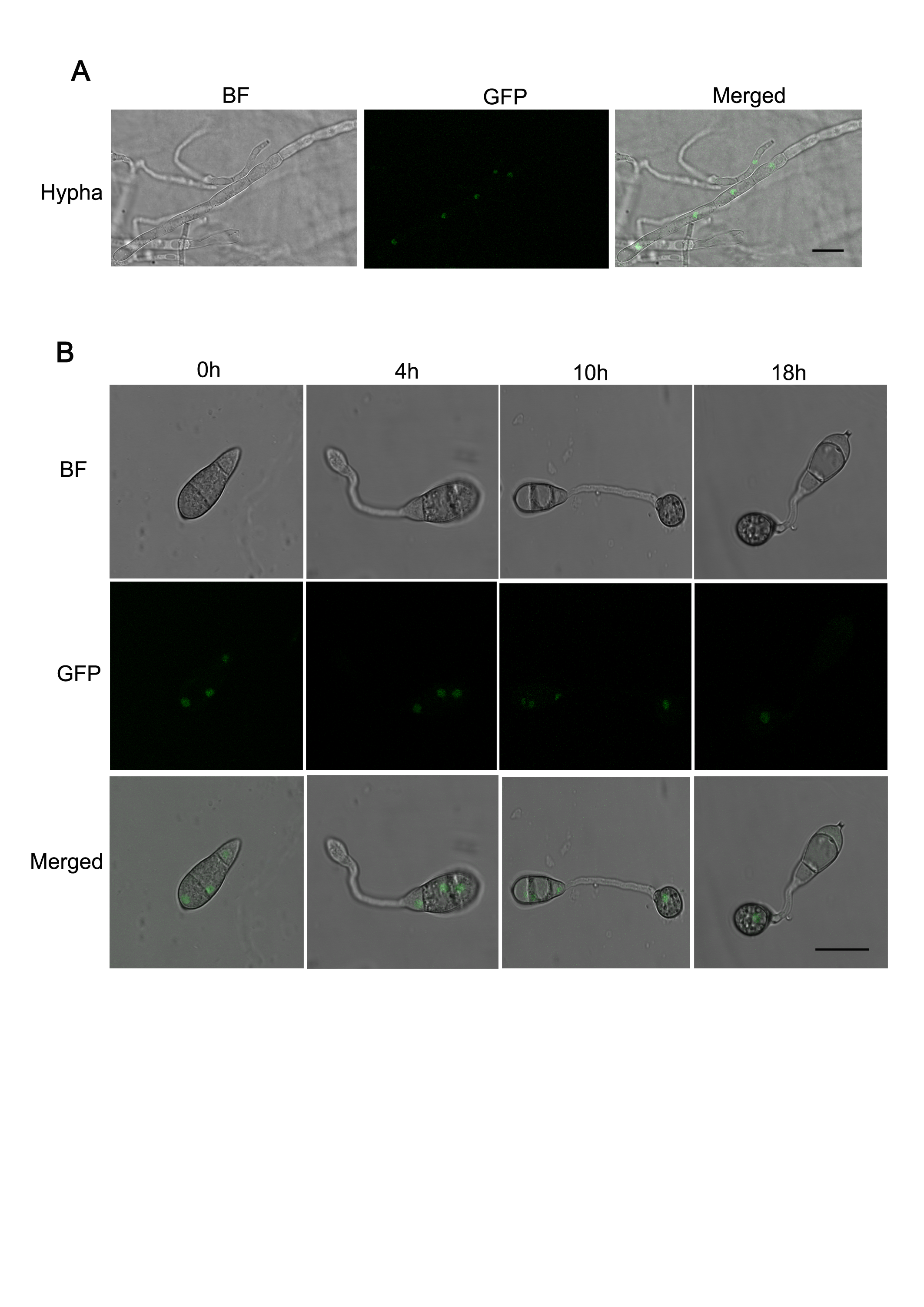

Supplement: Figure S6 — Intracellular localization of MoCdtf1-green fluorescent protein. (A) Expression of Magnaporthe oryzae MoCDTF1 in hyphae of the strain CTC1, which carries a single GFP-carboxy translational fusion of MoCDTF1. The GFP fluorescence was observed in the nucleus. (B) The patterns of MoCDTF1 expression and nuclear division during appressorium development in M. oryzae. Conidia of the strain CTC1 was allowed to germinate on hydrophobic GelBond film surfaces. Photographs were taken at various time intervals. BF = bright field. Scale bars = 10 µm. (TIF) [file ppat.1002385.s006.tif]

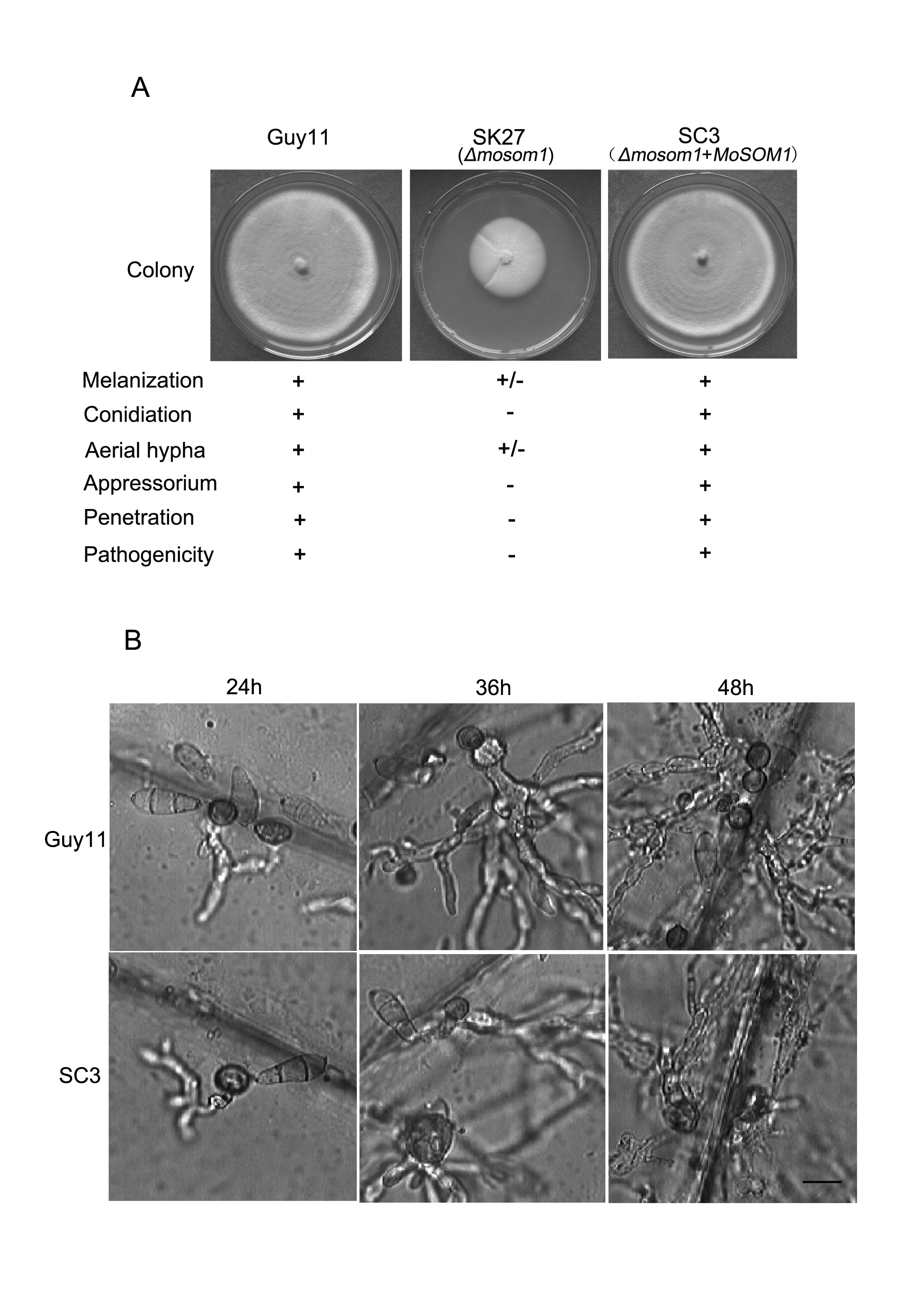

Supplement: Figure S7 — The phenotypes of Δmosom1 mutants are restored by re-introduction of MoSOM1 gene. (A) The colony morphology of the complementation transformant SC3 (Δmosom1+MoSOM1) was similar with the wild type strain Guy11. All defects of the Δmosom1 mutant (SK27), including vegetative growth, mycelium pigmented melanization, conidiation and appressorium formation, were overcome by re-introduction of MoSOM1 gene. +, normal; +/−, significantly reduced; −, not any. (B) Conidia from SC3 geminated and formed numerous appressoria on onion epidermis. Scale bar = 10 µm. (TIF) [file ppat.1002385.s007.tif]

## Slide 1
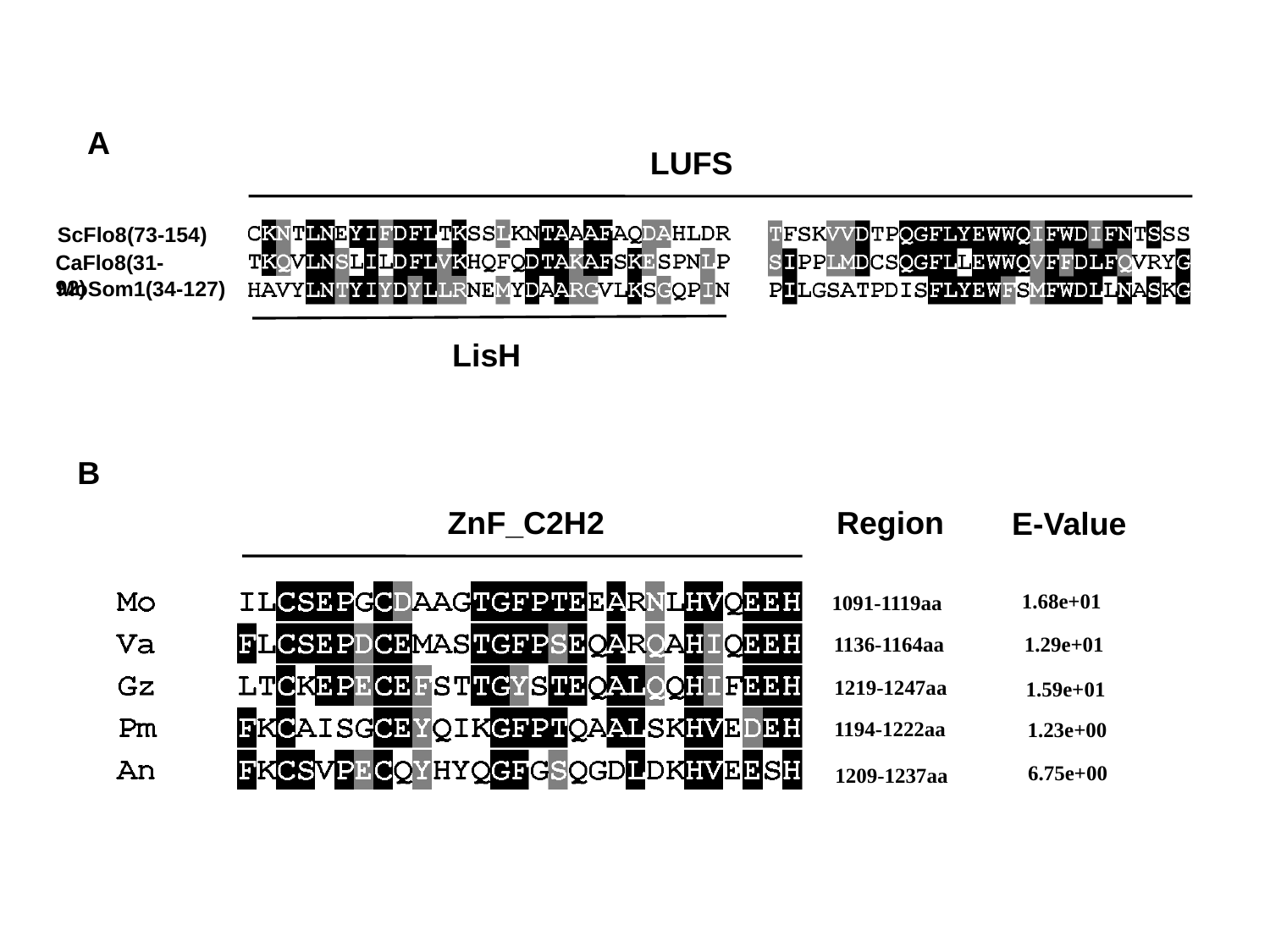

A
LUFS
ScFlo8(73-154)
CaFlo8(31-92)
MoSom1(34-127)
LisH
B
ZnF_C2H2
Region
E-Value
1.68e+01
1091-1119aa
1.29e+01
1136-1164aa
1219-1247aa
1.59e+01
1194-1222aa
1.23e+00
6.75e+00
1209-1237aa

Supplement: Figure S9 — Sequence alignments of LUFS and ZnF_C2H2 domains from several fungal species. (A) Sequence alignment of the LUFS (containing LisH) domain. Identical residues are shaded in black and conserved residues are shaded in gray. ScFlo8, Saccharomyces cerevisiae Flo8 (DAA07769); CaFlo8, Candida albicans Flo8 (AAQ03244); MoSom1, Magnaporthe oryzae MoSom1 (XP_362263). (B) Sequence alignment of the ZnF_C2H2 domain. Mo, M. oryzae XP_001413674; Va, Verticillium albo-atrum XP_003006450; Gz, Gibberella zeae XP_386829; Pm, Penicillium marneffei XP_002144056; An, Aspergillus nidulans XP_661814. (PPT) [file ppat.1002385.s009.ppt]

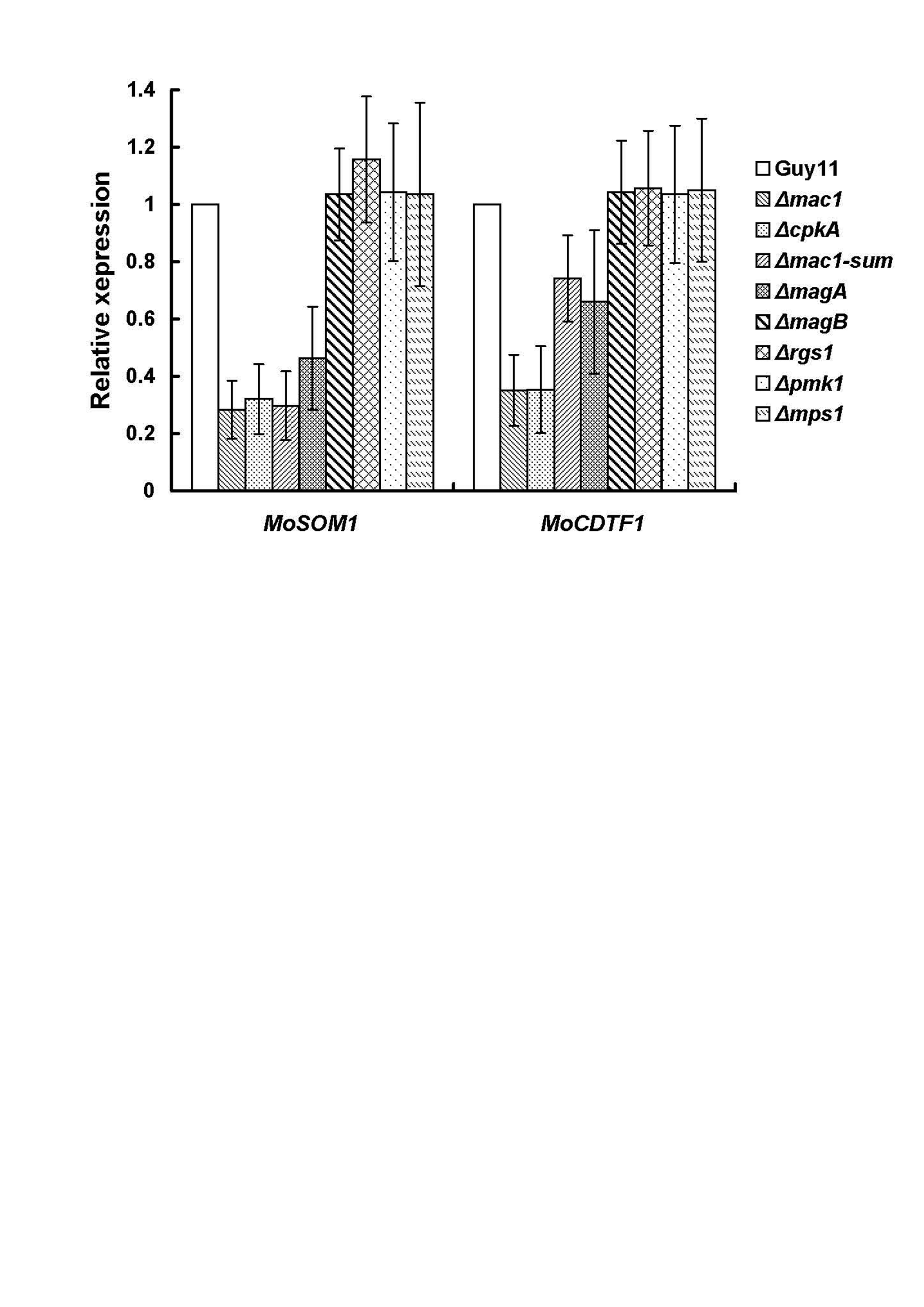

Supplement: Figure S10 — qRT-PCR analysis of MoSOM1 and MoCDTF1 expression in several mutants. Beta-tubulin gene (MGG_00604), MoSOM1 and MoCDTF1 were amplified with primer pairs of BT-F/BT-R, 145Q-F/145Q-R and 1303Q-F/1303Q-R, respectively. The Error bars represent standard deviation. Asterisks indicate a significant difference of gene expression between Guy11 and the mutants (P<0.01). (TIF) [file ppat.1002385.s010.tif]

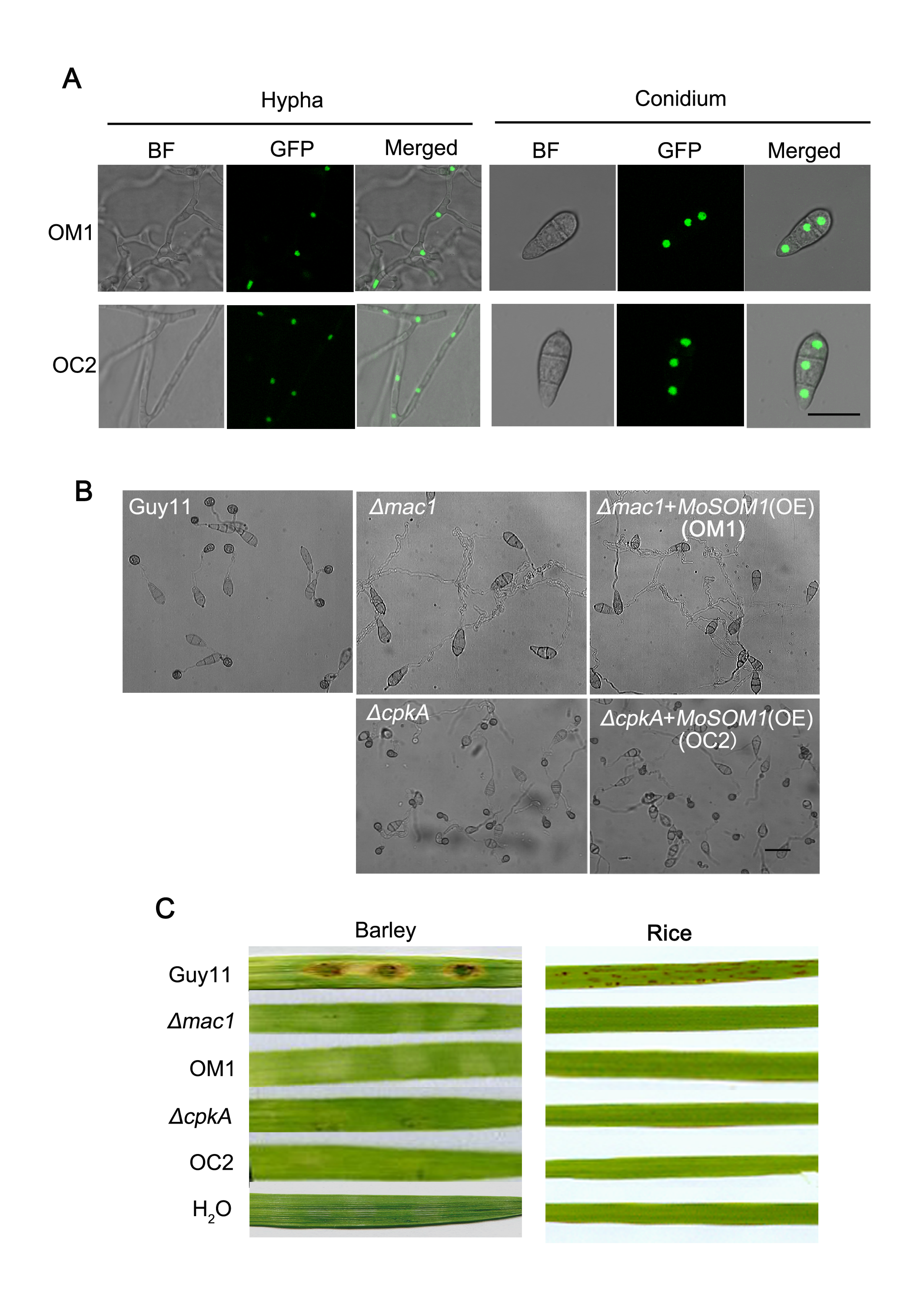

Supplement: Figure S11 — Over-expression of MoSOM1 in either Δmac1 or ΔcpkA mutant is unable to overcome the defects. (A) GFP fluorescence was observed in the nucleus for both OM1 and OC2 strains that MoSOM1 was over-expressed in Δmac1 and ΔcpkA mutants, respectively. (B) Over-expression of MoSOM1 in Δmac1 was unable to overcome the defect in appressorium development of the mutant. The patterns of appressorium formation between the ΔcpkA mutant and OC2 were also similar. Scale bar = 10 µm. (C) Like Δmac1 and ΔcpkA mutants, both OM1 and OC2 strains were nonpathogenic to susceptible barley and rice. (TIF) [file ppat.1002385.s011.tif]

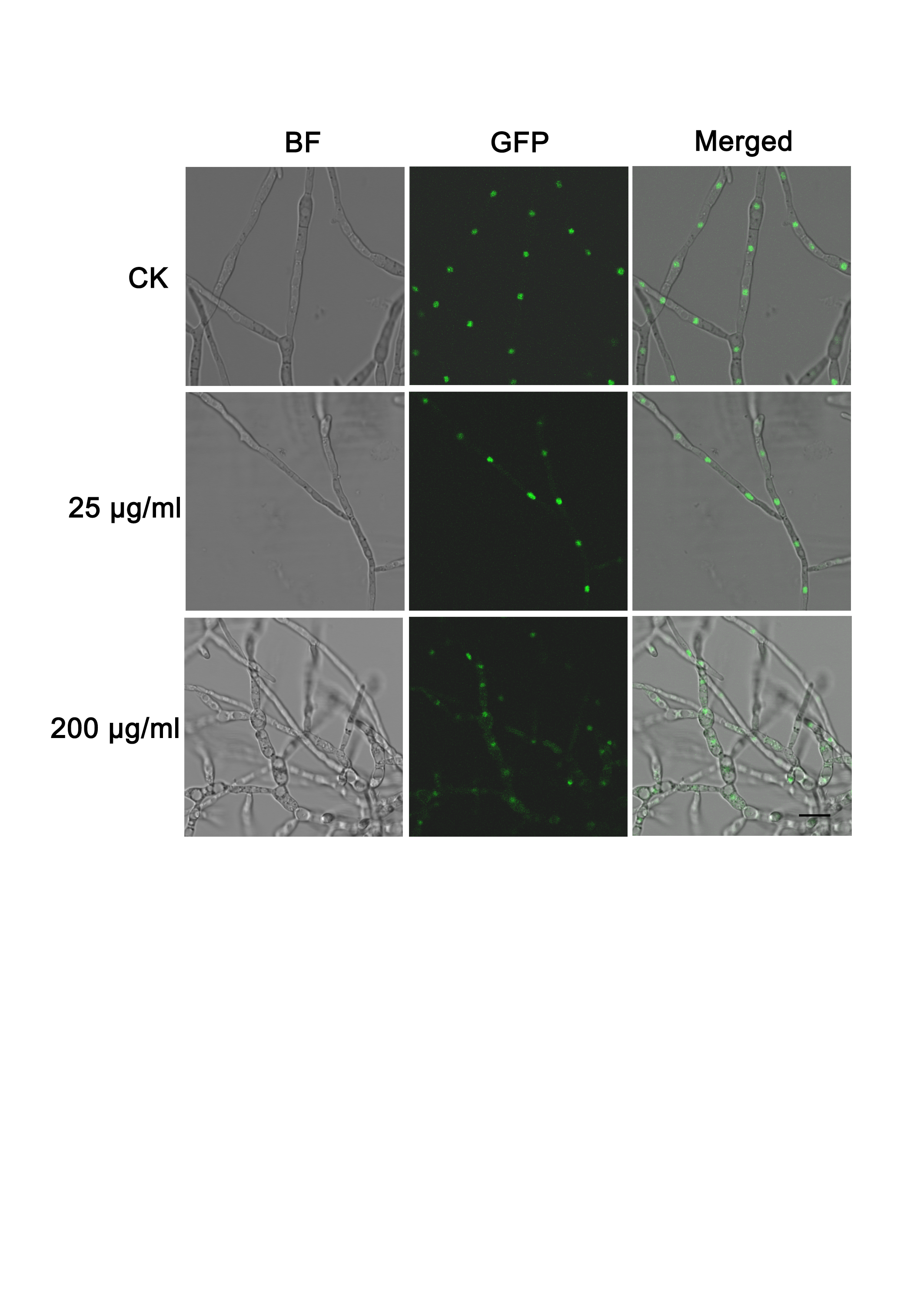

Supplement: Figure S12 — Localization of MoSom1 can be changed to cytoplasm and nucleus by the treatment of an adenylate cyclase inhibitor. MDL-12,330A hydrochloride (SIGMA), an adenylate cyclase inhibitor, was dissolved in dimethyl sulfoxide (DMSO). The strain SC3 (Δmosom1+MoSOM1) was incubated in liquid CM at 25°C for 24 h, and then added MDL-12,330A to 25 and 200 µg/ml, respectively. The same concentration of DMSO (0.8%, V/V) was used as the control. The cultures were incubated for additional 48 h before photographs were taken. The GFP was still observed in nucleus for the control (top), while the fluorescence was changed to cytoplasm and nucleus for the treatments of MDL-12,330A (middle and bottom), especially for that of 200 µg/ml . BF = bright field; Scale bar = 10 µm. (TIF) [file ppat.1002385.s012.tif]
