# Supplementary material for: Two Novel Transcriptional Regulators Are Essential for Infection-related Morphogenesis and Pathogenicity of the Rice Blast Fungus Magnaporthe oryzae
Source: PLoS Pathog. 2011 Dec 1;7(12):e1002385. doi: 10.1371/journal.ppat.1002385 (PMC3228794; doi:10.1371/journal.ppat.1002385)
Supplement: Figure S8 — Putative spliced isoforms of Magnaporthe oryzae MoSom1. (A) The full coding sequence of MoSOM1 was amplified with primers SOM-E-F and SOM-Xh-R and sequenced. Six spliced isoforms of MoSom1 were found. I, MGG_04708; I2-6, amino acid sequence of MoSom1 with minor variations as indicated. (B) The protein sequence of MoSom1. The missed or extra amino acid residues in the MoSom1 isoforms were marked. Several predicted PKA phosphorylation sites (Website: http://mendel.imp.ac.at/sat/pkaPS/) were underlined. (PPT) [file ppat.1002385.s008.ppt]

## Slide 1
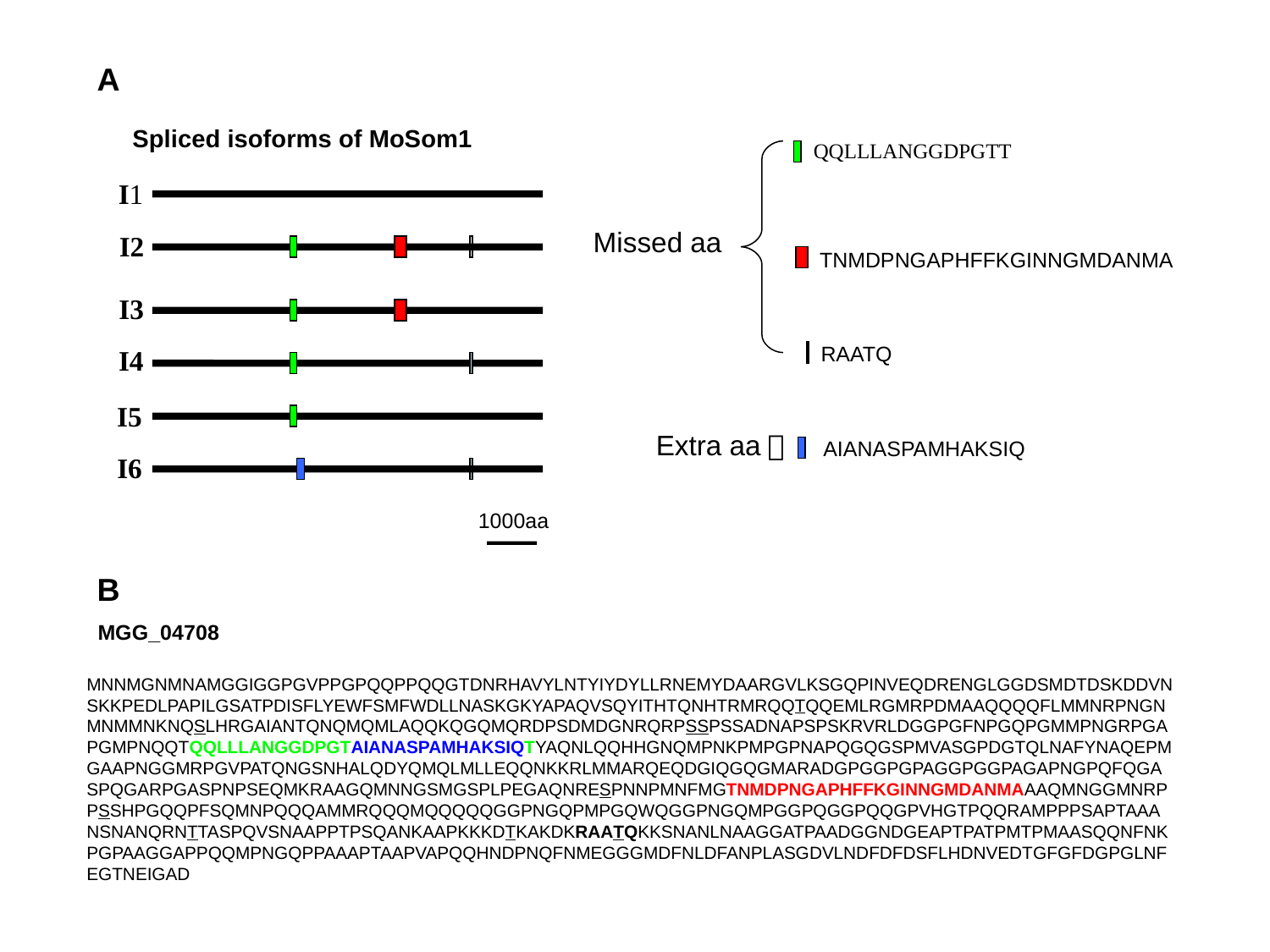

A
Spliced isoforms of MoSom1
QQLLLANGGDPGTT
I1
Missed aa
I2
TNMDPNGAPHFFKGINNGMDANMA
I3
RAATQ
I4
I5
Extra aa：
AIANASPAMHAKSIQ
I6
1000aa
B
MGG_04708
MNNMGNMNAMGGIGGPGVPPGPQQPPQQGTDNRHAVYLNTYIYDYLLRNEMYDAARGVLKSGQPINVEQDRENGLGGDSMDTDSKDDVNSKKPEDLPAPILGSATPDISFLYEWFSMFWDLLNASKGKYAPAQVSQYITHTQNHTRMRQQTQQEMLRGMRPDMAAQQQQFLMMNRPNGNMNMMNKNQSLHRGAIANTQNQMQMLAQQKQGQMQRDPSDMDGNRQRPSSPSSADNAPSPSKRVRLDGGPGFNPGQPGMMPNGRPGAPGMPNQQTQQLLLANGGDPGTAIANASPAMHAKSIQTYAQNLQQHHGNQMPNKPMPGPNAPQGQGSPMVASGPDGTQLNAFYNAQEPMGAAPNGGMRPGVPATQNGSNHALQDYQMQLMLLEQQNKKRLMMARQEQDGIQGQGMARADGPGGPGPAGGPGGPAGAPNGPQFQGASPQGARPGASPNPSEQMKRAAGQMNNGSMGSPLPEGAQNRESPNNPMNFMGTNMDPNGAPHFFKGINNGMDANMAAAQMNGGMNRPPSSHPGQQPFSQMNPQQQAMMRQQQMQQQQQGGPNGQPMPGQWQGGPNGQMPGGPQGGPQQGPVHGTPQQRAMPPPSAPTAAANSNANQRNTTASPQVSNAAPPTPSQANKAAPKKKDTKAKDKRAATQKKSNANLNAAGGATPAADGGNDGEAPTPATPMTPMAASQQNFNKPGPAAGGAPPQQMPNGQPPAAAPTAAPVAPQQHNDPNQFNMEGGGMDFNLDFANPLASGDVLNDFDFDSFLHDNVEDTGFGFDGPGLNFEGTNEIGAD
