# Supplementary material for: Two Novel Transcriptional Regulators Are Essential for Infection-related Morphogenesis and Pathogenicity of the Rice Blast Fungus Magnaporthe oryzae
Source: PLoS Pathog. 2011 Dec 1;7(12):e1002385. doi: 10.1371/journal.ppat.1002385 (PMC3228794; doi:10.1371/journal.ppat.1002385)
Supplement: Table S1 — Wild-type and recombinant strains of Magnaporthe oryzae used in this study. (DOC) [file ppat.1002385.s013.doc]

**Table S1. Wild-type and recombinant strains of *Magnaporthe oryzae* used in this study.**

| **Strain** | **Brief description** | **Reference** |
| --- | --- | --- |
| Guy11 | Wild-type, MAT1-2 | [74] |
| TH3 | Wild-type, MAT1-1 | [56] |
| *ΔmagB* | *MAGB* gene disruption mutant of 70-15 | [50] |
| *Δpmk1* | *Δpmk1* mutant of Guy11 | [40] |
| *ΔcpkA* | *ΔcpkA* mutant of 4091-5-8 | [14] |
| *Δmps1* | *Δmps1* mutant of Guy11 | [41] |
| *Δmac1_sum_* | *Δmac1_sum_* mutant of Guy11 | [44] |
| *Δ rgs1* | *Δ rgs1* mutant of B157 | [52] |
| MYX8, MYX13 | *Δmac1* mutants of Guy11 | This study |
| MA6, MA21 | *ΔmagA* mutants of Guy11 | This study |
| YX-145 | T-DNA insertional mutant | This study |
| YX-864 | T-DNA insertional mutant | This study |
| YX-1303 | T-DNA insertional mutant | This study |
| SK5, SK21, SK27 | *Δmosom1* mutants of Guy11 | This study |
| ES16 | Ectopic transformant of Guy11 with integration of pMoSOM1-KO | This study |
| SC1,SC3 | *Δmosom1* mutant (SK27) transformed with pMoSOM1-GFP | This study |
| MK9, MK12 | *Δmomsb2* mutants of Guy11 | This study |
| EM3 | Ectopic transformant of Guy11 with integration of pMoMSB2-KO | This study |
| MC1, MC5 | *Δmomsb2* mutant (MK12) transformed with pMoMSB2-HB | This study |
| CTK2,CTK15 | *Δmocdtf1* mutants of Guy11 | This study |

| EC14 | Ectopic transformant of Guy11 with integration of pMoCDTF1-KO | This study |
| --- | --- | --- |
| CTC1, CTC5 | *Δmocdtf1* mutant (CTK15) transformed with pMoCDTF1-GFP | This study |
| D-3, D-9 | Double knock-out strains *Δmosom1Δmocdtf1* | This study |
| ESC4 | CTK15 with integration of pMoSOM1-DKO | This study |
| SN1-2, SN1-5 | Transformants expressing *MoSOM1*^ΔPKKK^-GFP | This study |
| SN2-3, SN2-4 | Transformants expressing  *MoSOM1*^ΔPSKRVRL^-GFP | This study |
| SL1, SL7 | Transformants expressing *MoSOM1*^ΔLISH^-GFP | This study |
| CN-2, CN-6 | Transformants expressing MoCDTF^ΔPPKRKKP^-GFP | This study |
| OC2, OC7 | Overexpession of *MoSOM1* in *ΔcpkA* | This study |
| OM1, OM4 | Overexpession of *MoSOM1* in *Δmac1* | This study |
