# Supplementary material for: Two Novel Transcriptional Regulators Are Essential for Infection-related Morphogenesis and Pathogenicity of the Rice Blast Fungus Magnaporthe oryzae
Source: PLoS Pathog. 2011 Dec 1;7(12):e1002385. doi: 10.1371/journal.ppat.1002385 (PMC3228794; doi:10.1371/journal.ppat.1002385)
Supplement: Table S2 — PCR primers used in this study. (DOC) [file ppat.1002385.s014.doc]

**Table S2. PCR primers used in this study.**

| **Name** | **Sequence (5’→3’)*** |
| --- | --- |
| 1F | TTCACTTCCTTTCCTTCGCT |
| 2R | CGCTTGACATCTCGATCAC |
| HPH-Spl-F | CGTACGTATTGAAGGAGCATTTTTGG |
| HPH-Xho-F | CTCGAGTTCTCGAAATCAGCTCTTGT |
| 864H-Sal-F | ACGCGTCGACAAGAATGAGCGGATCCAG |
| 864H-Spe-R | GGACTAGTGTTCCAGCCGAGTGAGTTCT |
| GFP-Spe-F | ACTAGTATGGTGAGCAAGGGCGAGGA |
| GFP-Xho-R | CCGCTCGAGGTGGAGATGTGGAGTG |
| 864Q-F | CGAGGCATATCAAGAGACA |
| 864Q-R | CGAGTCGGTAGTCAATGCAT |
| P864-F | TCAATTCCTGGGCCGAAGAC |
| P864-R | CAGTTCCCTCATGAGACCC |
| 3F | GTAACAAAGTCGGCGCATCG |
| 4R | TCTAGATGTCTGCGCAGTTTGATT |
| 5F | GATTTCGTATCGTTCGTAC |
| 6R | GTCATTTGTAGTTCGTGGGT |
| HPH-Kpn-F | GGTACCTATTGAAGGAGCATTTTTGG |
| HPH-Xba-F | TCTAGATTCTCGAAATCAGCTCTTGT |
| Bar-Xba-F | GCTCTAGAAAGATGATATTGAAGGAG |
| Bar-kpnI-F | GCGGTACCAGAAGATGATATTGAAGG |
| 145-F | ATGAACAACATGGGGAATAT |
| 145-R | AGCCCATTCTCGCGATCTT |
| 145Q-F | CATTACACATACGCAGAACC |
| 145Q-R | CGAGCATCTGCATTTGGTTC |
| BT-F | ATTGGTGCTGCTTTCTGGCA |
| BT-R | GGAAGAGCTGGCCAAAAGGA |
| 145H-Nde-F | GGAATTCCATATGCTACAACATGCTCCGTGG |
| 145H-Hind-R | CCCAAGCTTGTCGGCGCCAATTTCGTT |
| GFP-Hind-F | AAGCTTATGGTGAGCAAGGGCGAGGA |
| GFP-Xho-R | CCGCTCGAGGTGGAGATGTGGAGTGGGCG |
| PKKK-R | TTTAGCTTTGGTATCGGCTGCCTTGTTAGCTTG |
| PKKK-F | CAAGCTAACAAGGCAGCCGATACCAAAGCTAAA |
| PSK-F | GGGGTACCGACGGCGGCCCTGGGTTCAA |
| PSK-R | GGGGTACCTGATGGAGCATTGTCAGCTG |
| LisH-F | GCGATCTTGTTCAACGCGATTGTCGGTGCCT |
| LisH-R | AGGCACCGACAATCGCGTTGAACAAGATCGC |
| 7F | TAAATCCTTTGGTCAACCTC |
| 8R | GGTACCTTTAGACTGTATGAGTTGC |
| 9F | TCTAGACGGAAGGCATGGATACTT |
| 10R | TTGTTATCGAAAAGCCAGATT |
| P1303-F | ATGGCCGAATTACAGAAC |
| P1303-R | TGGCCGGGCTCTGAGATG |
| 1303H-Aat-F | CGCGACGTCTCTCGAGGTATTCAAGGC |
| 1303H-Kpn-R | GGGGTACCGTTCGCTTCCAAGATGTA |
| GFP-Kpn-F | GGTACCATGGTGAGCAAGGGCGAG |
| 145OE-Xho-F | CCGCTCGAGATGAACAACATGGGGAATAT |
| 1303Q-F | CCTGTTCATCCGCTACCGAC |
| 1303Q-R | GTTGTTGAGCTACCGGACCG |
| 1303CD-R | AGACTTGGCCAGGGTTAGGTGGGTTGAGGGT |
| 1303CD-F | ACCCTCAACCCACCTAACCCTGGCCAAGTCT |
| MPG1-RT-F | GAAGGTCGTCTCTTGCTGCA |
| MPG1-RT-R | GGATGTTGACCAGACCAATC |
| MHP1-RT-F | CACCATCATCGCCACCATC |
| MHP1-RT-R | CAGCACTGAGCAGAGCCGTA |
| 10105-RT-F | CGGCAGCGGAGACTATGA |
| 10105-RT-R | CGCAAATGTCGGTGAAGC |
| 9134-RT-F | GCAGCGGAGCCTACAACAA |
| 9134-RT-R | TCCAAGAACAGGGAGCAGACA |
| 1Q RT-07877-F | TGCCCAATCCGTCAGGAGAA |
| 1QRT-07877-R | CCATCCTCTTCAGCATTGGT |
| 2QRT-12981-F | TTCCGTCACATCACCGGCAA |
| 2QRT-12981-R | TGTAGTGGAAGGGAGGCTCT |
| 3QRT-04432-F | GTGTTGCCTTCGTCAGCTTC |
| 3QRT-04432-R | GTAAAGATGTTGCCGTACTTC |
| 4QRT-04258-R | GGCAAATACGAGTGGGCGGT |
| 4QRT-04258-F | CTATGCCACTGGCCACCACT |
| 5QRT-13654-F | ATGAAGTTCCTCGGCCTCAC |
| 5QRT-13654-R | GAGCCAATGTTCCGGACGTA |
| 6QRT-12983-F | ATGGGTCCCGGAAAGGAAAA |
| 6QRT-12983-R | CTCCAACGTGCTCCTGAAAG |
| 7QRT-01818-F(MAGA) | GATTCTACTGCTTGGTTCTG |
| 7QRT-01818-R(MAGA) | GCAGATAGTCGCAAAGTTCT |
| 8QRT-06530-F | AAGAAGATCAACCTCGGTGT |
| 8QRT-06530-R | TGGACTGGGTGATGGCAGT |
| 9QRT-15157-F | ATGCCTCTCCAACTACGTCC |
| 9QRT-15157-R | CATGACTACGGCGGTGTGA |
| 10QRT-02531-F | ATGCGCAGCACAATCCTCT |
| 10QRT-02531-R | CTTGATGCCGTCGGTGGAT |
| MAC-KO-FP | CGGGTTCGCTCAAGAAAACT |
| MAC-KO-RP | CAAGACTGCTGCACAGCATT |
| HPH-Hind-F | CCCAAGCTTTATTGAAGGAGCATTTTTGG |
| HPH-Hind-R | CCCAAGCTTTTCTCGAAATCAGCTCTTGT |
| SOM-E-F | CCGGAATTCATGAACAACATGGGGAATA |
| SOM-Xh-R | CCGCTCGAGCTAGTCGGCGCCAATTTC |
| STU-E-F | CCGGAATTCATGATCAACGGCACTAAG |
| STU-E-R | CCGGAATTCCTATCTGCGTCTGGACAC |
| LDB-E-F | GAATTCATGAGCATGGGACCTTCG |
| LDB-S-R | GTCGACTCATGACTGAGCTTGGG |
| CPK-F | ACGCGTCGACATGCCTTCTCTAGGTTTT |
| CPK-R | ACGCGTCGACTCAGAATCCAGGGAACAA |
| QRT-01230 | TACTCAAGTACCCAAGCTCA |
| QRT-01230 | ATCTGTAGGTCTTGAATGCA |
| QRT-01662 | ACGTGCTGCTCGATGTCTAT |
| QRT-01662 | AGTGAAGACCTGGTCGAG |
| QRT-02252 | ATTGTCAACTACGCCAACAG |
| QRT-02252 | GATGGTAAAGACACGGTCAA |
| QRT-02731 | TACGACCAGGCACTTATCTG |
| QRT-02731 | ACAGAATGGTGCACTTTGAC |
| QRT-03860 | TAGGGGACTCGAAGAAGAAC |
| QRT-03860 | CGTGCGTAATCATATGTGTG |
| QRT-03977 | CAACTCTCCAAACTCGCTTA |
| QRT-03977 | GGTAGTTGTATGTGGGCTGA |
| QRT-05344 | CAACATCCTCTCCATCTTCA |
| QRT-05344 | CATCGACACCTCCAATGTA |
| QRT-06368 | CTACACGCCAAAAACATCAT |
| QRT-06368 | CAGACTTGTTGTAGCCCTTG |
| QRT-09898 | AGACTTACCAGGGAGCAAAC |
| QRT-09898 | ATACACCACCTCTGGGTTCT |
| QRT-14008 | AATACTTTGTTCCCGGAGAG |
| QRT-14008 | CTTCGTCGGTAGGTAGGAGT |
| 1303YTH-E-F | CCGGAATTCATGGCCGAATTACAGAACAA |
| 1303YTH-E-R | CCGGAATTCTCAGTTCGCTTCCAAGATGT |
| LAD1 | ACGATGGACTCCAGAGCGGCCGCVNVNNNGGAA |
| LAD2 | ACGATGGACTCCAGAGCGGCCGCBNBNNNGGTT |
| LAD3 | ACGATGGACTCCAGAGCGGCCGCHNVNNNCCAC |
| LAD4 | ACGATGGACTCCAGAGCGGCCGCVVNVNNNCCAA |
| LAD5 | ACGATGGACTCCAGAGCGGCCGCBDNBNNNCGGT |
| AC1 | ACGATGGACTCCAGAG |
| R1 | CGTGACTGGGAAAACCCTGGCGTT |
| R2 | ACGATGGACTCCAGAGCGACCCAACTTAATCGCCTTGCAGCACATC |
| R3 | GAAGAGGCCCGCACCGATCGCCCTT |

*Introduced restriction sites are underlined. *Aat*II=GACGTC; *Bam*HI=GGATCC; *Eco*RI=GAATTC; *Hin*dIII=AAGCTT; *Spl*I=CGTACG; *Kpn*I=GGTACC; *Nde*I=CATATG; *Sal*I=GTCGAC; *Spe*I=ACTAGT; *Xba*I=TCTAGA; *Xho*I=CTCGAG. N=A/G/C/T; V=A/C/G; B=G/C/T; H=A/C/T; D=A/G/T
