# Supplementary material for: Two Novel Transcriptional Regulators Are Essential for Infection-related Morphogenesis and Pathogenicity of the Rice Blast Fungus Magnaporthe oryzae
Source: PLoS Pathog. 2011 Dec 1;7(12):e1002385. doi: 10.1371/journal.ppat.1002385 (PMC3228794; doi:10.1371/journal.ppat.1002385)
Supplement: Table S4 — Several genes up- or down-regulated by deletion of MoSOM1 , MoRIC8 or MoLDB1 in Magnaporthe oryzae . (DOC) [file ppat.1002385.s016.doc]

**Table S4. Several genes up- or down-regulated by deletion of *MoSOM1*, *MoRIC8* or *MoLDB1* in *Magnaporthe oryzae.***

| **Gene ID** | **log2 Ratio (*Δmosom1*/Guy11)** | **log2 Ratio (*Δmoric8*/Guy11)** | **log2 Ratio (*Δmoldb1*/Guy11)** | **Description** |
| --- | --- | --- | --- | --- |
| MGG_02246 | 11.92 | 8.78 | 8.70 | conserved hypothetical protein (633 nt) |
| MGG_03545 | 9.67 | 10.69 | 9.43 | conserved hypothetical protein (2367 nt) |
| MGG_03595 | 9.32 | 9.48 | 9.43 | G2/mitotic-specific cyclin-B1 (1293 nt) |
| MGG_06117 | 9.25 | 8.37 | 8.38 | conserved hypothetical protein (4081 nt) |
| MGG_08138 | 9.25 | 10.78 | 9.49 | glutamyl-tRNA synthetase (771 nt) |
| MGG_14904 | 8.86 | 9.82 | 9.38 | histidinol-phosphate aminotransferase (1322 nt) |
| MGG_12574 | 8.86 | 9.24 | 8.96 | aspartyl-tRNA synthetase (1801 nt) |
| MGG_08694 | 8.77 | 10.4 | 8.70 | conserved hypothetical protein (1332 nt) |
| MGG_03615 | 8.56 | 9.17 | 8.60 | conserved hypothetical protein (728 nt) |
| MGG_02101 | 8.32 | 8.69 | 9.92 | conserved hypothetical protein (1259 nt) |
| MGG_14307 | 8.12 | 6.09 | 4.56 | L-ascorbate oxidase (2029 nt) |
| MGG_11021 | 7.94 | 5.26 | 5.56 | conserved hypothetical protein (2061 nt) |
| MGG_13993 | 7.56 | 3.98 | 3.42 | conserved hypothetical protein (1147 nt) |
| MGG_15267 | 6.51 | 4.24 | 2.86 | hypothetical protein (539 nt) |
| MGG_10327 | 6.06 | 3.18 | 3.97 | conserved hypothetical protein (2544 nt) |
| MGG_11754 | 5.30 | 3.78 | 2.95 | heavy metal tolerance protein (2188 nt) |
| MGG_04240 | 5.21 | 3.49 | 3.36 | monooxygenase FAD-binding (1438 nt) |
| MGG_05128 | 5.12 | 1.46 | 3.75 | dicarboxylic amino acid permease (2339 nt) |
| MGG_05994 | 4.88 | 3.54 | 2.21 | predicted protein (4038 nt) |
| MGG_08440 | 4.86 | 3.76 | 4.26 | trichothecene 3-O-acetyltransferase (1428 nt) |
| MGG_02781 | 4.53 | 3.35 | 3.56 | conserved hypothetical protein (4354 nt) |
| MGG_12580 | 4.47 | 2.36 | 2.98 | conserved hypothetical protein (3191 nt) |
| MGG_12230 | 4.40 | 3.45 | 3.01 | hypothetical protein (675 nt) |
| MGG_06096 | 4.38 | 2.42 | 1.59 | tRNA wybutosine-synthesizing protein 1 (2445 nt) |
| MGG_05980 | 4.35 | 2.83 | 2.30 | pyridoxine biosynthesis protein PDX1 (1308 nt) |
| MGG_00370 | 4.24 | 2.92 | 3.11 | pre-mRNA-splicing factor cwc-21 (800 nt) |
| MGG_00045 | 4.23 | 3.67 | 3.84 | general alpha-glucoside permease (2325 nt) |
| MGG_11682 | 4.15 | 2.82 | 2.23 | phenylacetone monooxygenase (1824 nt) |
| MGG_01391 | 4.03 | 2.38 | 3.38 | ent-kaurene oxidase (2562 nt) |
| MGG_04595 | 3.93 | 4.37 | 2.93 | hypothetical protein (830 nt) |
| MGG_11427 | 3.86 | 5.41 | 4.05 | intracellular protein transport protein (UsoA) (3227 nt) |
| MGG_04876 | 3.82 | 1.94 | 2.71 | succinate dehydrogenase cytochrome B subunit (899 nt) |
| MGG_04900 | 3.80 | 3.2 | 4.84 | alkaline phosphatase (2308 nt) |
| MGG_01990 | 3.77 | 2.08 | 3.05 | b-ZIP transcription factor IDI-4 (1005 nt) |
| MGG_03375 | 3.74 | 1.05 | 2.39 | isotrichodermin C-15 hydroxylase (2078 nt) |
| MGG_06298 | 3.68 | 1.92 | 3.17 | hypothetical protein (1526 nt) |
| MGG_00658 | 3.68 | 3.68 | 2.41 | conserved hypothetical protein (828 nt) |
| MGG_04652 | 3.65 | 2.55 | 2.08 | DNA-directed RNA polymerase II subunit RPB1 (5874 nt) |
| MGG_01756 | 3.58 | 2.42 | 2.08 | ubiquitin-conjugating enzyme (1092 nt) |
| MGG_13875 | 3.53 | 4.36 | 2.61 | conserved hypothetical protein (1041 nt) |
| MGG_09350 | 3.46 | 2.52 | 2.58 | conserved hypothetical protein (1579 nt) |
| MGG_04642 | 3.44 | 3.59 | 3.18 | predicted protein (432 nt) |
| MGG_07144 | 3.42 | 3.01 | 3.29 | hypothetical protein (673 nt) |
| MGG_09302 | 3.31 | 2.98 | 2.25 | conserved hypothetical protein (877 nt) |
| MGG_05602 | 3.28 | 2.52 | 2.53 | glucosidase 2 subunit beta (2153 nt) |
| MGG_12994 | 3.27 | 2.26 | 2.58 | conserved hypothetical protein (1313 nt) |
| MGG_11744 | 3.26 | 3.27 | 2.58 | hypothetical protein (488 nt) |
| MGG_08611 | 3.25 | 2.56 | 2.25 | conserved hypothetical protein (1662 nt) |
| MGG_06039 | 3.25 | 2.11 | 1.81 | cortical actin cytoskeleton protein asp1 (5046 nt) |
| MGG_00665 | 3.24 | 3.15 | 3.11 | tyrosyl-tRNA synthetase (2326 nt) |
| MGG_01303 | 3.18 | 3.5 | 2.72 | 3-keto-steroid reductase (1652 nt) |
| MGG_05456 | 3.17 | 2.11 | 1.63 | hypothetical protein (1009 nt) |
| MGG_05168 | 3.16 | 4.33 | 2.51 | anthranilate phosphoribosyltransferase (1278 nt) |
| MGG_00476 | 3.16 | 4.75 | 3.50 | xaa-Pro aminopeptidase 1 (2158 nt) |
| MGG_11039 | 3.13 | 2.44 | 1.39 | N-acetyltransferase 10 (3687 nt) |
| MGG_00836 | 3.07 | 4.38 | 1.65 | NUDIX domain-containing protein (652 nt) |
| MGG_02835 | 3.07 | 3.32 | 2.63 | conserved hypothetical protein (1296 nt) |
| MGG_03576 | 3.07 | 1.67 | 1.84 | conserved hypothetical protein (960 nt) |
| MGG_00525 | 3.05 | 2.15 | 2.05 | conserved hypothetical protein (1432 nt) |
| MGG_01481 | 3.04 | 3.01 | 2.74 | peroxisomal targeting signal 2 receptor (1431 nt) |
| MGG_07877 | -13.91 | -3.27 | -5.42 | dipeptidyl-peptidase V (2591 nt) |
| MGG_01944 | -13.64 | -7.86 | -13.64 | hypothetical protein (1007 nt) |
| MGG_12981 | -13.36 | -13.36 | -13.36 | cupin domain-containing protein (829 nt) |
| MGG_15113 | -12.29 | -12.29 | -5.50 | pyridoxal reductase (1494 nt) |
| MGG_12228 | -12.13 | -2.76 | -12.13 | oxidoreductase (1223 nt) |
| MGG_14269 | -11.96 | -3.59 | -2.77 | predicted protein (1252 nt) |
| MGG_12091 | -11.79 | -2.1 | -11.79 | hypothetical protein (595 nt) |
| MGG_12737 | -11.15 | -1.73 | -1.66 | creatine transporter (2080 nt) |
| MGG_00156 | -11.07 | -3.94 | -7.81 | conserved hypothetical protein (1244 nt) |
| MGG_10315 | -11.06 | -1.47 | -11.23 | hydrophobin (851 nt) |
| MGG_10961 | -10.95 | -10.95 | -10.95 | conserved hypothetical protein (1706 nt) |
| MGG_11697 | -10.63 | -3.53 | -1.84 | carbamoylphosphate synthase large subunit (1533 nt) |
| MGG_03279 | -10.47 | 2.12 | 2.32 | hypothetical protein (1901 nt) |
| MGG_15114 | -10.14 | -10.14 | -10.14 | conserved hypothetical protein (1766 nt) |
| MGG_08026 | -10.14 | -10.14 | -3.03 | hypothetical protein (690 nt) |
| MGG_12715 | -9.99 | 1.39 | -3.20 | predicted protein (740 nt) |
| MGG_02531 | -9.95 | -3.6 | -4.17 | minor extracellular protease vpr (3099 nt) |
| MGG_02273 | -9.91 | 2.5 | -9.91 | hypothetical protein (796 nt) |
| MGG_10910 | -9.91 | -9.91 | -9.91 | aflatoxin biosynthesis ketoreductase nor-1 (1322 nt) |
| MGG_12983 | -9.20 | -7.7 | -5.46 | conserved hypothetical protein (1432 nt) |
| MGG_10254 | -8.47 | -1.74 | -3.63 | taurine catabolism dioxygenase TauD (1589 nt) |
| MGG_14113 | -8.45 | -8.45 | -8.45 | hypothetical protein (518 nt) |
| MGG_10694 | -8.45 | -8.45 | -8.45 | conserved hypothetical protein (3143 nt) |
| MGG_13464 | -8.32 | -1.05 | -3.74 | laccase (2539 nt) |
| MGG_03327 | -8.29 | 2.45 | -2.80 | hypothetical protein (1920 nt) |
| MGG_05232 | -8.00 | -5.65 | -2.65 | conserved hypothetical protein (1107 nt) |
| MGG_07228 | -7.42 | -3.5 | -6.50 | conserved hypothetical protein (3698 nt) |
| MGG_02275 | -7.34 | -2.54 | -2.32 | endoprotease Endo-Pro-Aspergillus niger (1914 nt) |
| MGG_03212 | -6.72 | -2.41 | -3.46 | conserved hypothetical protein (982 nt) |
| MGG_03817 | -6.47 | -2.46 | -2.15 | metalloprotease 1 (1205 nt) |
| MGG_04729 | -6.35 | -2.26 | -1.51 | conserved hypothetical protein (1638 nt) |
| MGG_06953 | -6.21 | -1.29 | -2.32 | conserved hypothetical protein (975 nt) |
| MGG_02818 | -6.06 | -1.45 | -3.58 | isoamyl alcohol oxidase (2193 nt) |
| MGG_12982 | -5.95 | -4.45 | -3.21 | 2-(R)-hydroxypropyl-CoM dehydrogenase (1169 nt) |
| MGG_04850 | -5.73 | -3.18 | -1.97 | integral membrane protein (2312 nt) |
| MGG_03038 | -5.73 | -3.73 | -2.50 | conserved hypothetical protein (2535 nt) |
| MGG_06059 | -5.63 | -3.59 | -2.57 | arrestin (1803 nt) |
| MGG_09714 | -5.44 | -1.82 | -4.80 | saccharopepsin (2592 nt) |
| MGG_13309 | -5.41 | -3.17 | -10.27 | conserved hypothetical protein (3796 nt) |
| MGG_10245 | -5.38 | -3.46 | -2.12 | conserved hypothetical protein (1071 nt) |
| MGG_02347 | -5.32 | -6.1 | -4.75 | conserved hypothetical protein (697 nt) |
| MGG_01293 | -5.30 | -1.29 | -2.88 | conserved hypothetical protein (863 nt) |
| MGG_05344 | -5.14 | -4.81 | -3.36 | conserved hypothetical protein (759 nt) |
| MGG_05940 | -5.11 | -4.81 | -3.14 | short chain dehydrogenase (1043 nt) |
| MGG_07315 | -5.06 | -2.33 | -2.54 | predicted protein (411 nt) |
| MGG_01229 | -5.03 | -1.64 | -1.05 | conserved hypothetical protein (1327 nt) |
| MGG_12749 | -4.97 | -1.17 | -3.04 | glutathione reductase (1855 nt) |
| MGG_13929 | -4.92 | -1.71 | -2.99 | isoflavone reductase (1037 nt) |
| MGG_02127 | -4.89 | -3.8 | -2.37 | alcohol oxidase (2367 nt) |
| MGG_12316 | -4.88 | -2.48 | -4.08 | GTP-binding protein EsdC (2186 nt) |
| MGG_02157 | -4.84 | -3.66 | -4.23 | predicted protein (1245 nt) |
| MGG_01662 | -4.84 | -1.37 | -2.04 | 4-aminobutyrate aminotransferase (2102 nt) |
| MGG_08990 | -4.83 | -2 | -11.69 | predicted protein (1862 nt) |
| MGG_06769 | -4.70 | -4.63 | -2.43 | conserved hypothetical protein (1874 nt) |
| MGG_07791 | -4.63 | -5.19 | -2.21 | surface protein 1 (798 nt) |
| MGG_06747 | -4.53 | -2.43 | -4.76 | glutathione S-transferase (1469 nt) |
| MGG_09138 | -4.51 | 1.11 | -2.55 | glutathione S-transferase II (1010 nt) |
| MGG_09945 | -4.50 | -3.32 | -2.71 | averantin oxidoreductase (2371 nt) |
| MGG_00492 | -4.49 | -1.19 | -2.37 | SAM and PH domain-containing protein (3247 nt) |
| MGG_10532 | -4.40 | -1.98 | 2.20 | necrosis and ethylene inducing peptide (744 nt) |
